# Biogeography and species diversity of freshwater *Savoryellomycetidae* (*Sordariomycetes*) fungi

**DOI:** 10.1080/21501203.2025.2509809

**Published:** 2025-06-21

**Authors:** Wen-Peng Wang, Hong-Wei Shen, Dan-Feng Bao, Rajesh Jeewon, Zheng-Quan Zhang, Li-Quan Yang, Zong-Long Luo

**Affiliations:** a College of Agriculture and Biological Science, Dali University, Dali, China; b Engineering and Research Center for Southwest Biopharmaceutical Resource of National Education Ministry of China, Guizhou University, Guiyang, China; c Department of Health Sciences, Faculty of Medicine and Health Sciences, University of Mauritius, Reduit, Mauritius; d Department of Zoology, College of Science, King Saud University, Riyadh, Saudi Arabia; e College of Tea (Pu’er), West Yunnan University of Applied Sciences, Pu’er, China; f Co-Innovation Center for Cangshan Mountain and Erhai Lake Integrated Protection and Green Development of Yunnan Province, Dali University, Dali, China

**Keywords:** Ten new species, freshwater fungi, *Ascomycota*, taxonomy, biodiversity

## Abstract

The subclass *Savoryellomycetidae* (*Sordariomycetes*, *Ascomycota*) primarily consists of fungal saprobes on decayed wood from freshwater habitats. They are widely distributed worldwide with a higher diversity in Asia. This study comprehensively examines freshwater *Savoryellomycetidae* species in China, focusing on their diversity, taxonomy, phylogeny, and biogeography. A comprehensive multigene phylogenetic analysis of *Savoryellomycetidae* was performed to provide better taxonomic insights and classification of known species, resulting in the identification of 38 species, with detailed sample collection sites also provided. Among these, ten new species and three new combinations are introduced. Nine species are newly recorded in China. Both sexual and asexual morphs of *Ascolacicola aquatica* and *Phaeoisaria filiformis* are described. Additionally, some taxonomic uncertainties within existing classifications have been resolved through phylogenetic analyses and morphological characteristics. Furthermore, by integrating our data with previous studies, a biogeographic analysis of *Savoryellomycetidae* is conducted. This not only accurately depicts the number of species in this subclass but also reveals the global distribution of freshwater *Savoryellomycetidae* species. This study contributes valuable insights into the species diversity of *Savoryellomycetidae* fungi in freshwater habitats and offers a clearer understanding of the systematic relationships among taxa in this subclass.

## 1. Introduction

Fungi constitute the second largest group of eukaryotes (Tedersoo et al. 2014). However, at present, there remains a vast gap in the study of fungal diversity as most species have not yet been discovered and described (Hyde et al. 2024b; Wanasinghe et al. 2024). Freshwater fungi are an extremely complex ecological group, encompassing species that inhabit natural or artificial freshwater habitats and spend part or the whole of their life cycle in these habitats (Shearer 1993; Thomas 1996; Grossart et al. 2019). They play a vital role in nutrient recycling as decomposers and energy flow in freshwater environments (Wong et al. 1998; Wurzbacher et al. 2014; Hyde et al. 2016; Tennakoon et al. 2021; Shen et al. 2022). The subclass *Savoryellomycetidae* (*Sordariomycetes*) was introduced by Hongsanan et al. (2017), encompassing four monotypic orders, *viz*., *Conioscyphales*, *Fuscosporellales*, *Pleurotheciales*, and *Savoryellales*, with more than 30 genera (Chuaseeharonnachai et al. 2020; Hyde et al. 2020b, 2024a; Calabon et al. 2022; Yang et al. 2023a). Species of *Savoryellomycetidae* have been reported in Africa, the Americas, Asia, Europe, and Oceania, with freshwater habitats being the most prevalent, where they mainly act as saprophytes on the dead stems of different plants (Réblová et al. 2012, 2016, 2020; Yang et al. 2016; Luo et al. 2018a, 2019; Dong et al. 2021; Wang et al. 2024a). Although several studies have reported species of *Savoryellomycetidae* (Shi et al. 2021; Bao et al. 2022; Du et al. 2022; Yu et al. 2023; Xu et al. 2024a, 2024b, 2024c), no specific, systematic, and comprehensive studies have yet been conducted on this group. Additionally, research on the biogeography of these fungi remains limited. The diversity of *Savoryellomycetidae* and the global distribution patterns of freshwater species of *Savoryellomycetidae* require updated assessments. During our taxonomic investigation of lignicolous freshwater fungi in Southern China, we isolated 56 fresh strains of *Savoryellomycetidae*. Phylogenetic analysis using a combined dataset of the large subunit of nuclear ribosomal RNA gene (LSU), the nuclear ribosomal internal transcribed spacer (ITS), the small subunit of nuclear ribosomal RNA gene (SSU), the second-largest subunit of RNA polymerase II (*rpb*2), and the translation elongation factor 1-alpha (*tef*1-α) sequences revealed that these strains were identified to 38 species, including ten new species. Additionally, we compiled data on known freshwater *Savoryellomycetidae* species to map their global distribution patterns, providing valuable insights into their biogeography.

## 2. Materials and methods

### 2.1. Samples collection

Specimens of submerged decaying wood were collected from freshwater habitats (streams, rivers, and reservoirs) in Guizhou Province (wet season, August 2023), Sichuan Province (wet season, August 2024), Yunnan Province (wet season, July 2022 and 2023), Guangxi Autonomous Region (dry season, February 2024), and Xizang Autonomous Region (wet season, July 2024), China (Coordinate range: 21°96′54.90″–31°34′46.51″N; 93°32′18.73″–111°13′81.42″E). To preserve their integrity, the specimens were brought to the laboratory in plastic bags. The sample processing was performed as described by Shen et al. (2023). In brief, the samples were cut to the appropriate length, numbered, and placed in a disinfected plastic crisper for incubation at room temperature.

### 2.2. Isolation and morphological examination

Fungal colonies on natural substrates were observed using a Guiguang GL-99BI compound stereomicroscope (Guilin Guiguang Instrument Co., Ltd., Guilin, China) and then photographed with a Nikon SMZ1000 stereo zoom microscope (Nikon Corporation, Tokyo, Japan). Fungal structures were photographed using a Nikon ECLIPSE Ni-U compound microscope (Nikon Corporation, Tokyo, Japan) fitted with a Nikon DS-Ri2 digital camera (Nikon Corporation, Tokyo, Japan), as per the guidelines provided by Luo et al. (2018b). Fungal species were isolated using single spore isolation following the method described in Senanayake et al. (2020). Germinating spores were transferred to new potato dextrose agar (PDA) media and incubated at room temperature. Herbarium specimens (dry woody branches with fungal material) were deposited in the Herbarium of Cryptogams, Kunming Institute of Botany, Academia Sinica (HKAS), Kunming, China. The living cultures were deposited in the China General Microbiological Culture Collection Center (CGMCC), Beijing, China, and the Kunming Institute of Botany Culture Collection Center (KUNCC), Kunming, China. Names of the new taxa were registered in Fungal Names (FN) (https://nmdc.cn/fungalnames/, accessed on 25 January 2025). We follow Thines et al. (2020) and italicised all the Latin that appeared in the text.

### 2.3. DNA extraction, PCR amplification, and sequencing

Genomic DNA was extracted from fungal mycelium. A Trelief^TM^ Hi-Pure Plant Genomic DNA Kit (Beijing TsingKe Biotech Co., Ltd., Beijing, China) was used to extract total genomic DNA following the manufacturer’s instructions. DNA amplification was performed by the polymerase chain reaction (PCR). LSU, ITS, SSU, *rpb*2, and *tef*1-α gene regions were amplified using the primer pairs LR0R/LR5 (Vilgalys and Hester 1990), ITS5/ITS4 (White et al. 1990), NS1/NS4 (White et al. 1990), fRPB2-5F/fRPB2-7cR (Liu et al. 1999), and 983F/2218 R (Rehner and Buckley 2005). The amplifications were performed in a 25 µL reaction volume containing 9.5 µL ddH_2_O, 12.5 µL 2× Taq PCR Master Mix with blue dye (Shanghai Biotech Bioengineering Co., Ltd., Shanghai, China), 1 µL of DNA template, and 1 µL of each primer (10 µmol/L). PCR products were checked on 1% agarose electrophoresis gels stained with Gel Red (Beijing Tsingke Biological Engineering Technology and Services Co., Ltd., Beijing, China). The sequencing reactions were carried out with the primers mentioned above by Beijing Tsingke Biological Engineering Technology and Services Company, Beijing, China, or Shanghai Sangon Biological Engineering Technology and Services Co., Shanghai, China.

### 2.4. Phylogenetic analyses

The BLAST searches in National Center of Biotechnology Information (NCBI) were initially performed to screen out strains of *Savoryellomycetidae* for further analyses. Five gene markers LSU, ITS, SSU, *rpb*2, and *tef*1-α were used for the multi-gene analyses with the whole or part of them concatenated for different fungal groups (Table S1). Single-locus sequences were aligned using the online multiple alignment programme MAFFT v7 (Katoh and Standley 2013) and sequence trimming was performed with trimAl v1.2 for Windows (http://trimal.cgenomics.org), with 0.5 site coverage cut-off value (Capella-Gutiérrez et al. 2009). BioEdit was used to manually adjust the alignments and the alignment fasta file (http://www.mbio.ncsu.edu/bioedit/bioedit.html). The concatenated sequence alignments were obtained from SequenceMatrix v1.7.8 (Vaidya et al. 2011).

Maximum likelihood (ML) analysis was performed using RAxML-HPC2 on ACCESS (Stamatakis 2006; Stamatakis et al. 2008) on the XSEDE Teragrid of the CIPRES Science Gateway online platform (Miller et al. 2010) with rapid bootstrap analysis, followed by 1,000 bootstrap replicates, using the GTRGAMMA+I model of evolution.

Bayesian inference (BI) analysis was performed in a likelihood framework implemented in MrBayes v3.1.2 (Ronquist et al. 2012). Best-fit model of DNA evolution for the Bayesian inference analysis was estimated by MrModeltest v2.2 (Nylander 2004), and the GTR+I+G model was selected for LSU, ITS, *rpb*2, and *tef*1-α, GTR+G model was selected for SSU. The Markov Chain Monte Carlo (MCMC) sampling approach was used to calculate posterior probability (PP) (Rannala and Yang 1996). Bayesian analysis of six simultaneous Markov chains was run for 10,000,000 generations, with trees sampled every 1,000 generations. Sequences generated in this study were deposited in GenBank and are listed in Table S1.

### 2.5. Collection and processing of data

By using information from Index Fungorum (http://www.indexfungorum.org/Names/Names.asp, accessed on 31 December 2024) and published studies, we determined the total number of species in *Conioscyphales*, *Fuscosporellales*, *Pleurotheciales*, and *Savoryellales* as well as the number of species reported from freshwater habitats. We then compiled all data as shown in Figure 37 and listed all species of freshwater *Savoryellomycetidae* in Table S2, which includes hosts, environments, and reported countries and continents. Moreover, we constructed a double-layer donut chart, which better demonstrates the percentage of freshwater *Savoryellomycetidae* in different freshwater environments and continents.

## 3. Results

### 3.1. Phylogenetic analyses

The dataset of combined LSU, ITS, SSU, *rpb*2, and *tef*1-α sequence data comprises 356 strains with 3,989 characters, including gaps (LSU: 1–801 bp, ITS: 802–1,315 bp, SSU: 1,316–2,280 bp, *rpb*2: 2,281–3,159 bp, and *tef*1-α: 3,160–3,989 bp). *Pisorisporium cymbiforme* (PRM 924377 and PRM 924379) were selected as the outgroup taxa. RAxML and Bayesian analyses were conducted and resulted in generally congruent topologies. The best RAxML tree with a final likelihood value of − 88,336.078716 is presented. The matrix had 2,656 distinct alignment patterns, with 40.52% undetermined characters or gaps. Estimated base frequencies were as follows: A = 0.232115, C = 0.264461, G = 0.292306, T = 0.211117; substitution rates AC = 1.298126, AG = 3.080387, AT = 1.382528, CG = 1.108979, CT = 6.392586, and GT = 1.000000; the gamma distribution shape parameter was α = 0.343651.

In the phylogenetic tree, 56 new collections were nested in 14 different genera of *Savoryellomycetidae*. Of these, 34 new collections clustered with *Dematipyriforma*, *Phaeoisaria*, *Pleurotheciella*, *Pleurothecium*, and *Sterigmatobotrys* of *Pleurotheciales*; fourteen new collections clustered with *Ascolacicola*, *Bactrodesmium*, *Canalisporium*, *Dematiosporium*, and *Savoryella* of *Savoryellales*; a new collection clustered with *Conioscyphales*; and seven new collections clustered with *Fuscosporella*, *Mucispora*, and *Vanakripa* of *Fuscosporellales* (Figure 1).

**Figure 1.**
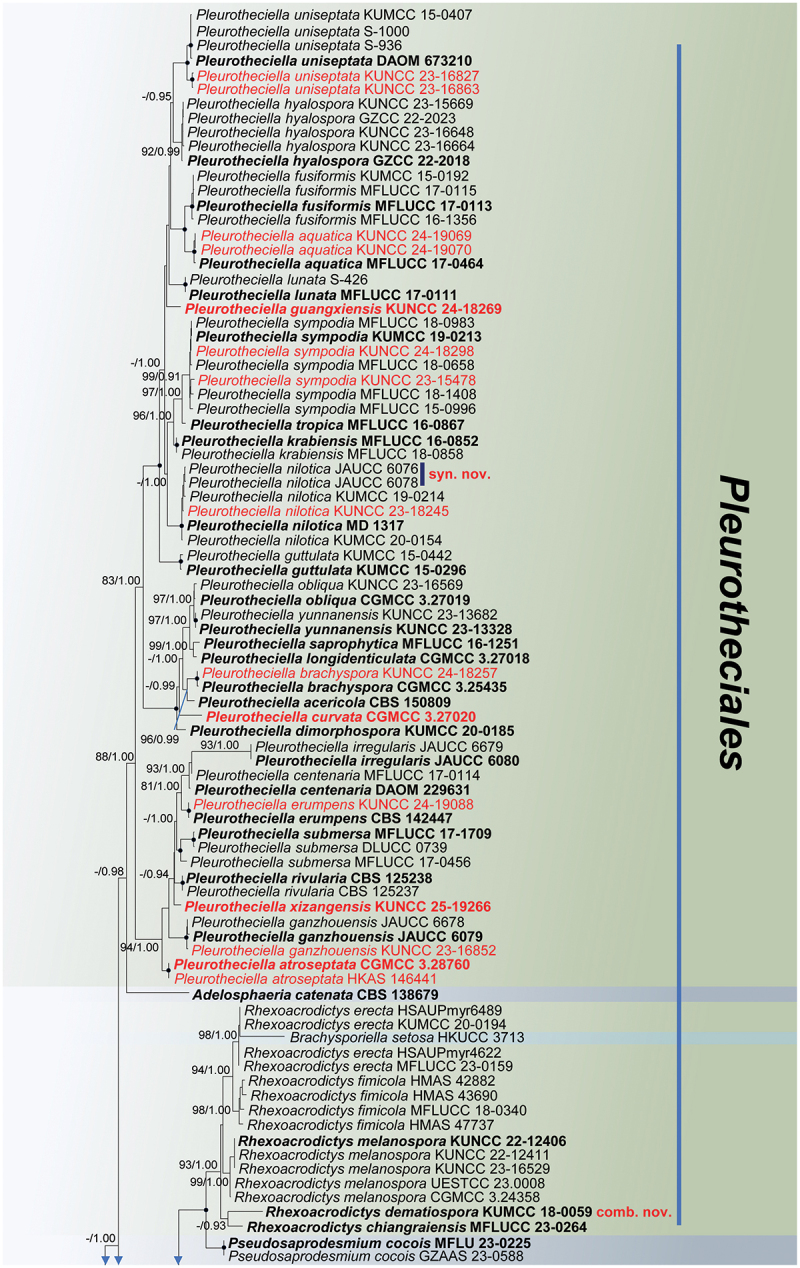
RAxML tree based on combined LSU, ITS, SSU, *rpb*2, and *tef*1-α sequence data of *Savoryellomycetidae*. Bootstrap support values for maximum likelihood (ML) greater than 80% and Bayesian posterior probabilities (PP) greater than 0.90 are given as ML/PP above the nodes. Newly obtained sequences are indicated in red, and ex-type strains are in bold. The black dot indicates that branches with 100% ML/1.00 PP support.

**Figure 1.**
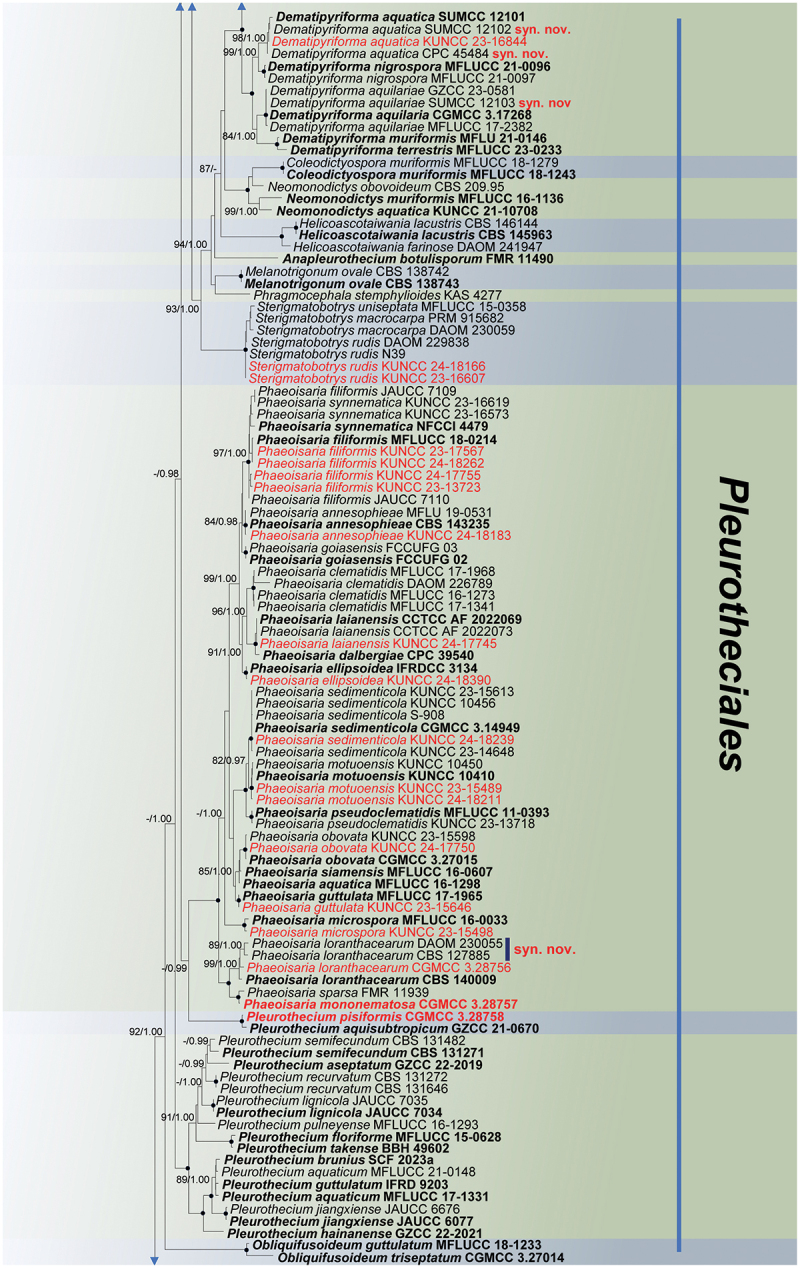
(Continued).

**Figure 1.**
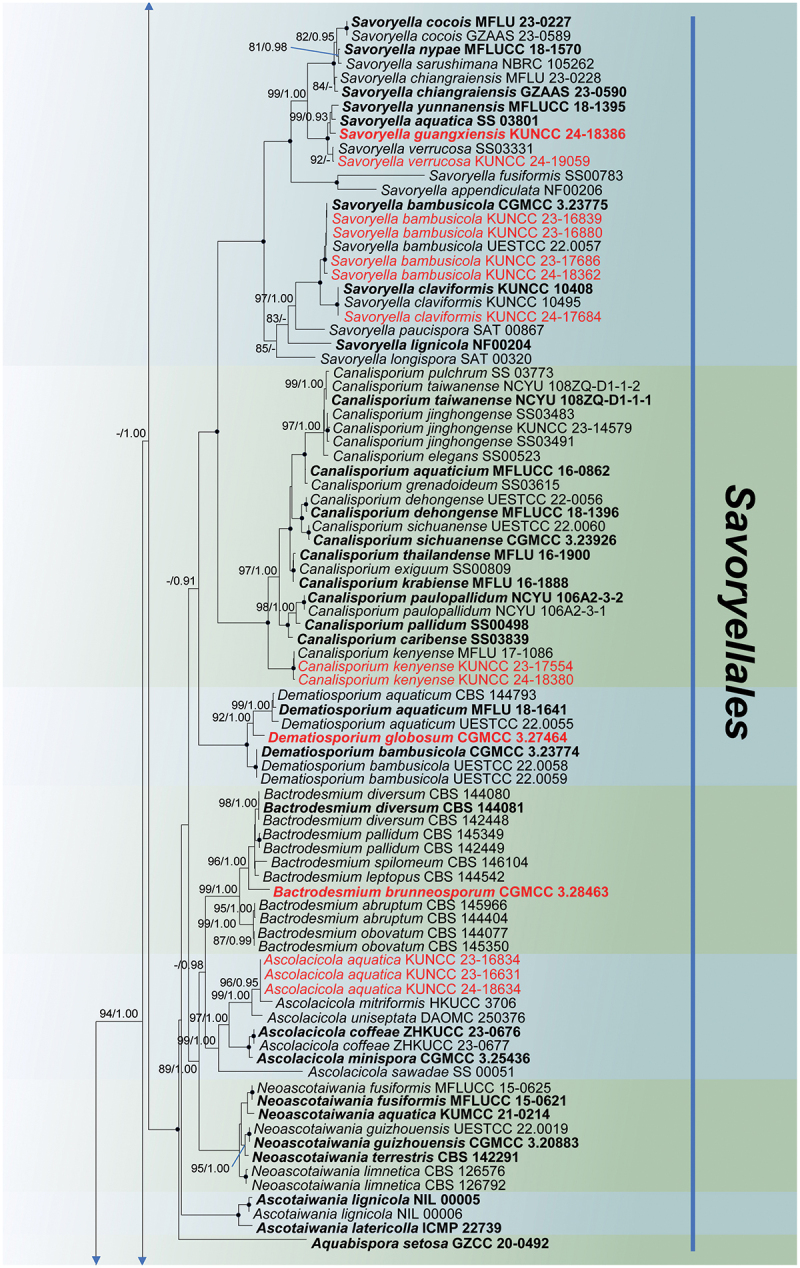
(Continued).

**Figure 1.**
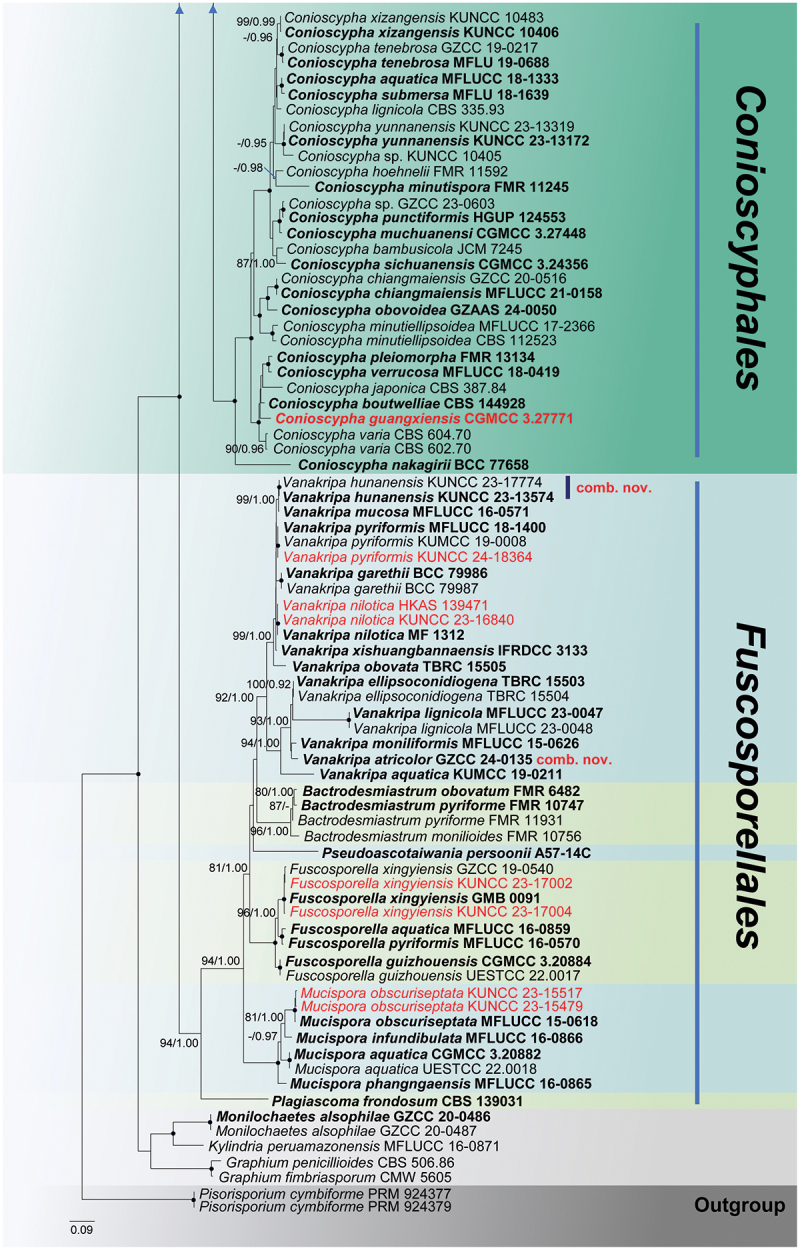
(Continued).

### 3.2. Taxonomy

***Sordariomycetes*** O.E. Erikss. & Winka

***Savoryellomycetidae*** Hongsanan, K.D. Hyde & Maharachch.

***Conioscyphales*** Réblová & Seifert

***Conioscyphaceae*** Réblová & Seifert

Notes: *Conioscyphaceae* was introduced by Réblová et al. (2016) to accommodate the monotypic genus *Conioscypha*, so far, the family comprises only one genus (Hyde et al. 2024a). In earlier studies, *Conioscypha* included asexual species with dark brown, globose to subglobose conidia (von Höhnel 1904; Shearer and Motta 1973; Goh and Hyde 1998). Réblová and Seifert (2004) established *Conioscyphascus* to accommodate two sexual species, *C. gracilis* and *C. varius*. Subsequently, *Conioscyphascus* was synonymised under *Conioscypha* based on cultural studies of *C. varius* and phylogenetic analysis of DNA sequence data (Zelski et al. 2015; Réblová et al. 2016).

***Conioscypha*** Höhn.

Notes: *Conioscypha* was introduced by von Höhnel (1904), with *C. lignicola* as the type. Twenty-seven species are accepted in *Conioscypha*, and most species are reported as asexual morphs. The asexual morph of *Conioscypha* is characterised by reduced or micronematous, hyaline conidiophores with terminal, enteroblastic or phialidic conidiogenous cells, and subglobose or elliptical, pigmented conidia (Hernández-Restrepo et al. 2017; Luo et al. 2019; Phukhamsakda et al. 2022; Li et al. 2024; Xu et al. 2024c; Yu et al. 2024a, 2024b). The sexual morph of *Conioscypha* is characterised by globose to subglobose, immersed to superficial ascomata, cylindric-clavate asci with a refractive J- apical annulus, and fusiform, multiseptate ascospores (Réblová and Seifert 2004). *Conioscypha* species are frequently reported from freshwater and terrestrial habitats on decayed plant tissues (Luo et al. 2019; Hyde et al. 2020a; Li et al. 2024; Xu et al. 2024c; Yu et al. 2024a, 2024b). In this study, we introduce a new species, *Conioscypha guangxiensis*, from freshwater habitat in Guangxi, China.

**Figure 2.**
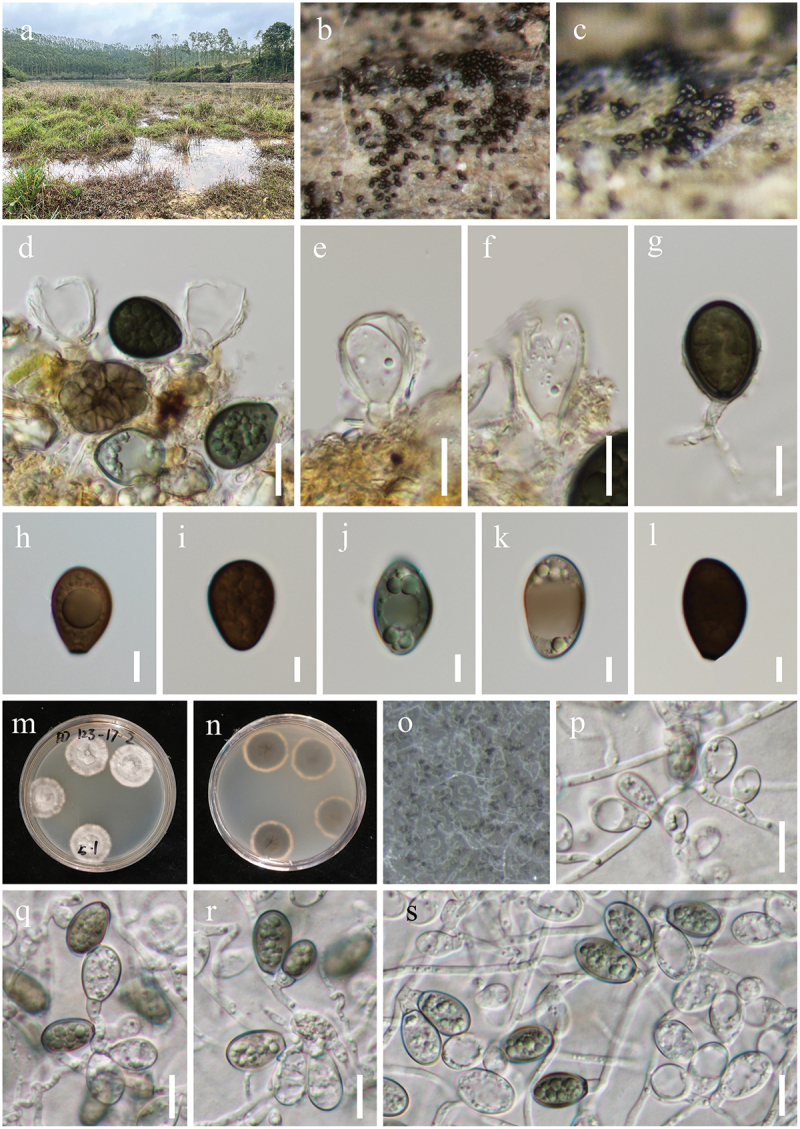
*Conioscypha guangxiensis* (HKAS 139430, holotype). (a) Freshwater habitat. (b, c) Colonies on the substratum. (d–g) Conidiogenous cells with conidia. (h–l) Conidia. (m, n) Colonies on PDA from surface and reverse. (o–s) Sporulation observed on PDA [(o) Colonies on PDA; (p–s) Conidiogenous cells with conidia]. Scale bars: d–g, p–s = 10 µm, h–l = 5 µm.

***Conioscypha guangxiensis*** W.P. Wang & Z.L. Luo, sp. nov. Figure 2

*Fungal Names number*: FN572353.

*Etymology*: Referring to the Guangxi Autonomous Region, China, where the species was collected.

*Holotype*: HKAS 139430.

*Saprobic* on submerged decaying wood. **Asexual morph**: *Colonies* superficial, effuse, dark brown to black conidia gathered or scattered, glistening. *Mycelium* immersed, composed of septate, smooth, branched, hyaline hyphae. *Conidiophores* reduced or mononematous, micronematous, hyaline, cylindrical. *Conidiogenous cells* 4.4–5.6 × 2.7–3.3 µm, monoblastic, integrated, terminal, determinate, hyaline. *Conidia* 15–22 × 10–16 µm ($\overset{ˉ}{x}$ = 17.7 × 12.7 µm, *n* = 30), acrogenous, solitary, ellipsoidal to obovoid or subglobose, aseptate, smooth-walled, brown or patina when young, dark brown when mature, broad, rounded apical and basal or truncate at the base, guttulate. **Sexual morph**: Undetermined.

Culture characteristics: Colonies on PDA reaching 30 mm in diameter after 3 weeks at room temperature. Mycelia dry, dense. Colonies on the surface of PDA, with regular edges, slightly protruding in centre, surface rough, white. Reverse circular, sectored from the centre, dark brown with white edges. *Conidiophores* reduced. *Conidiogenous cells* 3–7.9 × 2.5–4.6 µm, monoblastic, integrated, terminal, determinate, cylindrical, hyaline, guttulate. *Conidia* 13–19 × 8.1–11 µm ($\overset{ˉ}{x}$ = 15.6 × 9.5 µm, *n* = 50), acrogenous, solitary, obovoidal, aseptate, smooth-walled, hyaline to greyish green, broad, rounded apical and truncate base, guttulate.

Material examined: China, Guangxi Autonomous Region, Qinzhou, Hongchaojiang reservoir (21°96′54.90″N; 109°07′44.86″E), on unknown submerged decaying wood, 25 February 2024, Fa-Li Li, S-6424 (HKAS 139430, holotype), ex-type cultures, CGMCC 3.27771 = KUNCC 24-18299.

Notes: The BLAST search of the ITS sequence of *Conioscypha guangxiensis* on NCBI GenBank showed that the most similar is *C. varia*, with a 93.05% similarity rate. Comparisons of the LSU and ITS sequences of *C. guangxiensis* compared with *C. varia* (CBS 602.70) showed a difference of 1.69% (14/826 bp, five gaps) and 5.1% (25/490 bp, 19 gaps) across the LSU and ITS nucleotides. In addition, *C. guangxiensis* differs from *C. varia* in having cylindrical conidiophores, terminal, monoblastic conidiogenous cells, and acrogenous, guttulate conidia (Figure 2). We follow the guidelines of Jeewon and Hyde (2016) to introduce *C. guangxiensis* as a new species.

***Fuscosporellales*** J. Yang, J. Bhat & K.D. Hyde

***Fuscosporellaceae*** J. Yang, J. Bhat & K.D. Hyde

Notes: Yang et al. (2016) established *Fuscosporellaceae* to accommodate *Bactrodesmiastrum* species, *Plagiascoma frondosum*, and four new genera, *viz*., *Fuscosporella*, *Mucispora*, *Parafuscosporella*, and *Pseudoascotaiwania*, with *Fuscosporella* as the type genus. Recently, Goh et al. (2023, 2024) transferred all *Parafuscosporella* species to *Vanakripa*, and synonymised *Parafuscosporella* under *Vanakripa* based on phylogenetic analysis and morphological characteristics. To date, six genera, *viz. Bactrodesmiastrum*, *Fuscosporella*, *Mucispora*, *Plagiascoma*, *Pseudoascotaiwania*, and *Vanakripa* are accepted in *Fuscosporellaceae*. The sexual morph of *Fuscosporellaceae* is characterised by immersed to semi-immersed, dark brown to black ascomata with fragile peridium, unitunicate, 8-spored, cylindrical to cylindric-fusiform, stipitate asci with a non-amyloid apical ring, and fusiform, hyaline or light brown, transversely septate ascospores (Réblová et al. 2016; Yang et al. 2016). *Bactrodesmiastrum*, *Fuscosporella*, *Mucispora*, and *Vanakripa* are reported only in the asexual morph stage, characterised by micronematous, macronematous conidiophores mostly reduced, holoblastic, hyaline, obovate to globose conidiogenous cells, and obovate to obpyriform, dark conidia (Yang et al. 2016, 2020; Du et al. 2022; Goh et al. 2023).

***Fuscosporella*** J. Yang, J. Bhat & K.D. Hyde

Notes: *Fuscosporella* was introduced by Yang et al. (2016), with *F. pyriformis* as the type. *Fuscosporella* species are often saprobes on dead stems from freshwater habitats (Yang et al. 2016, 2017, 2023a; Du et al. 2022). Xu et al. (2022) introduced *F. xingyiensis* from terrestrial habitat in Guizhou Province, China. *Fuscosporella* is characterised by sporodochia colonies, conidiophores absent, originating from superficial hyphae, intercalary, moniliform conidiogenous cells, and thallic, developing intercalarily from sporogenous hyphae, muriform, ellipsoidal conidia with or without dilated appendages at both ends (Goh et al. 2024).

***Fuscosporella xingyiensis*** X. Xu, Q.R. Li, Y.Q. Kang & Wijayaw., Chiang Mai J. Sci. 49 (3): 720 (2022), Figure 3

**Figure 3.**
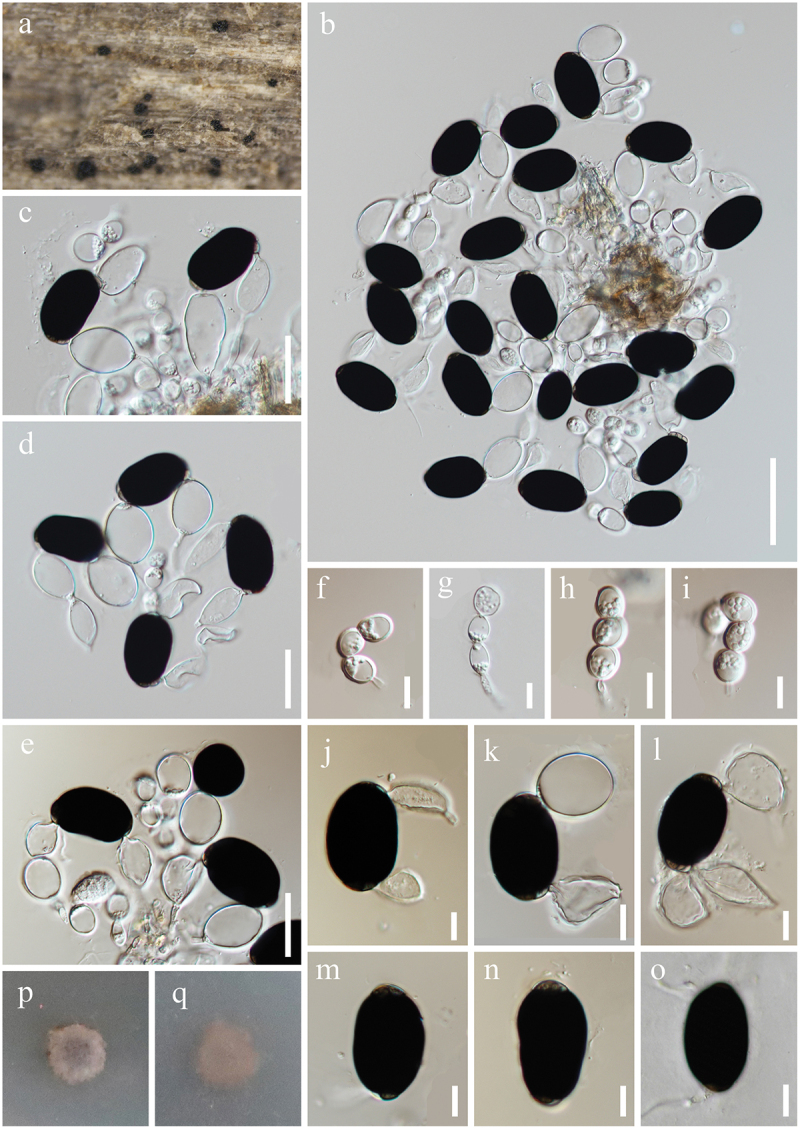
*Fuscosporella xingyiensis* (HKAS 139436). (a) Colonies on the substratum. (b–e) Conidiogenous cells with conidia. (f–i) Conidiogenous cells. (j–n) Conidia. (o) Germinating conidium. (p, q) Colony on PDA from surface and reverse. Scale bars: b = 50 µm, c–e = 30 µm, f–o = 10 µm.

*Fungal Names number*: FN555821.

Material examined: China, Guizhou Province, Qiandongnan Autonomous Prefecture, Zhenyuan County (27°03′13.43″N; 108°25′79.11″E), on unknown submerged decaying wood in a freshwater stream, 17 August 2023, Sha Luan, S-5800 (HKAS 139436), living culture, KUNCC 23-17002; *ibid*., S-5802 (HKAS 139446), living culture, KUNCC 23-17004.

***Mucispora*** J. Yang, J. Bhat & K.D. Hyde

Notes: *Mucispora* was introduced by Yang et al. (2016), with *M. obscuriseptata* as the type species. *Mucispora* can be clearly distinguished from other asexual genera of *Fuscosporellaceae* in having macronematous, mononematous, erect, cylindrical conidiophores. All *Mucispora* species have been reported on submerged decaying wood in China and Thailand (Yang et al. 2016, 2017; Hyde et al. 2020b; Wijayawardene et al. 2021; Du et al. 2022).

***Mucispora obscuriseptata*** J. Yang, J. Bhat & K.D. Hyde, Cryptogam. Mycol. 37 (4): 466 (2016), Figure 4

**Figure 4.**
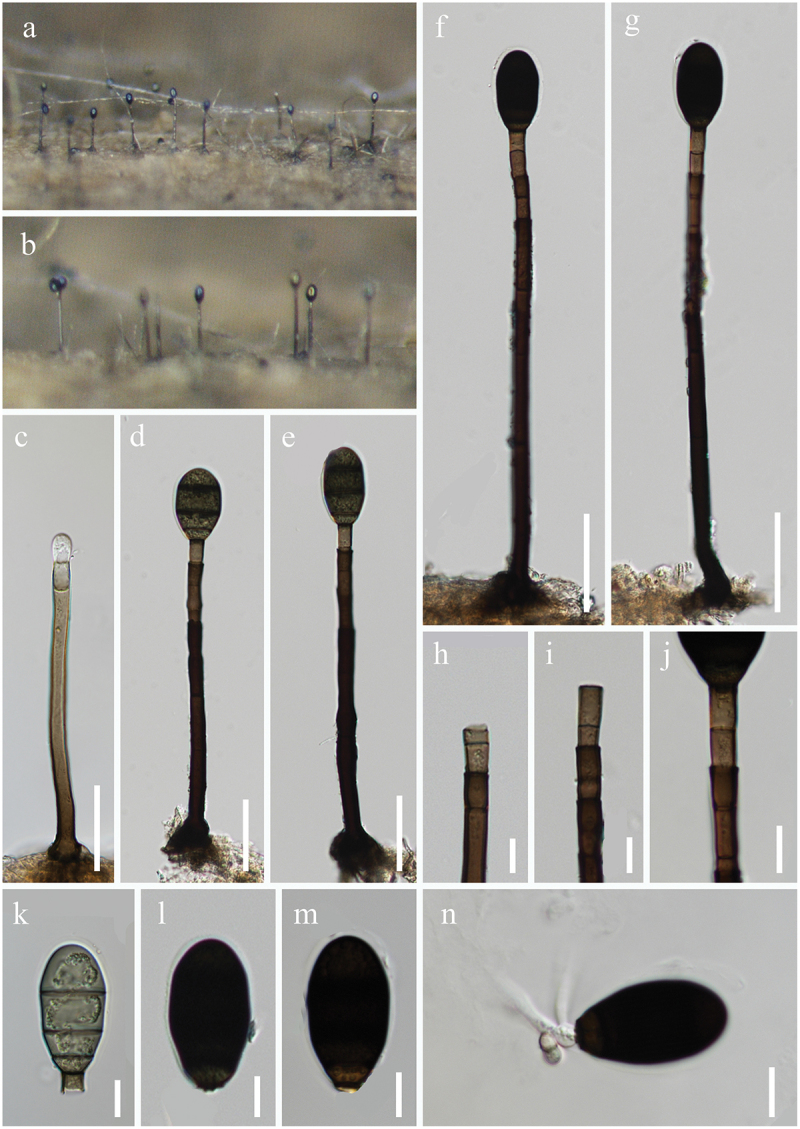
*Mucispora obscuriseptata* (HKAS 139469). (a, b) Colonies on the substratum. (c–g) Conidiophores with conidia. (h–j) Conidiogenous cells. (k–m) Conidia. (n) Germinating conidium. Scale bars: c–e = 30 µm, f, g = 40 µm, h–n = 10 µm.

*Fungal Names number*: FN552291.

*Saprobic* on submerged decaying wood. **Asexual morph**: *Colonies* erect, hairy, scattered, solitary, brown conidiophores with a glistening, black conidia. *Mycelium* partly immersed, partly superficial, composed of branched, septate, brown hyphae. *Conidiophores* 111–200 × 5–7.3 µm ($\overset{ˉ}{x}$ = 152.9 × 6.1 µm, *n* = 20), macronematous, mononematous, solitary, cylindrical, straight or slightly flexuous, smooth, thick-walled, dark brown, slightly paler towards the tip, percurrent proliferating. *Conidiogenous cells* 6.2–14 × 5–7 µm ($\overset{ˉ}{x}$ = 9.1 × 5.7 µm, *n* = 20), monoblastic, integrated, terminal, cylindrical, smooth-walled, brown. *Conidia* 31–37 × 16–24 µm ($\overset{ˉ}{x}$ = 34.8 × 19.2 µm, *n* = 20), acrogenous, solitary, ellipsoidal to obovoidal, olive green when young, dark brown to black when mature, with a paler cell at the base, 3-septate, rounded apical and truncate base, smooth-walled, partly covered by a hyaline, thin mucilaginous sheath. **Sexual morph**: Undetermined.

Culture characteristics: Conidia germinating on PDA within 24 h, with germ tubes produced from the base.

Material examined: China, Yunnan Province, Xishuangbanna Autonomous Prefecture, Xishuangbanna Tropical Botanical Garden (21°53′37.60″N; 101°16′30.98″E), on unknown submerged decaying wood in a freshwater stream, 12 July 2022, Rui Gu, S-4203 (HKAS 139469), living culture KUNCC 23-15517; *ibid*., S-4267 (HKAS 139462), living culture, KUNCC 23-15479.

Notes: Our new collections fit well with the description of *Mucispora obscuriseptata* in having erect, cylindrical conidiophores percurrently proliferating, and ellipsoidal to obovoidal, 3-septate conidia with a hyaline mucilaginous sheath (Yang et al. 2016). Comparisons of LSU, ITS, SSU, and *rpb*2 gene regions of the new collection (KUNCC 23-15517) and the type strain of *M. obscuriseptata* (MFLUCC 15-0618) showed a similarity of 99.88% (833/834 bp), 99.28% (552/556 bp, three gaps), 100.00% (997/997 bp), and 99.19% (861/868 bp), respectively. We therefore identify our new collections as *M. obscuriseptata* based on morphological characteristics and sequence data. *Mucispora obscuriseptata* was introduced by Yang et al. (2016) from freshwater stream in Thailand, and it is reported for the first time in China.

***Vanakripa*** Bhat, W.B. Kendr. & Nag Raj

Notes: *Vanakripa* was introduced by Bhat and Kendrick (1993), with *V. gigaspora* as the type species. Since the type species do not have molecular data, some vanakripa-like species were accepted in *Vanakripa* (Pinnoi et al. 2003; Yang et al. 2020, 2023a; Phukhamsakda et al. 2022). Goh et al. (2023) considered that *Parafuscosporella* fits well with the generic circumscription of *Vanakripa*, and synonymised *Parafuscosporella* under *Vanakripa*, and accepted 13 species in *Vanakripa*. All *Vanakripa* species are reported as asexual morphs, which are characterised by sporodochia conidiomata with or without jelly-like, gelatinous covering, unbranched or branched, hyaline conidiophores composed of globose to subglobose, cylindrical or clavate, sometimes moniliform cells, monoblastic conidiogenous cells, and obovoidal or pyriform with broadly apical, uniseptate at the base, distal cell dark brown to black, proximal cell brown, smooth-walled conidia (Boonyuen et al. 2016, 2021; Yang et al. 2016, 2020; Boonmee et al. 2021; Goh et al. 2023; Li et al. 2023; Wang et al. 2023).

***Vanakripa atricolor*** (N.G. Liu, K.D. Hyde & Jian K. Liu) W.P. Wang & Z.L. Luo, comb. nov.

*Fungal Names number*: FN572468.

*Basionym*: ***Parafuscosporella atricolor*** N.G. Liu, K.D. Hyde & Jian K. Liu, Fungal Divers. 129: 157 (2024).

Holotype: China, Guizhou Province, Xiaochehe Wetland Park, on decaying submerged wood, 24 July 2018, Ning-Guo Liu, XCH016 (GZAAS 24-0043, holotype), ex-type culture GZCC 24-0135.

Notes: Phylogenetic analysis showed that *Parafuscosporella atricolor* and *P. hunanensis* clustered within *Vanakripa* (Figure 1), and both species also fit the concept of *Vanakripa* (Liu et al. 2024a; Li et al. 2025). We therefore transfer *P. atricolor* and *P. hunanensis* to *Vanakripa*.

***Vanakripa hunanensis*** (L. Li, Bhat & Phookamsak) W.P. Wang & Z.L. Luo, comb. nov.

*Fungal Names number*: FN572469.

*Basionym*: ***Parafuscosporella hunanensis*** L. Li, Bhat & Phookamsak, Front. Cell. Infect. Microbiol. 14(no. 1515972): 11 (2025).

Holotype: China, Hunan Province (108°47′–114°15′E, 24°38′–30°08′N, 500–1,500 msl), saprobic on decaying wood submerged in a freshwater stream, 26 August 2022, L. Li, LILU 203 (HKAS 136260, holotype), ex-type culture, KUNCC23-13574; *ibid*., LILU-203-2 (HKAS 136261), living culture KUNCC 24-17774.

Notes: *Parafuscosporella hunanensis* was introduced by Li et al. (2025) from a freshwater habitat in Hunan Province, China. Based on phylogenetic analysis and morphological characteristics, *Vanakripa hunanensis* comb. nov. is introduced in this study.

***Vanakripa nilotica*** (Abdel-Aziz) Goh, S.Y. Hsieh & C.H. Kuo, Fungal Divers. 111: 176 (2021), Figure 5

**Figure 5.**
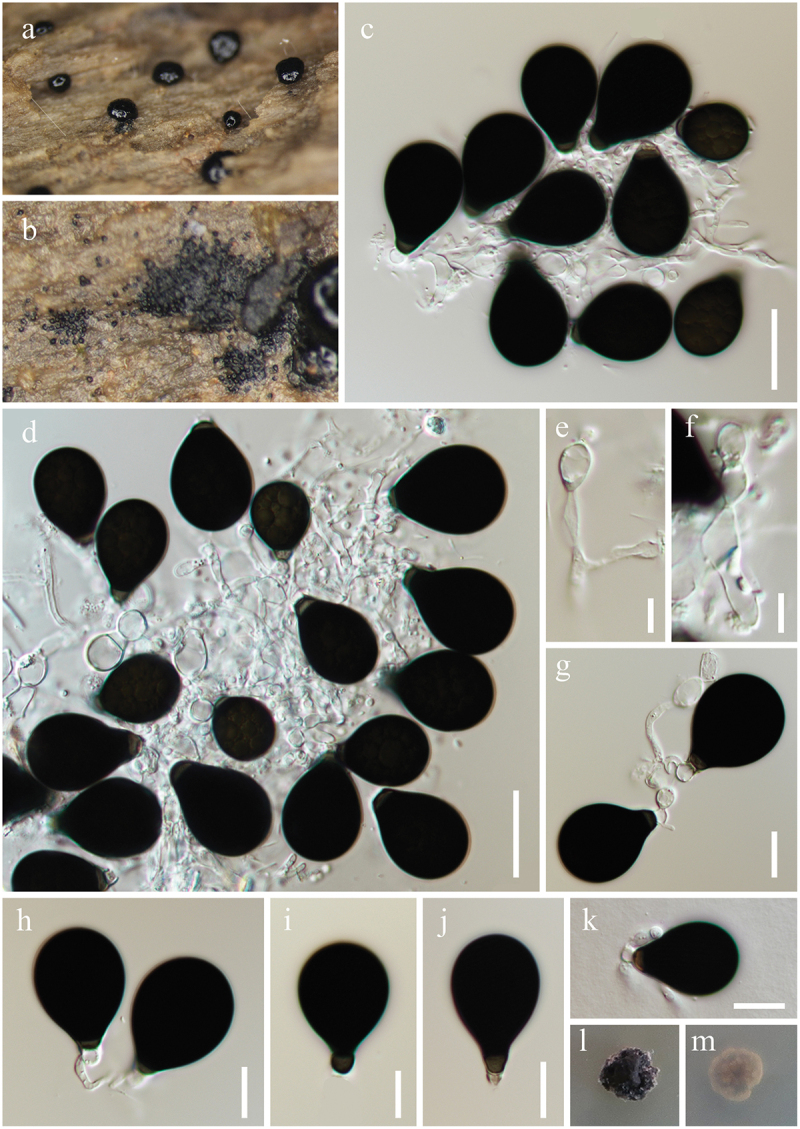
*Vanakripa nilotica* (HKAS 139439). (a, b) Colonies on the substratum. (c, d) Amount of conidia. (e) Conidia born directly on hyphae. (f) Conidiophore. (g, h) Hyphae with conidia. (i, j) Conidia. (k) Germinating conidium. (l, m) Colony on PDA from surface and reverse. Scale bars: c, d = 20 µm, e, f = 5 µm, g–k = 10 µm.

*Fungal Names number*: FN848839.

*Saprobic* on submerged decaying wood. **Asexual morph**: *Colonies* superficial, sporodochia, black, with jelly-like, gelatinous covering. *Mycelium* mostly immersed, composed of branched, septate, hyaline hyphae. *Conidiophores* reduced or micronematous, mononematous, subcylindrical composed of obovoid cells, smooth, thin-walled, hyaline. *Conidiogenous cells* monoblastic, integrated, terminal, hyaline. *Conidia* 20–31 × 14–22 µm ($\overset{ˉ}{x}$ = 23.8 × 17 µm, *n* = 30), acrogenous, partly directly produced from hyphae, obovoidal to pyriform, broadly rounded at the apex, uniseptate at the base, smooth-walled, distal cell dark brown to black, proximal cell brown, guttulate, sometimes with a globose to subglobose, hyaline separating cell. **Sexual morph**: Undetermined.

Cultural characteristics: Conidia germinating on PDA within 36 h, with germ tubes produced from the base. Colonies on PDA reaching 8 mm in diameter after 6 weeks at room temperature. Mycelia dry, dense. Colonies on the surface of PDA, with irregular edges, protrusions, surface rough, dark brown, pale brown to brown from below.

Material examined: China, Yunnan Province, Wenshan Autonomous Prefecture, Qiubei County, Xiaohuishan reservoir (24°16′24.33″N; 104°13′77.45″E), on unknown submerged decaying wood, 17 July 2023, Zheng-Quan Zhang, S-5847 (HKAS 139439), living culture, KUNCC 23-16840; *ibid*., S-5982 (HKAS 139471).

Notes: Boonmee et al. (2021) introduced *Parafuscosporella nilotica*, collected from Nile River in Egypt, but Goh et al. (2023) transferred *P. nilotica* to *Vanakripa* as *V. nilotica*. In the phylogenetic analysis, two new collections clustered with the ex-type strain of *V. nilotica* with 100% ML/1.00 PP support (Figure 1). Our new collections are similar to *V. nilotica* in having sporodochia conidiomata with jelly-like, gelatinous covering, micronematous, mononematous or reduced conidiophores, sometimes directly produced from hyphae, obovoidal to pyriform, broadly rounded at the apex conidia with a septate at the base (Boonmee et al. 2021). We therefore identify our new collections as *V. nilotica*, and it is reported in China for the first time.

***Vanakripa pyriformis*** (H. Yang, W. Dong & H. Zhang) Goh, S.Y. Hsieh & C.H. Kuo, Mycol. Prog. 22: 61 (2023)

*Fungal Names number*: FN848839.

Material examined: China, Yunnan Province, Wenshan Autonomous Prefecture, Qiubei County, Xiaohuishan reservoir (24°16′24.33″N; 104°13′77.45″E), on unknown submerged decaying wood, 17 July 2023, Xing-Ya Zeng, S-5639 (HKAS 139432), living culture, KUNCC 23-18364.

***Pleurotheciales*** Réblová & Seifert

***Pleurotheciaceae*** Réblová & Seifert

Notes: *Pleurotheciaceae* was introduced by Réblová et al. (2016), with *Pleurothecium* as the type genus. It is the largest family within *Savoryellomycetidae*, Hyde et al. (2024a) listed 15 genera, *viz*., *Adelosphaeria*, *Anapleurothecium*, *Coleodictyospora*, *Dematipyriforma*, *Helicoascotaiwania*, *Melanotrigonum*, *Monotosporella*, *Neomonodictys*, *Phaeoisaria*, *Phragmocephala*, *Pleurotheciella*, *Pleurothecium*, *Pseudosaprodesmium*, *Saprodesmium*, and *Sterigmatobotrys* in this family. However, phylogenetic analyses of this study and other recent studies showed that *Rhexoacrodictys* is nested within *Pleurotheciaceae* (Bao et al. 2022; Huang et al. 2022; Tian et al. 2024; Wang et al. 2024a; Win et al. 2024). Additionally, Wang et al. (2024a) accepted *Obliquifusoideum* into this family. Therefore, 17 genera in this family were accepted in this study. Species of *Pleurotheciaceae* are widely distributed in terrestrial, freshwater and marine habitats across the globe, with freshwater habitat being the most prevalent (Wang et al. 2024a). In freshwater habitats, *Pleurotheciaceae* species are mainly saprophytic on the dead stem tissues of various plant species (Réblová 2012; 2016; Luo et al. 2018a, 2019; Hyde et al. 2018, 2020b; Shi et al. 2021).

***Dematipyriforma*** L.Y. Sun, H.Y. Li, X. Sun & L.D. Guo

Notes: *Dematipyriforma* was introduced by Sun et al. (2017) to accommodate the type *D. aquilaria*. *Dematipyriforma* is characterised by reduced or micronematous to semi-macronematous, fasciculate, hyaline, cylindrical conidiophores, monoblastic, intercalary or terminal conidiogenous cells, and pyriform or ellipsoidal to obovoidal, muriform conidia with transverse and oblique or longitudinal septation (Sun et al. 2017; Bao et al. 2022; Jayawardena et al. 2022). At present, eight species are recognised in *Dematipyriforma*, and these species exhibit diverse lifestyles. *Dematipyriforma aquatica*, *D. globispora*, *D. muriformis*, *D. nigrospora*, *D. nilotica*, and *D. terrestris* are saprobes on dead wood or leaf; *D. aquilaria* is an endophytic species isolated from *Aquilaria crassna*, and *D. americana* has been isolated from a swab of the basement wall (Bao et al. 2022; Tian et al. 2024; Crous et al. 2024; Samarakoon et al. 2024).

***Dematipyriforma aquatica*** Abdel-Aziz & Abdel-Wahab, Fungal Divers. 117: 132 (2022), Figure 6

**Figure 6.**
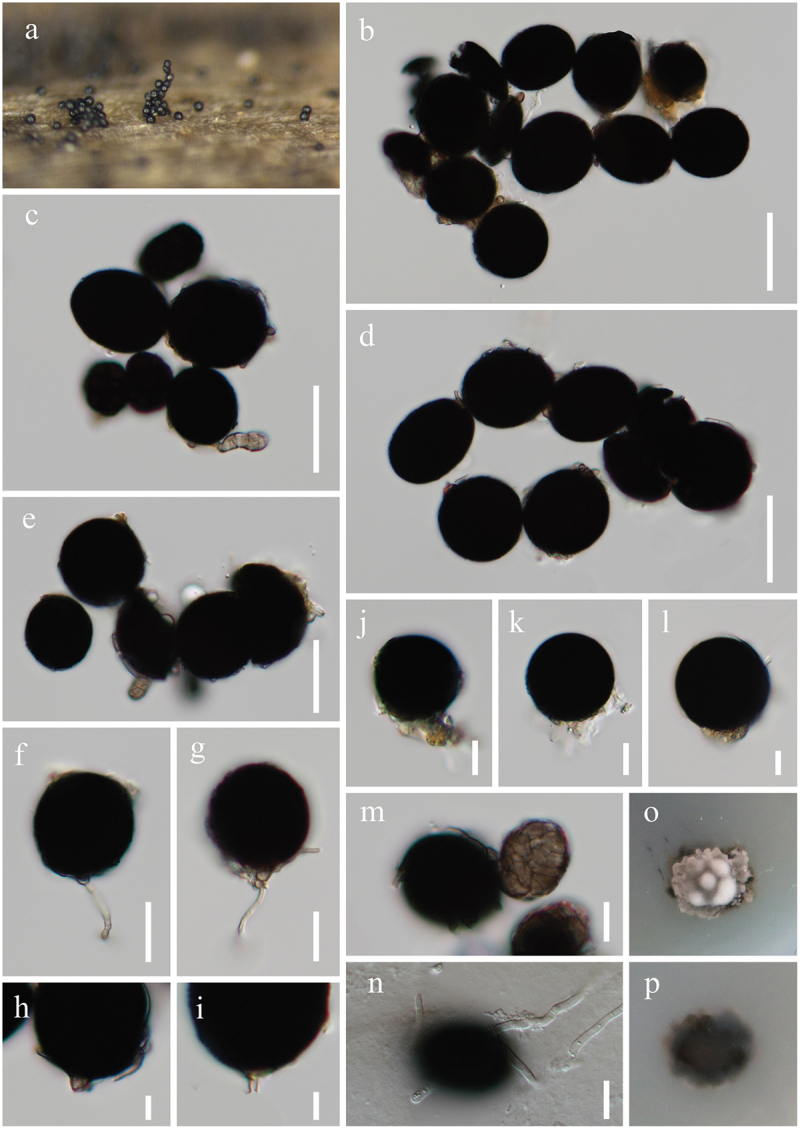
*Dematipyriforma aquatica* (HKAS 139441). (a) Colonies on the substratum. (b–e) Mount of conidia, conidiophores with conidia. (f–i) Hyphae with conidia. (j–m) Conidia. (n) Germinated conidium. (o, p) Colony on PDA from surface and reverse. Scale bars: b, d = 30 µm, c, e = 25 µm, f, g = 15 µm, h, i = 5 µm, j–n = 10 µm.

*Fungal Names number*: FN900081.

*Synonyms*: ***Dematipyriforma globispora*** Abdel-Aziz & Abdel-Wahab, Fungal Divers. 117: 134 (2022), syn. nov.

***Dematipyriforma americana*** Crous & Jurjević, Fungal Systematics and Evolution 13: 367 (2024), syn. nov.

*Saprobic* on submerged decaying wood. **Asexual morph**: *Colonies* effuse, superficial, globose conidia gathered or scattered, black, glistening. *Mycelium* mostly immersed, composed of septate, smooth-walled, unbranched, smooth, pale brown hyphae. *Conidiophores* reduced or micronematous, mononematous, pale brown to brown, subcylindrical, septate, straight or flexuous, 8.5–11 × 1.8–5.5 µm. *Conidiogenous cells* monoblastic, integrated, terminal, determinate, pale brown. *Conidia* 20–36 × 15–36 µm ($\overset{ˉ}{x}$ = 29.3 × 26.4 µm, *n* = 50), acrogenous, solitary, mostly directly produced from hyphae, thin-walled, ovoidal or subglobose to globose, muriform, with irregular, thin septate, not constricted at the septum, brown when young, black when mature. **Sexual morph**: Undetermined.

Culture characteristics: Conidia germinating on PDA within 24 h, producing germ tubes. Colonies on PDA reaching 20 mm in diameter after 4 weeks at room temperature. Mycelia dry, dense. Colonies semi-immersed in PDA, with irregular edges, slightly protrusion, dark green, then the surface were covered with a layer of white to grey hyphae, brown to dark green from below.

Material examined: China, Yunnan Province, Wenshan Autonomous Prefecture, Qiubei County, Xiaohuishan reservoir (24°16′34.33″N; 104°13′77.45″E), on unknown submerged decaying wood, 17 July 2023, Fa-Li Li, S-5835 (HKAS 139441), living cultures, CGMCC 3.27767 = KUNCC 23-16844.

Notes: Phylogenetic analysis showed that the new collection (KUNCC 23-16844) clustered with *Dematipyriforma americana*, *D. aquatica*, and *D. globispora* with 98% ML/1.00 PP support (Figure 1). *Dematipyriforma aquatica* and *D. globispora* were introduced by Jayawardena et al. (2022) on submerged wood in Egypt, while *D. americana* was described by Crous et al. (2024) as originating from a basement wall. Comparisons of the LSU sequence of the new collection with *D. americana*, *D. aquatica*, and *D. globispora* showed a similarity of 100% (809/809 bp), 99.88% (808/809 bp), and 99.88% (808/809 bp). The ITS sequence comparison of the new collection to *D. americana* reveals a similarity of 98.75% (555/562 bp, two gaps). There are no ITS sequences in GenBank for *D. aquatica* and *D. globispora*, and therefore they could not be analysed and compared. Although these species exhibit some slight morphological differences, phylogenetic analysis and DNA sequences cannot really delineate them. We therefore identify our new collection as *D. aquatica*, and synonymise *D. americana* and *D. globispora* with *D. aquatica*.

***Dematipyriforma aquilariae*** L.Y. Sun, H.Y. Li, X. Sun & L.D. Guo, Cryptogam. Mycol. 38(3): 345 (2017)

*Fungal Names number*: FN842402.

*Synonym*: ***Dematipyriforma nilotica*** Abdel-Aziz & Abdel-Wahab, Fungal Divers. 117: 135 (2022), syn. nov.

Notes: In the phylogenetic analysis, *Dematipyriforma nilotica* (SUMCC 12103) is nested within *D. aquilariae* with 100% ML/1.00 PP support (Figure 1). *Dematipyriforma nilotica* was introduced by Jayawardena et al. (2022), and it is phylogenetically related to *D. aquilariae*. Jayawardena et al. (2022) compared the LSU sequence of two species and reported that there are 15 base pairs differences. However, we re-compared the base pairs in the LSU sequence between the holotype of *D. nilotica* and *D. aquilariae* and we herein report 100% (590/590 bp) similarity. There is no ITS sequence of *D. nilotica* in GenBank, so comparison is not possible. Morphologically, *D. nilotica* has micronematous, mononematous, 2–5 µm wide conidiophores and pyriform, muriform conidia with transverse and longitudinal septa, which is similar to *D. aquilariae* (Sun et al. 2017). Therefore, we synonymise *D. nilotica* with *D. aquilariae* based on phylogenetic analysis and morphological resemblance. Currently, five species including *D. aquatica*, *D. aquilariae*, *D. muriformis*, *D. nigrospora*, and *D. terrestris* are accepted in *Dematipyriforma*.

***Phaeoisaria*** Höhn.

Notes: *Phaeoisaria* was introduced by Höhnel (1909), with *P. bambusae* as the type species. The asexual morph of *Phaeoisaria* species is influenced by substrate on which it grows. On woody substratum, it is characterised by synnemata composed of parallelly adpressed conidiophores, numerous sympodially extending denticulate conidiogenous cells and aseptate, obovoidal conidia (Cheng et al. 2014; Liu et al. 2015, 2022; Hyde et al. 2017; Luo et al. 2018a; Boonmee et al. 2021). In culture, it is characterised by reduced or macronematous, mononematous or synnematous conidiophores, aseptate or uniseptate conidia and dark chlamydospores (Crous et al. 2015, 2017; Réblová et al. 2016; Jayawardena et al. 2022; Wang et al. 2024a). The sexual morph of *Phaeoisaria* is only known for *P. filiformis* and is characterised by globose-to-elongate ascomata with a long neck, cylindrical asci, and filiform ascospores (Luo et al. 2019). In this study, we introduce a new species, *Phaeoisaria mononematosa*, and rediscover *P. filiformis* from Guangxi, China, but the new collection is different from the holotype of *P. filiformis* in morphology. Furthermore, the morphological descriptions of *P. annesophieae*, *P. filiformis*, *P. motuoensis*, and *P. obovata* in culture were provided.

***Phaeoisaria annesophieae*** Hern.-Restr., Persoonia 39: 447 (2017), Figure 7

**Figure 7.**
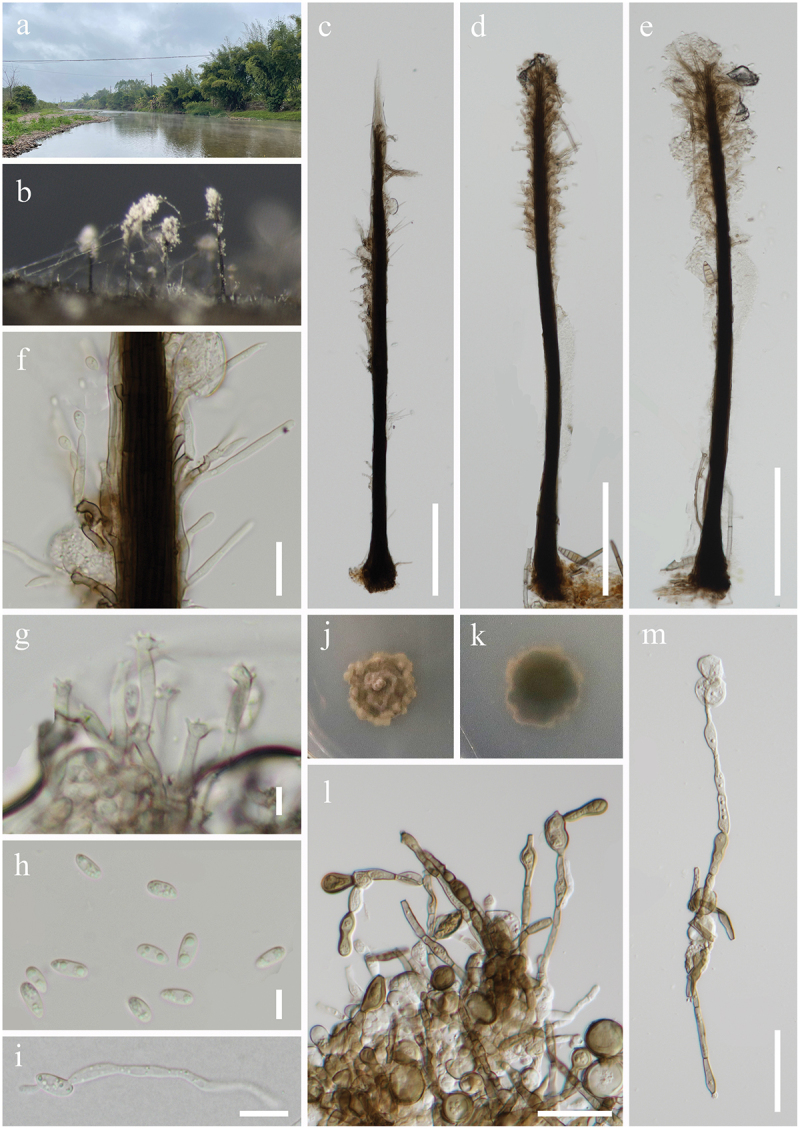
*Phaeoisaria annesophieae* (HKAS 139455). (a) Freshwater habitat. (b) Colonies on the substratum. (c–e) Synnemata. (f) Conidiogenous cells with conidia. (g) Conidiogenous cells. (h) Conidia. (i) Germinating conidium. (j, k) Colony on PDA from surface and reverse. (l, m) Chlamydospores. Scale bars: c–e = 100 µm, f = 15 µm, g, h = 5 µm, i = 10 µm, l = 25 µm, m = 30 µm.

*Fungal Names number*: FN823031.

*Saprobic* on submerged decaying wood. **Asexual morph**: *Colonies* erect, scattered, hairy, dark brown to black synnemata covered by white, velvety conidia. *Mycelium* partly immersed, partly superficial, composed of septate, smooth, branched, pale brown hyphae. *Conidiophores* macronematous, synnematous, cylindrical, septate, branched at the apex, straight, dark brown, smooth, thick-walled. *Synnemata* 221–598 × 8.2–16 µm ($\overset{ˉ}{x}$ = 452.5 × 12.9 µm, *n* = 10), rigid, dark brown to black, composed of compact appressed conidiophores. *Conidiogenous cells* 13–43 × 1.6–2.8 µm ($\overset{ˉ}{x}$ = 25.4 × 2.2 µm, *n* = 30), integrated, terminal, polyblastic, cylindrical to subcylindrical, straight to curved, percurrent proliferating, inflated apical with one to several small denticulate, subhyaline conidiogenous loci. *Conidia* 4.7–8.4 × 3.5 µm ($\overset{ˉ}{x}$ = 6.7 × 2.9 µm, *n* = 50), acrogenous, solitary, obovoidal, smooth-walled, rounded apical and taper at the base, hyaline, aseptate, straight, guttulate. **Sexual morph**: Undetermined.

Culture characteristics: Conidia germinating on PDA within 24 h, with germ tubes produced from both ends. Colonies on PDA reaching 20 mm in diameter after 4 weeks at room temperature. Mycelia dry, dense. Colonies semi-immersed in PDA, with irregular edges, surface rough, central protrusion, centre is grey, turning brownish-yellow with brown edges in colour, smooth, dark green with brown edges from below. *Chlamydospores* 7.6–18 × 4.5–13 µm ($\overset{ˉ}{x}$ = 11.9 × 8.8 µm, *n* = 30), produced from mycelia, intercalary, lateral to terminal, solitary or catenate, obovoidal to pyriform or globose to subglobose, thick, smooth-walled, rounded at the apex, guttulate, aseptate, hyaline when young, brown when mature. *Secession schizolytic*.

Material examined: China, Guangxi Autonomous Region, Liuzhou (22°37′76.62″N; 110°05′19.30″E), on unknown submerged decaying wood in a freshwater stream, 25 February 2024, Tian-Tian Zhao, S-6229 (HKAS 139455), living culture, KUNCC 24-18183.

Notes: In our phylogenetic tree, our new collection of *Phaeoisaria annesophieae* (KUNCC 24-18183) clustered with two strains of *P. annesophieae* (CBS 143235 and MFLUCC 19-0325) with 100% ML/1.00 PP support. Our new collection is similar to *P. annesophieae* in having cylindrical, conspicuous denticles clustered in the apical region, obovoidal, aseptate, guttulate conidia with rounded apical, and globose or pyriform chlamydospores (Crous et al. 2017; Shi et al. 2021). We therefore identify our new collection as *P. annesophieae*, a species introduced by Crous et al. (2017) isolated from soil in Netherlands. Shi et al. (2021) also described *P. annesophieae* from freshwater habitat in Thailand, and it is reported for the first time in China.

***Phaeoisaria ellipsoidea*** R. Zhu & H. Zhang, Phytotaxa 591 (1): 25 (2023), Figure 8

**Figure 8.**
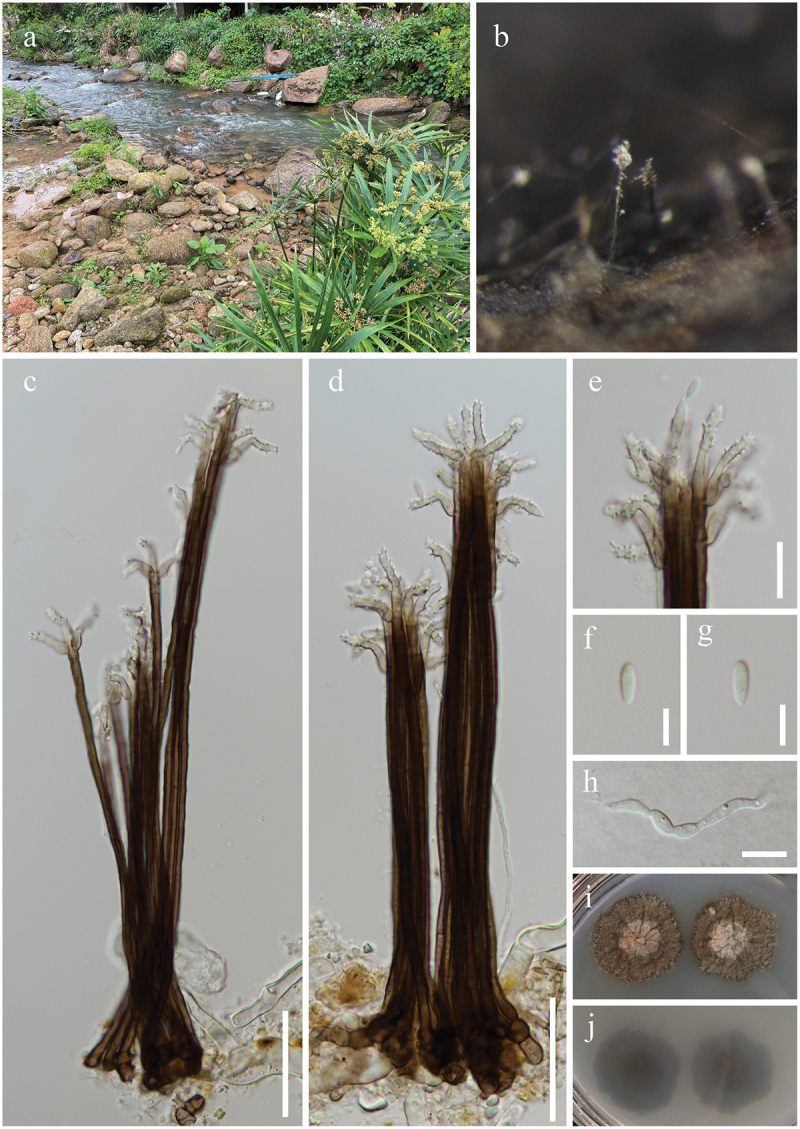
*Phaeoisaria ellipsoidea* (HKAS 139468). (a) Freshwater habitat. (b) Colonies on the substratum. (c, d) Synnemata. (e) Apex of synnema. (f, g) Conidia. (h) Germinating conidium. (i, j) Colonies on PDA from surface and reverse. Scale bars: c, d = 30 µm, e, h = 10 µm, f, g = 5 µm.

*Fungal Names number*: FN900262.

*Saprobic* on submerged decaying wood. **Asexual morph**: *Colonies* erect, scattered, hairy, dark brown to black synnemata covered by white, velvety conidia. *Mycelium* partly immersed, partly superficial, composed of septate, smooth, unbranched, hyaline to pale brown hyphae. *Conidiophores* 43–139 (−201) × 1.7–2.3 µm ($\overset{ˉ}{x}$ = 101.9 × 2 µm, *n* = 10), macronematous, synnematous, cylindrical, branched at the apex, brown, septate, smooth, thick-walled. *Synnemata* 101–235 × 4.6–14 µm ($\overset{ˉ}{x}$ = 168.1 × 8.3 µm, *n* = 10), rigid, cylindrical, brown to dark brown, with subhyaline to pale brown, flared conidiogenous cells in the above half, composed of compact and incompact conidiophores. *Conidiogenous cells* 6.5–18 × 1.5–2.8 µm ($\overset{ˉ}{x}$ = 12.4 × 2.1 µm, *n* = 30), polyblastic, terminal, integrated, curved, hyaline to pale brown, sympodial, with multiple denticulate tiny conidiogenous loci. *Conidia* 4–5.8 × 1.5–2.1 µm ($\overset{ˉ}{x}$ = 5.2 × 1.8 µm, *n* = 20), acrogenous, solitary, elongated obovoidal to fusiform, straight, rounded at both ends, aseptate, hyaline, smooth-walled. **Sexual morph**: Undetermined.

Culture characteristics: Conidia germinating on PDA within 12 h, with germ tubes produced from both ends. Colonies on PDA reaching 20 mm in diameter after 5 weeks at room temperature. Mycelia dry, dense. Colonies on the surface of PDA, with regular edges, surface rough with pucker, centre is white, turning brown, brown to dark brown, smooth from below.

Material examined: China, Guangxi Autonomous Region, Yulin (22°24′48.70″N; 109°70′42.37″E), on unknown submerged decaying wood in a freshwater stream, 25 February 2024, Wen-Peng Wang, S-6460 (HKAS 139468), living culture, KUNCC 24-18390.

Notes: Phylogenetic analysis showed that our new collection clustered with the ex-type strain of *Phaeoisaria ellipsoidea* with 100% ML/1.00 PP support (Figure 1). Comparisons of the LSU and ITS sequences of our new collection and the holotype of *P. ellipsoidea* reveal 100% (810/810 bp) and 99.2% (495/499 bp, three gaps) similarity, respectively. However, our new collection has branched, significantly shorter conidiophores [43–139 (−201) µm vs. up to 1,200 µm], and smaller conidia (4–5.8 × 1.5–2.1 µm vs. 4–8 × 3–4.5 µm), which are the only morphological differences from the holotype of *P. ellipsoidea* (Yang et al. 2023b). Therefore, we identify our new collection as *Phaeoisaria ellipsoidea* based on phylogenetic analysis and sequence data.

***Phaeoisaria filiformis*** D.F. Bao, Z.L. Luo, K.D. Hyde & H.Y. Su, Fungal Divers. 99: 565 (2019), Figures 9, 10

**Figure 9.**
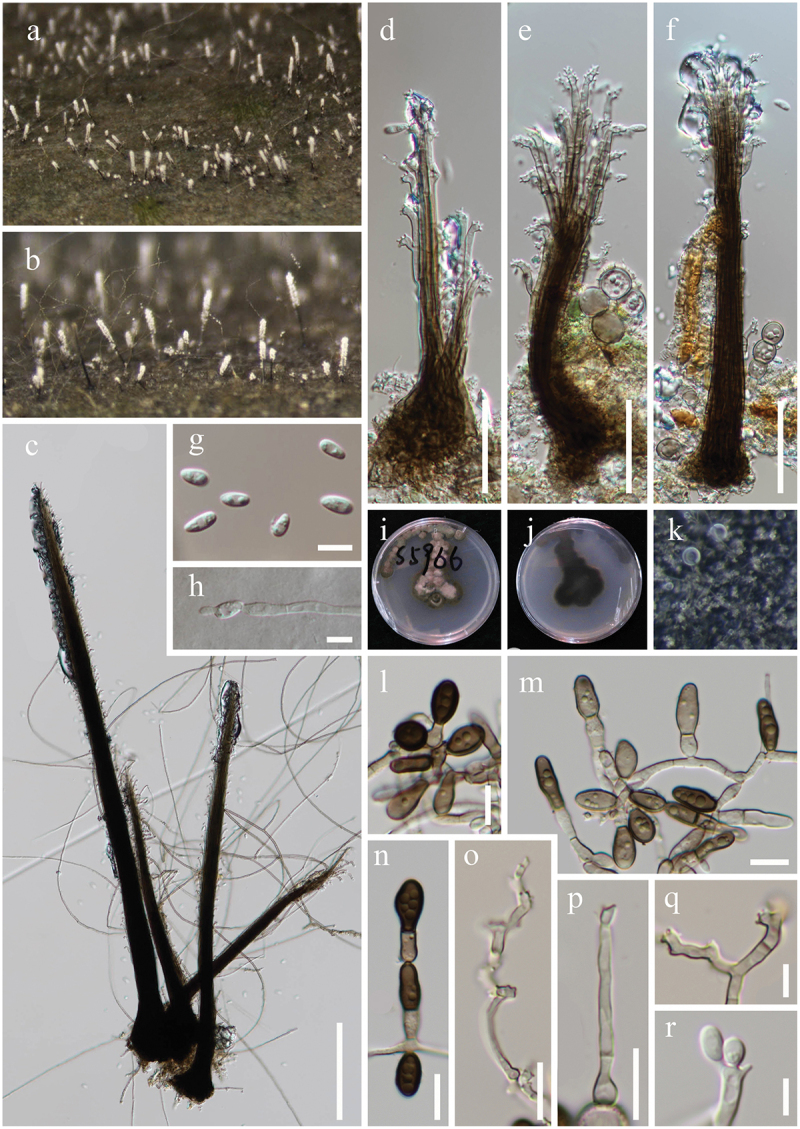
Asexual morph of *Phaeoisaria filiformis* (a–j, HKAS 139466; k–r, HKAS 139438). (a, b) Colonies on the substratum. (c–f) Synnemata. (g) Conidia. (h) Germinating conidium. (i, j) Colony on PDA from surface and reverse. (k–r) Sporulation observed on PDA [(k) Colonies on PDA; (l–n) Chlamydospores; (o–q) Conidiogenous cells; (r) Conidiogenous cell with conidia]. Scale bars: c = 100 µm, d–f = 25 µm, g, h, q, r = 5 µm, l–p = 10 µm.

**Figure 10.**
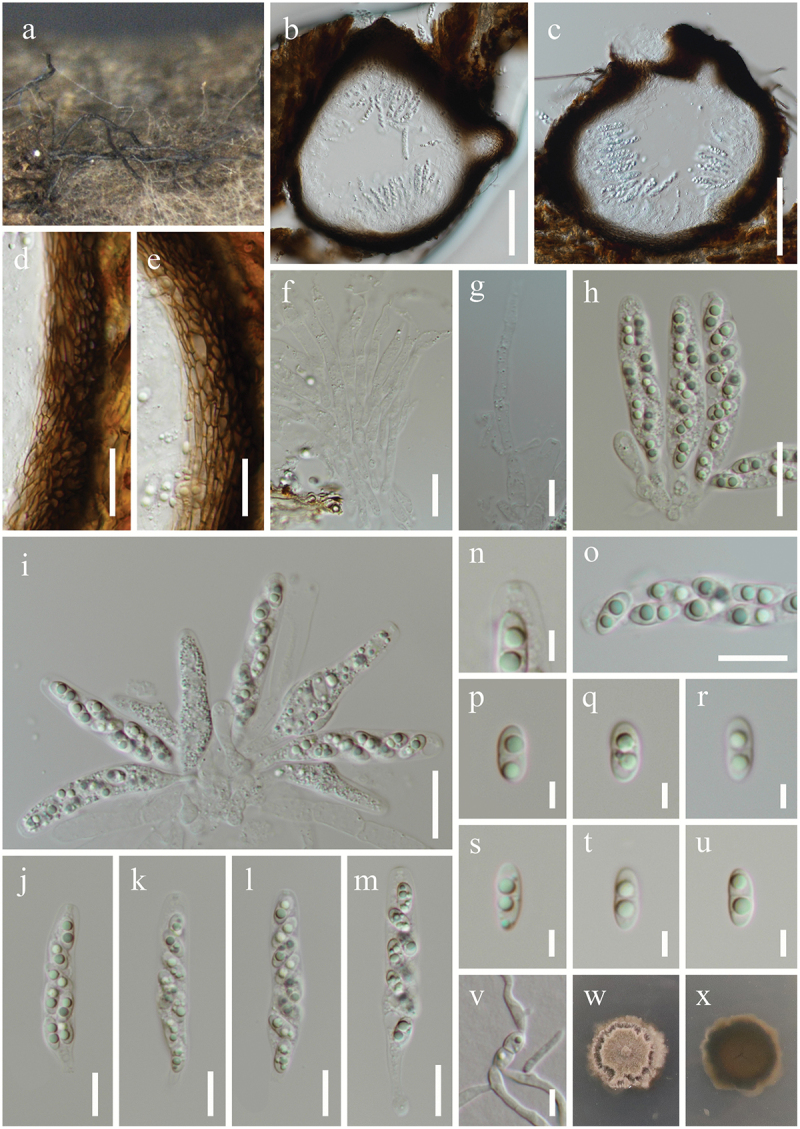
Sexual morph of *Phaeoisaria filiformis* (HKAS 139438). (a) Ascomata on the substratum. (b, c) Vertical section of ascomata. (d, e) Structure of peridium. (f, g) Paraphyses. (h–m) Asci. (n) Apex of ascus. (o–u) Ascospores. (v) Germinating ascospore. (w, x) Colony on PDA from surface and reverse. Scale bars: b, c = 70 µm, d, e, h, i = 15 µm, f, g, j–m, o = 10 µm, n, p–u = 3 µm, v = 5 µm.

*Fungal Names number*: FN555671.

*Saprobic* on submerged decaying wood. **Asexual morph**: *Colonies* erect, hairy, scattered or gathered in groups, dark brown to black synnemata covered by white, velvety conidia. *Mycelium* mostly superficial, composed of branched, septate, pale brown to brown, smooth hyphae. *Conidiophores* macronematous, synnematous, cylindrical, brown, paler towards the apex, hyaline at the apex, septate, smooth, thick-walled. *Synnemata* 94–305 × 8.2–25 µm ($\overset{ˉ}{x}$ = 254 × 12.7 µm, *n* = 20), rigid or slightly flexuous, brown to dark brown, paler towards the apex, hyaline to pale brown at the apex, composed of compactly conidiophores, with flared conidiogenous cells in the above half to the apex. *Conidiogenous cells* 3.2–15.5 × 1.6–2.4 µm ($\overset{ˉ}{x}$ = 7.9 × 2 µm, *n* = 30), polyblastic, terminal, integrated, curved, hyaline to brown, with multiple denticulate conidiogenous loci at the apex. *Conidia* 4.4–7.6 × 2–3.1 µm ($\overset{ˉ}{x}$ = 6.2 × 2.5 µm, *n* = 50), acrogenous, solitary, obovoidal, straight, rounded apical and obtuse base, aseptate, hyaline, smooth-walled, guttulate. **Sexual morph**: *Ascomata* 171–253 µm high, 190–232 µm diam., gathered, immersed, obovoidal to subglobose, sometimes a mammiform protrusion forms on the thicker side of the peridium inner, black, ostiolate, with a long, subcylindrical, flexuous neck. *Peridium* 9.3–32 µm thick, composed of multiple layers, thick-walled, brown, irregular polygons cells of *textura angularis*. *Paraphyses* 2.4–6.9 µm wide, unbranched, hyaline, tapering towards the apex, septate, slightly constricted at the septum. *Asci* 38–57 × 6.3–8.9 µm ($\overset{ˉ}{x}$ = 46.1 × 7.4 µm, *n* = 20), 8-spored, unitunicate, subcylindrical, slightly flexuous, slightly truncated at the apex, with 4.6–14.6 µm long pedicellate and a small apical ring. *Ascospores* 6.7–8.8 × 2.7–3.6 µm (Accessisdenied = 7.6 × 3.2 µm, *n* = 30), overlapping, uniseriate or vertical biseriate, fusoid or ellipsoidal, rounded at both ends, straight, uniseptate, slightly constricted at the septum, guttulate, hyaline, thin, smooth-walled, without gelatinous sheath or appendage.

Culture characteristics: Conidia germinating on PDA within 24 h, with germ tubes produced from both ends. Colonies on PDA reaching 20 mm in diameter after 4 weeks at room temperature. Mycelia dry, dense. Colonies on the surface of PDA, with regular edges, surface rough, slight protrusion, dark green, covered with a layer white hypha, dark green, smooth from below. Ascospores germinating on PDA within 24 h, with germ tubes produced from both ends. Colonies on PDA reaching 20 mm in diameter after 4 weeks at room temperature. Mycelia dry, dense. Colonies on the surface of PDA, with irregular edges, slightly protrusion, surface flat with a small protrusion, brownish-yellow, sectored from the centre, dark brown to dark green with brown edges from below. *Conidiophores* reduced. *Conidiogenous cells* 10.7–31 × 1.6–3.1 µm ($\overset{ˉ}{x}$ = 18.8 × 2.1 µm, *n* = 20), polyblastic, terminal, integrated, cylindrical, curved to recurved, hyaline to pale brown, smooth-walled, percurrent proliferating, with several denticulate conidiogenous loci at the apex. *Conidia* 3.4–7.4 × (1.6–) 2.1–4.5 µm ($\overset{ˉ}{x}$ = 4.9 × 2.7 µm, *n* = 50), acrogenous, solitary, obovoidal to subglobose, straight, rounded at both ends, aseptate, hyaline, smooth-walled. *Chlamydospores* 7.6–15 × 5.2–9.6 µm ($\overset{ˉ}{x}$ = 11 × 7.2 µm, *n* = 50), produced from a cylindrical or subglobose, hyaline to brown cell, intercalary, lateral to terminal, solitary, obovoidal to pyriform or globose to subglobose, smooth-walled, rounded at the apex, guttulate, aseptate, hyaline when young, brown to dark brown when mature. *Secession schizolytic*.

Material examined: China, Yunnan Province, Wenshan Autonomous Prefecture, Guangnan County (24°29′33.74′′N; 105°07′02.35′′E), on unknown submerged decaying wood in a freshwater stream, 7 February 2022, Wen-Peng Wang, S-3511 (HKAS 131388), living culture, KUNCC 23-13723; Guangxi Autonomous Region, Nanning (22°48′48″N; 108°13′32″E), on unknown submerged decaying wood in a freshwater river, 17 November 2023, Qiu-Xia Yang, S-5966 (HKAS 139466), living culture, KUNCC 23-17567; Baise (24°08′90.39″N; 106°64′83.78″E), on unknown submerged decaying wood in a freshwater stream, 18 February 2024, Tian-Tian Zhao, S-6073 (HKAS 139443), living cultures, CGMCC 3.27770 = KUNCC 24-17755; Qinzhou (22°14′61.07″N; 108°61′73.78″E), on unknown submerged decaying wood in a freshwater stream, 26 February 2024, Wen-Peng Wang, S-6348 (HKAS 139438), living culture, KUNCC 24-18262.

Notes: Phylogenetic analysis showed that our new collections clustered with the type strain of *Phaeoisaria filiformis* (Figure 1). Comparisons of the LSU and ITS sequences of the new collection (HKAS 139438) and the holotype of *P. filiformis* reveal a 99.87% (773/774 bp) and 99.81% (524/525 bp) similarity, respectively. Therefore, we identify our new collections as *P. filiformis*, a species introduced by Luo et al. (2019), which was the first sexual species in *Phaeoisaria*. However, the holotype of *P. filiformis* has cylindrical asci without pedicellate, filiform, tapering at both ends, multiseptate ascospores; the new collection (HKAS 139438) has subcylindrical, pedicellate, smaller (38–57 × 6.3–8.9 µm vs. 120–136 × 12–14 µm) asci slightly truncated at the apex, and fusoid or ellipsoidal, rounded at both ends, straight, uniseptate conidia, which is different from the holotype (Luo et al. 2019). We identify our collections as *P. filiformis* but more sexual *Phaeoisaria* species need to be collected and analysed to explain why the two specimens have different morphologies.

***Phaeoisaria guttulata*** J. Yang & K.D. Hyde, Mycosphere 9 (2): 401 (2018), Figure 11

**Figure 11.**
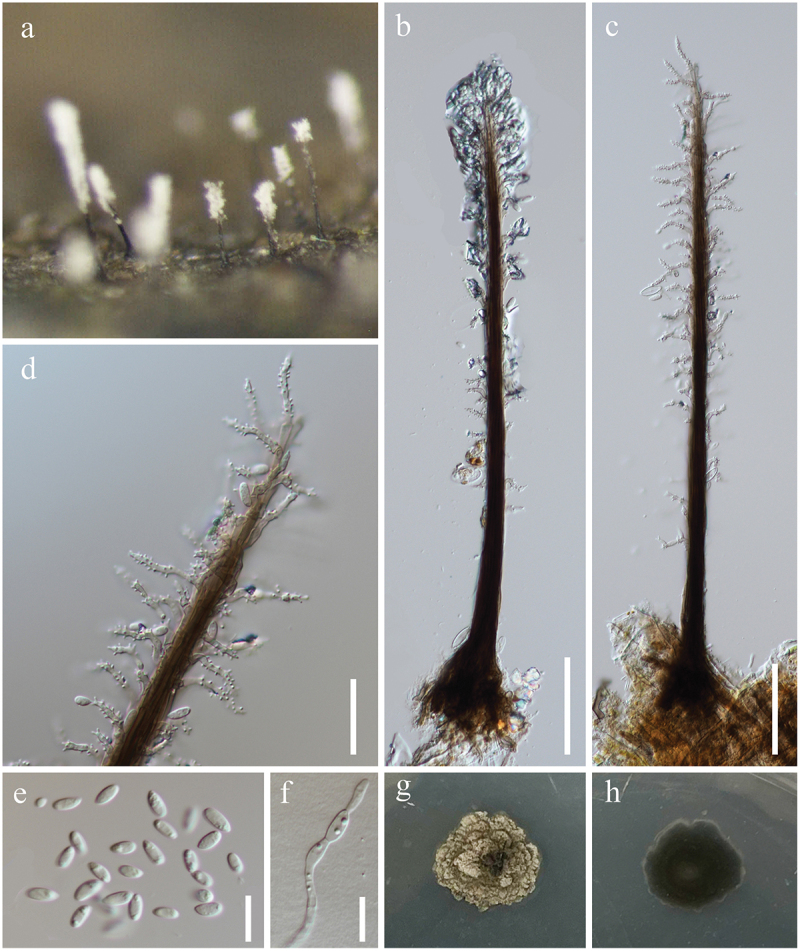
*Phaeoisaria guttulata* (HKAS 139428). (a) Colonies on the substratum. (b, c) Synnemata. (d) Apex of synnema. (e) Conidia. (f) Germinating conidium. (g, h) Colony on PDA from surface and reverse. Scale bars: b, c = 50 µm, d = 20 µm, e, f = 10 µm.

*Fungal Names number*: FN554233.

Material examined: China, Yunnan Province, Qujing, Luoping County (25°01′27.11″N; 104°36′75.96″E), on unknown submerged decaying wood in a freshwater river, 15 July 2023, Xing-Ya Zeng, S-5438 (HKAS 139428), living culture, KUNCC 23-15646.

***Phaeoisaria laianensis*** Y. Liu, G.P. Xu, X.Y. Yan, D.M. Hu & Z.J. Zhai, Biodivers. Data J. 10(e94088): 6 (2022)

*Fungal Names number*: FN844773.

Material examined: China, Guangxi Autonomous Region, Guilin (24°60′46.51″N; 110°42′06.30″E), on unknown submerged decaying wood in a freshwater stream, 21 February 2024, Zheng-Quan Zhang, S-6049 (HKAS 139442), living culture, KUNCC 24-17745.

***Phaeoisaria loranthacearum*** Crous & R.K. Schumach., Sydowia 67: 110 (2015), Figure 12

**Figure 12.**
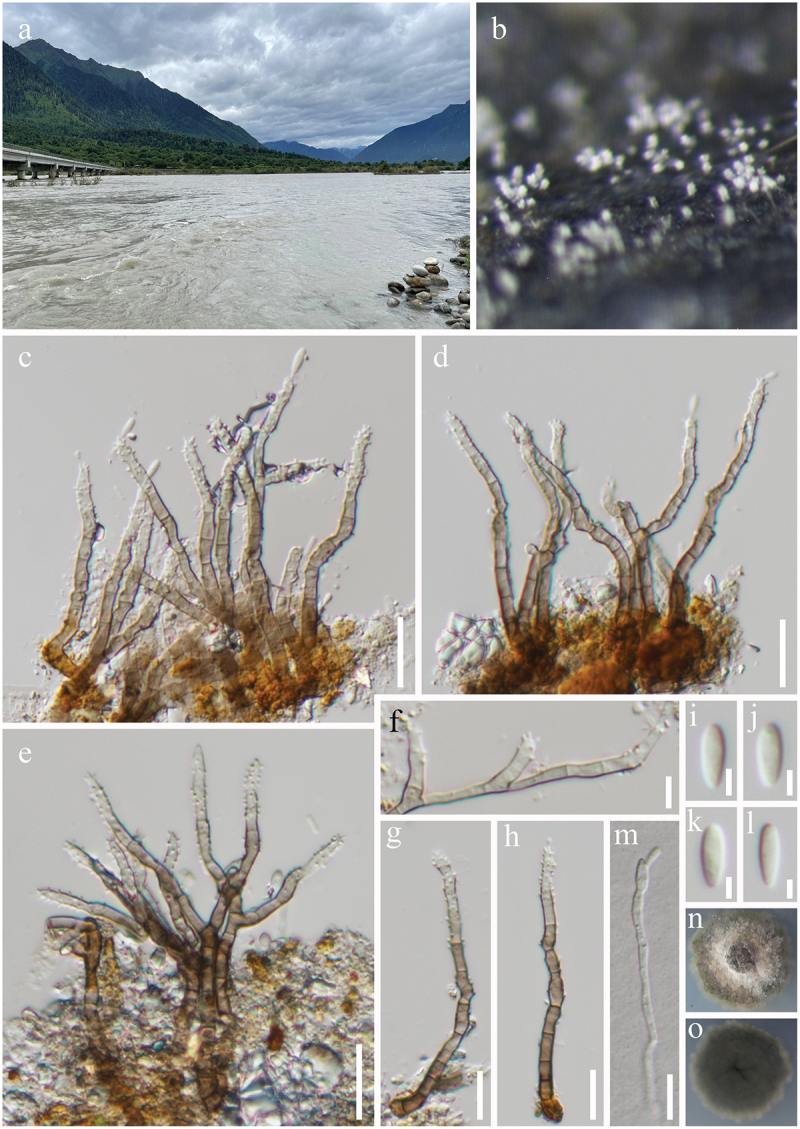
*Phaeoisaria loranthacearum* (HKAS 146439). (a) Freshwater habitat. (b) Colonies on the substratum. (c–h) Conidiophores. (i–l) Conidia. (m) Germinating conidium. (n, o) Colony on PDA from surface and reverse. Scale bars: c–e = 15 µm, f = 5 µm, g, h, m = 10 µm, i–l = 2 µm.

*Fungal Names number*: FN812551.

*Synonyms*: ***Phaeoisaria fasciculata*** Réblová & Seifert, Persoonia 37: 69 (2016), syn. nov.

*Saprobic* on submerged decaying wood. **Asexual morph**: *Colonies* erect, scattered, hairy, white conidia gathered at the apex of conidiophores. *Mycelium* mostly immersed, composed of aseptate, branched, smooth, hyaline hyphae. *Conidiophores* 25–69 × 2– 3.2 µm ($\overset{ˉ}{x}$ = 51.2 × 2.6 µm, *n* = 30), macronematous to semi-macronematous, mononematous, solitary or in small groups, cylindrical, straight to slightly flexuous, unbranched or branched at the middle, septate, brown, paler towards the apex, hyaline to pale brown at the apex, thick-walled, partly reduced to conidiogenous cells. *Conidiogenous cells* integrated, terminal to intercalary, polyblastic, cylindrical, sympodial, hyaline to pale brown, with multiple cylindrical, denticulate conidiogenous loci. *Conidia* 4.9–7.1 × 1.6–2.7 µm ($\overset{ˉ}{x}$ = 6.1 × 2 µm, *n* = 30), acropleurogenous, solitary, ellipsoidal to ovate, straight, aseptate, hyaline, obtuse to rounded at both ends. **Sexual morph**: Undetermined.

Culture characteristics: Conidia germinating on PDA within 12 h, and germ tubes produced from both ends. Colonies on PDA reaching 20 mm in diameter after 4 weeks at room temperature. Mycelia dry, dense. Colonies semi-immersed in PDA, compaction, umbellate, with irregular edges, dark brown to white, with blue-grey edges, surface rough, split from the centre, dark brown with pale edges below.

Material examined: China, Xizang Autonomous Region, Linzhi, Bomi County (29°45′18.26″N; 95°58′37.64″E), on unknown submerged decaying wood in Palongzangbu River, 26 July 2024, Wen-Peng Wang, S-6694 (HKAS 146439), living culture, CGMCC 3.28756 = KUNCC 24-18588.

Notes: Prior to this discovery, all known species of *Phaeoisaria* found on decaying wood produced synematous conidiophores, whereas this specimen (HKAS 146439) produces mononematous conidiophores on the wood substrate. Phylogenetic analysis showed that our new collection, *Phaeoisaria fasciculata*, and *P. loranthacearum* clustered with a clade of *Phaeoisaria* with 99% ML/1.00 PP support (Figure 1). Morphologically, our new collection, *P. fasciculata*, and *P. loranthacearum* share mononematous, macronematous to semi-macronematous or reduced conidiophores, without typical indeterminate synnemata of this genus, and micro conidia (Crous et al. 2015; Réblová et al. 2016). Comparisons of the ITS and LSU sequences of the ex-type strain of *P. fasciculata* and *P. loranthacearum* showed 0.2% (1/501, three gaps) and 1.39% (12/863, four gaps) difference, respectively. We therefore synonymise *P. fasciculata* with *P. loranthacearum* based on phylogenetic analysis and morphological characteristics, and identify our new collection as *P. loranthacearum*.

***Phaeoisaria microspora*** C.G. Lin & K.D. Hyde, Fungal Divers. 87: 160 (2017), Figure 13

**Figure 13.**
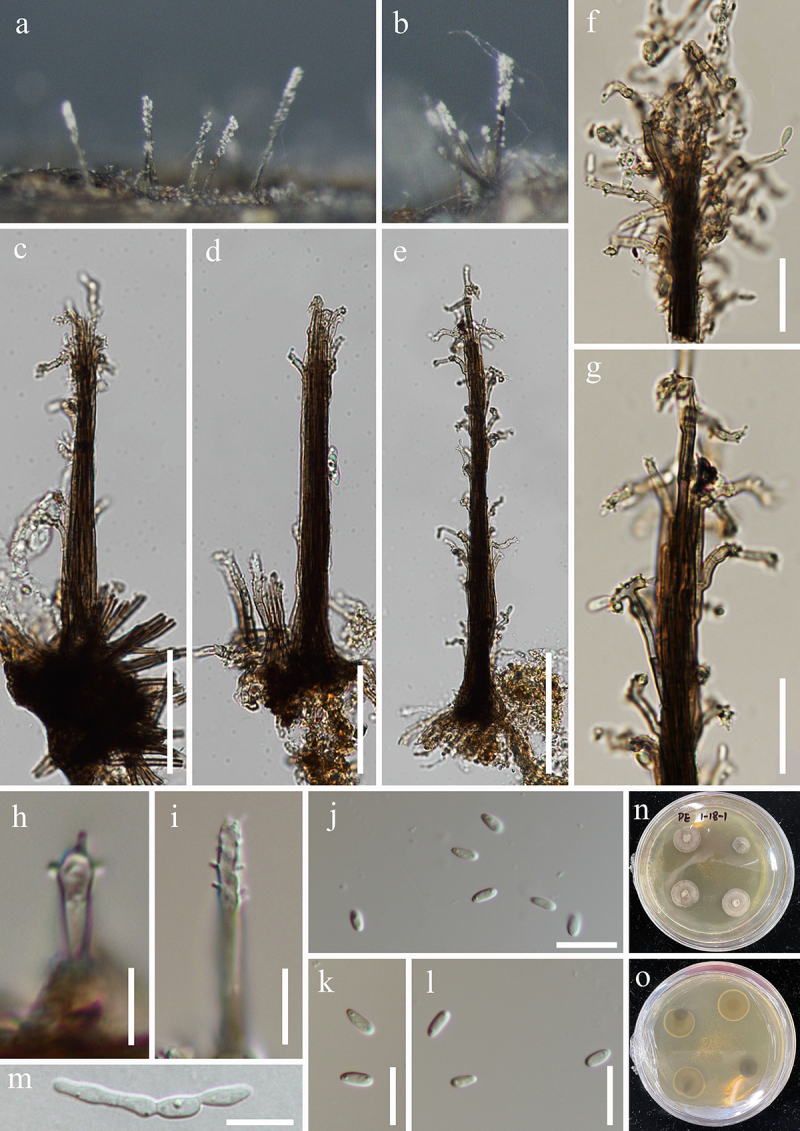
*Phaeoisaria microspora* (HKAS 131968). (a, b) Colonies on the substratum. (c–e) Synnemata. (f, g) Apex of synnemata. (h, i) Conidiogenous cells. (j–l) Conidia. (m) Germinating conidium. (n, o) Colonies on PDA from surface and reverse. Scale bars: c, d = 40 µm, e = 60 µm, f, g = 20 µm, h = 5 µm, i–m = 10 µm.

*Fungal Names number*: FN553261.

*Saprobic* on submerged decaying wood. **Asexual morph**: *Colonies* erect, solitary or gathered in groups, hairy, dark brown to black synnemata covered by white, velvety conidia. *Mycelium* mostly immersed, composed of septate, branched, brown hyphae. *Conidiophores* macronematous, synematous, septate, cylindrical, branched at the apex, straight, brown to dark brown, smooth, thick-walled. *Synnemata* 114.2–416.3 × 9.6–22.9 µm ($\overset{ˉ}{x}$ = 230.6 × 14.9 µm, *n* = 10), rigid, cylindrical to subulate, brown to dark brown, composed of compact appressed conidiophores, with flared conidiogenous cells cover up to the base, sometimes with multiple short, mononematous conidiophores gathered at the base. *Conidiogenous cells* 7.2–30.5 × 1.3–2.5 µm ($\overset{ˉ}{x}$ = 15.9 × 2 µm, *n* = 20), integrated, terminal, polyblastic, cylindrical, sympodial, straight to recurved, with multiple cylindrical, denticulate conidiogenous loci, hyaline to pale brown. *Conidia* 4.4–7.4 × 1.6–2.8 µm ($\overset{ˉ}{x}$ = 5.8 × 2.3 µm, *n* = 40), acrogenous, solitary, clavate to obovoidal, straight or slightly curved, aseptate, hyaline, smooth-walled, guttulate, obtuse at both ends. **Sexual morph**: Undetermined.

Culture characteristics: Conidia germinating on PDA within 12 h, and germ tubes produced from both ends. Colonies on PDA reaching 20 mm in diameter after 4 weeks at room temperature. Mycelia dry, dense. Colonies on the surface of the PDA, compaction, globose protrusion in the centre, with regular edges, grey to brown, circular, brown to brownish-yellow to dark brown, with lighter edges, smooth from below.

Material examined: China, Yunnan Province, Pu’er (22°71′62.42″N; 100°98′94.30″E), on unknown submerged decaying wood in a freshwater stream, 10 July 2022, Long-Li Li, S-3985 (HKAS 131968), living cultures, CGMCC 3.27763 = KUNCC 23-15498.

Notes: In the phylogenetic tree, our new collection (KUNCC 23-15498) is sister to the ex-type strain of *Phaeoisaria microspora* (MFLUCC 16-0033) with 100% ML/1.00 PP support (Figure 1). Morphologically, our new collection fits well with the description of *P. microspora* in having short synnemata, branched conidiophores, terminal, sympodial conidiogenous cells with multiple cylindrical denticulate conidiogenous loci, and aseptate conidia with similar size (4.4–7.4 × 1.6–2.8 µm vs. 4.5–6.9 × 1.3–3.1 µm) (Hyde et al. 2017). We therefore identify our new collection as *Phaeoisaria microspora* based on phylogenetic analysis and morphological characteristics.

**Figure 14.**
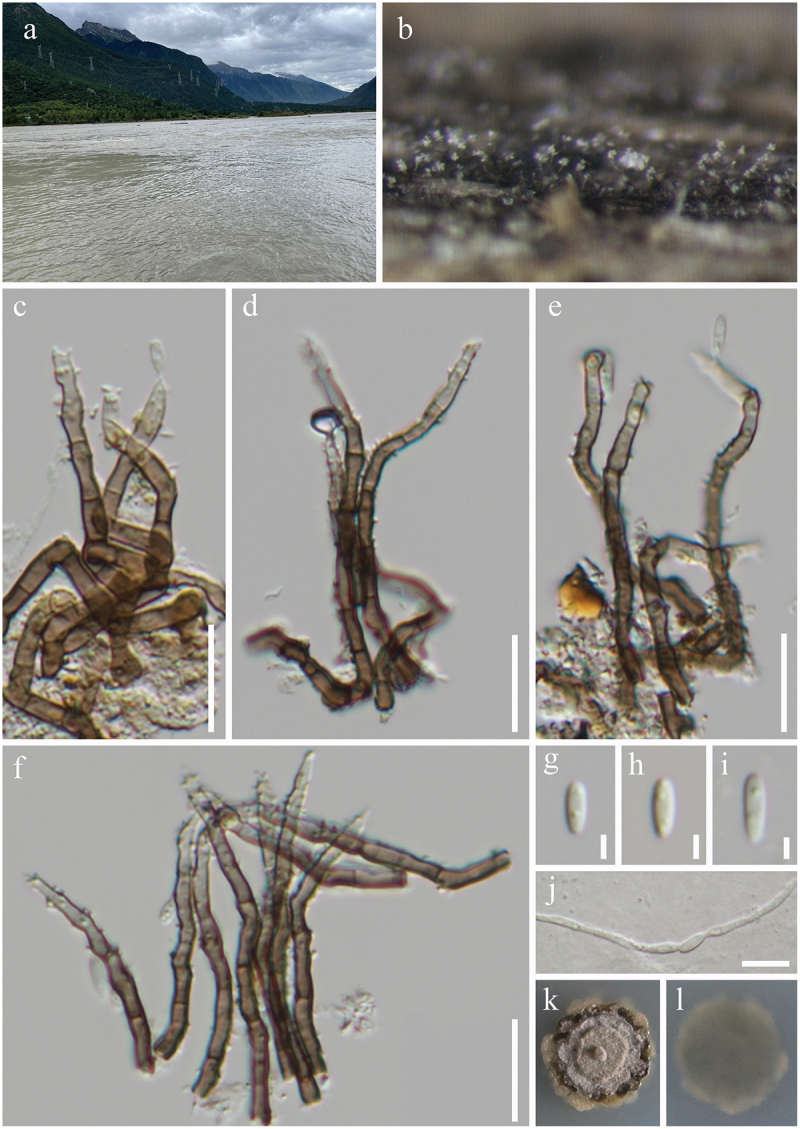
*Phaeoisaria mononematosa* (HKAS 146443, holotype). (a) Freshwater habitat. (b) Colonies on the substratum. (c–f) Conidiophores. (g–i) Conidia. (j) Germinating conidium. (k, l) Colony on PDA from surface and reverse. Scale bars: c–f = 15 µm, g–i = 2 µm, j = 10 µm.

***Phaeoisaria mononematosa*** W.P. Wang & Z.L. Luo, sp. nov., Figure 14

*Fungal Names number*: FN572470.

*Etymology*: Referring to the mononematous conidiophores.

*Holotype*: HKAS 146443.

*Saprobic* on submerged decaying wood. **Asexual morph**: *Colonies* erect, scattered, hairy, pale white conidia gathered at the apex of conidiophores. *Mycelium* mostly immersed, composed of aseptate, branched, smooth, hyaline hyphae. *Conidiophores* 29–81 × 1.4–2.7 µm ($\overset{ˉ}{x}$ = 48.1 × 2 µm, *n* = 20), macronematous, mononematous, solitary or in small groups, cylindrical, slightly flexuous to curved, unbranched, septate, brown, paler towards the apex, hyaline to pale brown at the apex, thick-walled. *Conidiogenous cells* integrated, terminal, polyblastic, cylindrical, sympodial, hyaline to pale brown, with multiple small, cylindrical, denticulate conidiogenous loci. *Conidia* 4.4–7.1 × 1.6–2.2 µm ($\overset{ˉ}{x}$ = 5.7 × 1.9 µm, *n* = 20), acrogenous, solitary, fusiform to ellipsoidal, straight, aseptate, hyaline, smooth-walled, guttulate, obtuse at both ends. **Sexual morph**: Undetermined.

Culture characteristics: Conidia germinating on PDA within 12 h, and germ tubes produced from both ends. Colonies on PDA reaching 20 mm in diameter after 4 weeks at room temperature. Mycelia dry, dense. Colonies semi-immersed in PDA, compaction, layer by layer from the outside to the inside, small globose in the centre, grey to brown, with irregular edges, surface rough, brown to grey from below.

Material examined: China, Xizang Autonomous Region, Linzhi, Bomi County (29°45′18.26″N; 95°58′37.64″E), on unknown submerged decaying wood in Palongzangbu River, 26 July 2024, Zong-Long Luo, S-6697 (HKAS 146443, holotype), ex-type culture, CGMCC 3.28757 = KUNCC 24-18591.

Notes: The phylogenetic tree showed that our new collection (KUNCC 24-18591) clustered with *Phaeoisaria sparsa* (FMR 11939) with 100% ML/1.00 PP support (Figure 1). Comparisons of the LSU and ITS sequences of the new collection (KUNCC 24-18591) and *P. sparsa* (FMR 11939) revealed a 1.14% (6/526 bp) and 1.20% (6/499 bp) difference, respectively. However, this strain (FMR 11939) is not derived from the holotype, and no morphological description is provided for this strain. Therefore, we compare the morphological characteristics of our new collection with the holotype of *P. sparsa*. *Phaeoisaria sparsa* have synematous, branched conidiophores and 0–3-septate conidia, whereas our new collection has mononematous, unbranched conidiophores and aseptate, smaller conidia (4.4–7.1 × 1.6–2.2 µm vs. 10–15.5 × 2.5–3.5 µm) (Sutton 1973). We therefore recognise our new collection as a new species mainly based on morphological characteristics.

***Phaeoisaria motuoensis*** K. Xu, R.J. Xu & Y.A. Zhu, Phytotaxa 642 (1): 67 (2024), Figure 15

**Figure 15.**
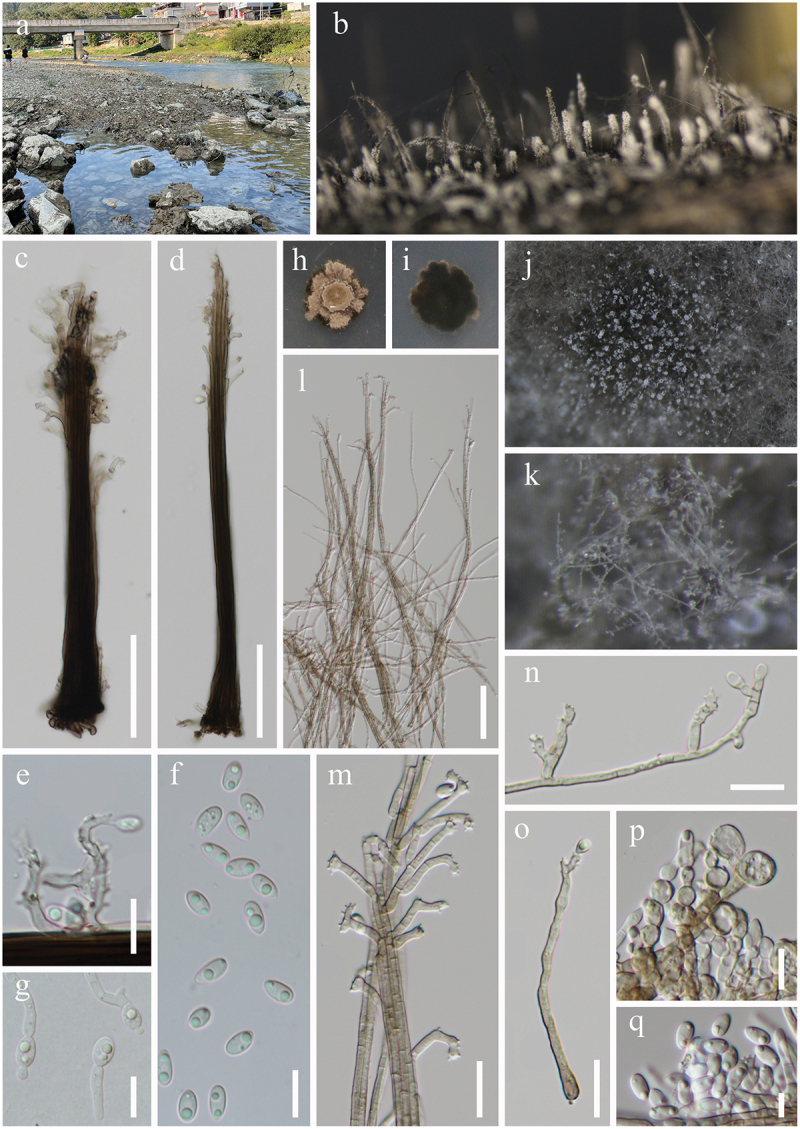
*Phaeoisaria motuoensis* (HKAS 139433). (a) Freshwater habitat. (b–f) Observed from substratum [(b) Colonies on the substratum; (c, d) Synnemata; (e) Conidiogenous cells; (f) Conidia]. (g) Germinating conidia. (h, i) Colony on PDA from surface and reverse. (j–q) Sporulation observed on PDA [(j, k) Colonies on PDA; (l) Synnemata; (m) Apex of synnema; (n) Conidiogenous cells; (o) Conidiophores with conidia; (p) Chlamydospores; (q) Conidia]. Scale bars: c, d, l = 50 µm, e–g, n, p = 10 µm, m, o = 15 µm, q = 5 µm.

*Fungal Names number*: FN900235.

*Saprobic* on submerged decaying wood. **Asexual morph**: *Colonies* erect, hairy, scattered or gathered, dark brown to black synnemata covered by white, velvety conidia. *Mycelium* partly immersed, partly superficial, composed of septate, smooth, branched, hyaline to pale brown hyphae. *Conidiophores* macronematous, synnematous, cylindrical, septate, unbranched, straight, brown to dark brown, paler towards the apex, smooth, thick-walled. *Synnemata* 212–1,364 × 5.5–43 µm ($\overset{ˉ}{x}$ = 503.4 × 16.3 µm, *n* = 20), rigid, brown to black, cylindrical or subulate, composed of compact conidiophores. *Conidiogenous cells* 11–39 × 1.5–2.7 µm ($\overset{ˉ}{x}$ = 20.5 × 2 µm, *n* = 20), integrated, terminal to intercalary, polyblastic, sympodial, cylindrical, curved, hyaline to pale brown, covered with small denticulate, hyaline conidiogenous loci. *Conidia* 5–9.8 × 3–4.5 µm ($\overset{ˉ}{x}$ = 7.5 × 3.7 µm, *n* = 50), acrogenous, solitary, ellipsoidal to obovoidal, smooth-walled, rounded apical, slightly taper at the base, hyaline, aseptate, straight, guttulate. **Sexual morph**: Undetermined.

Culture characteristics: Conidia germinating on PDA within 24 h, with germ tubes produced from both ends. Colonies on PDA reaching 15 mm in diameter after 4 weeks at room temperature. Mycelia dry, dense. Colonies on the surface of PDA, with irregular edges, surface rough, with slightly protrusion central, brownish-yellow, dark brown, smooth from below. *Conidiophores* reduced or arising from aerial hyphae, macronematous, mononematous or synnematous, cylindrical, straight or slightly flexural, branched, hyaline to brown, paler towards the apex. *Synnemata* 322–690 × 4.2–15 µm ($\overset{ˉ}{x}$ = 446.8 × 8.7 µm, *n* = 10), erect, slightly flexural, hyaline to pale brown, subcylindrical, composed of compact or incompact appressed conidiophores. *Conidiogenous cells* 5.9–29 × 1.7–4.5 µm ($\overset{ˉ}{x}$ = 15.5 × 2.7 µm, *n* = 30), polyblastic, terminal, integrated, cylindrical, curved, hyaline, smooth-walled, with several denticulate conidiogenous loci at the expanded apex. *Conidia* 6.3–8.4 × 2.7–4.6 µm ($\overset{ˉ}{x}$ = 7.3 × 3.7 µm, *n* = 30), acrogenous, solitary, obovoidal, straight, broadly rounded at the apex, aseptate, hyaline, smooth-walled, guttulate. *Chlamydospores* 6.3–11 × 4–8.1 µm ($\overset{ˉ}{x}$ = 7.9 × 5.1 µm, *n* = 30), produced from hyphae, catenate, intercalary, lateral to terminal, obovoidal to subglobose, thin, smooth-walled, aseptate, hyaline to pale brown. *Secession schizolytic*.

Material examined: China, Yunnan Province, Xishuangbanna Autonomous Prefecture, Jinghong, Lancangjiang River basin (22°42′96.98″N; 100°59′34.67″E), on unknown submerged decaying wood in a freshwater stream, 14 July 2022, Long-Li Li, S-3941 (HKAS 131969), living cultures, CGMCC 3.27016 = KUNCC 23-15489; Guangxi Autonomous Region, Baise (24°08′90.39″N; 106°64′83.78″E), on unknown submerged decaying wood in a freshwater stream, 18 February 2024, Zheng-Quan Zhang, S-6267 (HKAS 139433), living culture, KUNCC 24-18211.

Notes: Phylogenetic analysis showed that our new collections clustered with two strains of *Phaeoisaria motuoensis* (KUNCC 10410 and KUNCC 10450) with 100% ML/1.00 PP support (Figure 1). Morphological evidence also supported identification of our new collections as *P. motuoensis*, a species introduced by Xu et al. (2024a), collected from freshwater habitats in Xizang, China. Our two new collections were collected from Guangxi (KUNCC 24-18211) and Yunnan Province (KUNCC 23-15489), respectively, and the morphological characteristics in culture of this species are provided in this study.

***Phaeoisaria obovata*** W.P. Wang, H.W. Shen & Z.L. Luo, J. Fungi 10, 516: 10 (2024), Figure 16

**Figure 16.**
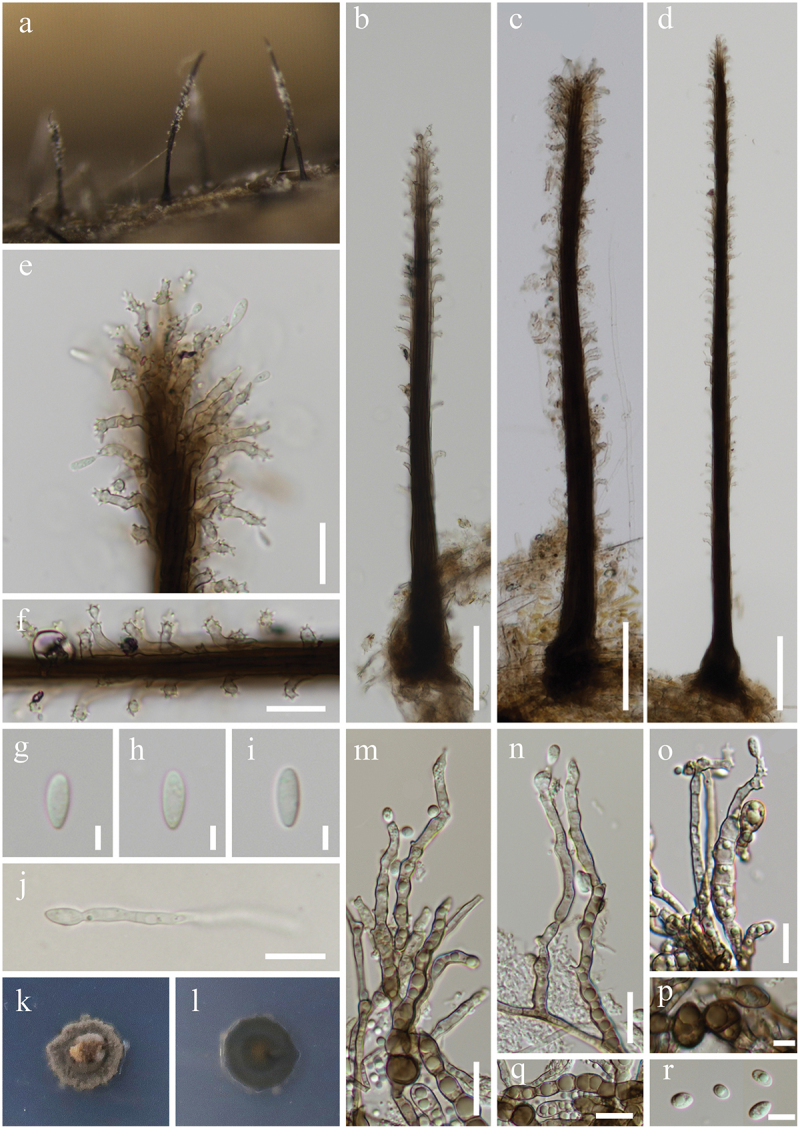
*Phaeoisaria obovata* (HKAS 139429). (a) Colonies on the substratum. (b–d) Synnemata. (e, f) Conidiogenous cells. (g–i) Conidia. (j) Germinating conidium. (k, l) Colony on PDA from surface and reverse. (m–r) Sporulation observed on PDA [(m–o) Conidiophores with conidia; (p, q) Chlamydospores; (r) Conidia]. Scale bars: b–d = 50 µm, e, f, j, m–o, q = 10 µm, g–i = 3 µm, p, r = 5 µm.

*Fungal Names number*: FN571975.

*Saprobic* on submerged decaying wood. **Asexual morph**: *Colonies* erect, hairy, scattered, dark brown to black synnemata covered by white, velvety conidia. *Mycelium* mostly immersed, composed of septate, smooth, branched, hyaline to pale brown hyphae. *Conidiophores* macronematous, synnematous, cylindrical, septate, unbranched, straight, brown to dark brown, paler towards the apex, smooth, thick-walled. *Synnemata* 391–870 × 12–22 µm ($\overset{ˉ}{x}$ = 604.7 × 17.1 µm, *n* = 10), rigid or slightly flexural, brown to black, cylindrical, composed of compact appressed conidiophores. *Conidiogenous cells* 3.6–17.2 × 1.3–2.7 µm ($\overset{ˉ}{x}$ = 11 × 2.1 µm, *n* = 30), integrated, terminal, polyblastic, cylindrical, curved to recurved, sympodial, hyaline, percurrent proliferating, with several denticulate, hyaline conidiogenous loci. *Conidia* 4.1–7.5 × 1.9–2.8 µm ($\overset{ˉ}{x}$ = 5.7 × 2.4 µm, *n* = 30), acrogenous, solitary, obovoidal, smooth-walled, rounded apical and slightly tapering towards to the base, hyaline, aseptate, straight. **Sexual morph**: Undetermined.

Culture characteristics: Conidia germinating on PDA within 24 h, with germ tubes produced from the apex. Colonies on PDA reaching 15 mm in diameter after 4 weeks at room temperature. Mycelia dry, dense. Colonies on the surface of PDA, with regular edges, surface rough, with slightly protrusion central, brown to dark green, circular, brown to dark green, smooth from below. *Conidiophores* 27–51 × 2.4–4 µm ($\overset{ˉ}{x}$ = 40.4 × 3 µm, *n* = 30), reduced or macronematous, mononematous, produced from hyphae or chain-like chlamydospores, cylindrical with irregular shrinkage or swelling, slightly flexural, hyaline to pale brown, paler towards the apex. *Conidiogenous cells* 8.8–27 × 1.8–3.8 µm ($\overset{ˉ}{x}$ = 17.2 × 2.5 µm, *n* = 30), polyblastic, terminal, integrated, subcylindrical, curved, hyaline to pale brown, with one to several denticulate conidiogenous loci. *Conidia* 3.1–6.5 × 2.1–3.6 µm ($\overset{ˉ}{x}$ = 4.8 × 2.8 µm, *n* = 30), acrogenous, solitary, obovoidal to subglobose, straight, rounded apical and basal, aseptate, hyaline, smooth-walled, guttulate. *Chlamydospores* 5–11 × 4.1–8.3 µm ($\overset{ˉ}{x}$ = 8.2 × 5.8 µm, *n* = 20), produced from hyphae, catenate as chain-like or solitary, intercalary or terminal, subcylindrical or obovoidal to subglobose, thin or thick, smooth-walled, aseptate, hyaline to brown, guttulate. *Secession schizolytic*.

Material examined: China, Guangxi Autonomous Region, Hechi (24°54′91.53″N; 106°96′68.56″E), on unknown submerged decaying wood in a freshwater stream, 19 February 2024, Fa-Li Li, S-6060 (HKAS 139429), living culture, KUNCC 24-17750.

Notes: Phylogenetic analysis showed that the new collection (KUNCC 24-17750) clustered with two strains of *Phaeoisaria obovata* (CGMCC 3.27015 and KUNCC 23-15598) with 100% ML/1.00 PP support (Figure 1), and the new collection fits well with the reported descriptions of *P. obovata*. We therefore identify the new collection as *P. obovata*, a species introduced by Wang et al. (2024a) in Yunnan Province, China. In this study, the new collection of *P. obovata* was collected from Guangxi, China, and the morphological characteristics in culture of this species are provided in this study.

***Phaeoisaria sedimenticola*** X.L. Cheng & Wei Li ter, Mycotaxon 127(1): 20 (2014)

*Fungal Names number*: FN563661.

Material examined: China, Guangxi Autonomous Region, Yulin (22°24′48.70″N; 109°70′42.37″E), on unknown submerged decaying wood in a freshwater stream, 25 February 2024, Fa-Li Li, S-6316 (HKAS 139434), living culture, KUNCC 24-18239.

***Pleurotheciella*** Réblová, Seifert & J. Fourn.

Notes: *Pleurotheciella* is a typical freshwater fungal genus introduced by Réblová et al. (2012), with *P. rivularia* as the type species. Twenty-four species are accepted in *Pleurotheciella*, only *P. acericola*, *P. dimorphospora*, and *P. hyalospora* have been collected from terrestrial habitat, and the area with the highest concentration of this genus is Yunnan Province, China (Réblová et al. 2012; Hyde et al. 2018; Luo et al. 2018a; Boonmee et al. 2021; Shi et al. 2021; Visagie et al. 2024; Wang et al. 2024a). Asexual morph of *Pleurotheciella* is characterised by reduced or macronematous, mononematous or synnematous, cylindrical, mostly unbranched, septate, paler towards the apex conidiophores, terminal or intercalary conidiogenous cells with denticulate conidiogenous loci, and solitary, hyaline, clavate to fusoid, straight or curved conidia (Hyde et al. 2018; Shi et al. 2021; Visagie et al. 2024). Sexual morph of *Pleurotheciella* is characterised by semi-immersed to superficial, ellipsoidal ascomata with a lateral neck, cylindrical to clavate, truncate or rounded apical asci with an apical ring, and fusoid-to-fusiform, hyaline, septate ascospores (Réblová et al. 2012, 2020; Luo et al. 2018a; Wang et al. 2024a).

***Pleurotheciella aquatica*** Z.L. Luo, D.J. Bhat, H.Y. Su & K.D. Hyde, Mycol. Prog. 17 (5): 517 (2018) Figure 17

**Figure 17.**
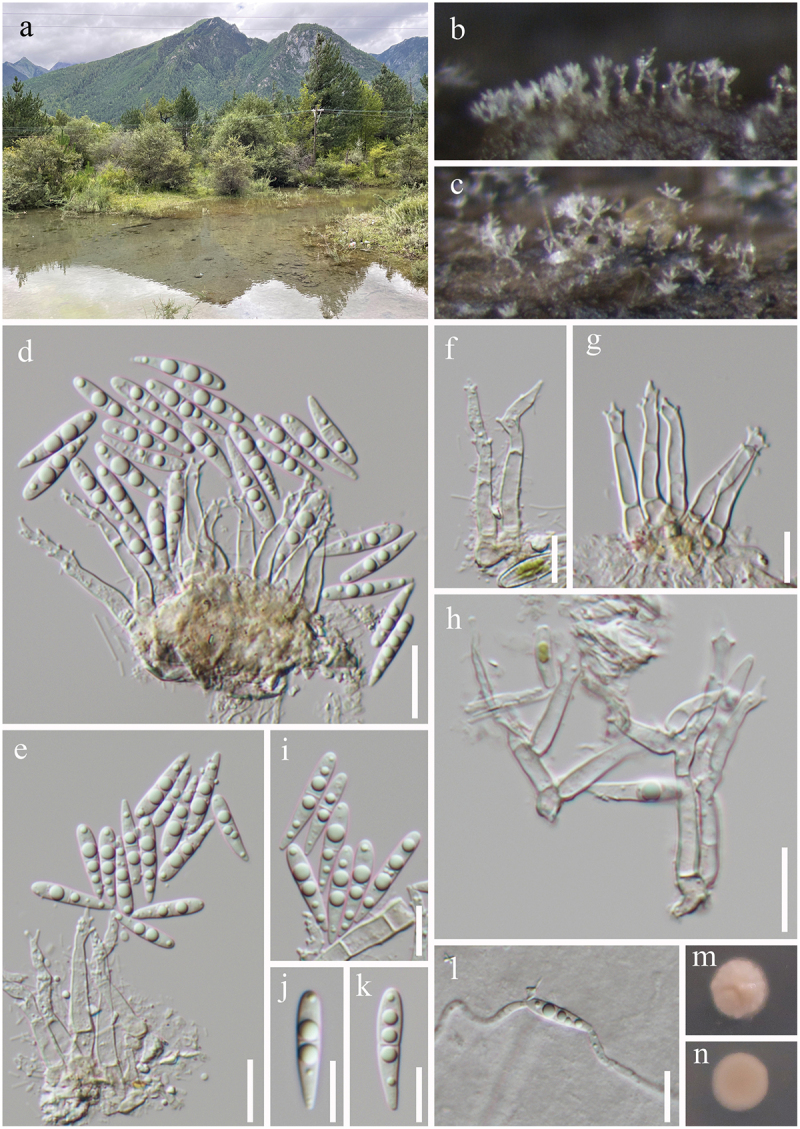
*Pleurotheciella aquatica* (HKAS 146440). (a) Freshwater habitat. (b, c) Colonies on the substratum. (d, e) Conidiophores with conidia. (f–h) Conidiophores. (i–k) Conidia. (l) Germinating conidium. (m, n) Colony on PDA from surface and reverse. Scale bars: d, e, h = 15 µm, f, g, i–l = 10 µm.

*Fungal Names number*: FN821838.

Material examined: China, Xizang Autonomous Region, Linzhi, Bomi County (29°55′46.58″N; 95°38′21.76″E), on unknown submerged decaying wood in a freshwater stream, 26 July 2024, Zong-Long Luo, S-6728 (HKAS 146440), living culture, KUNCC 24-19069; *ibid*., S-6729 (HKAS 146444), living culture, KUNCC 24-19070.

**Figure 18.**
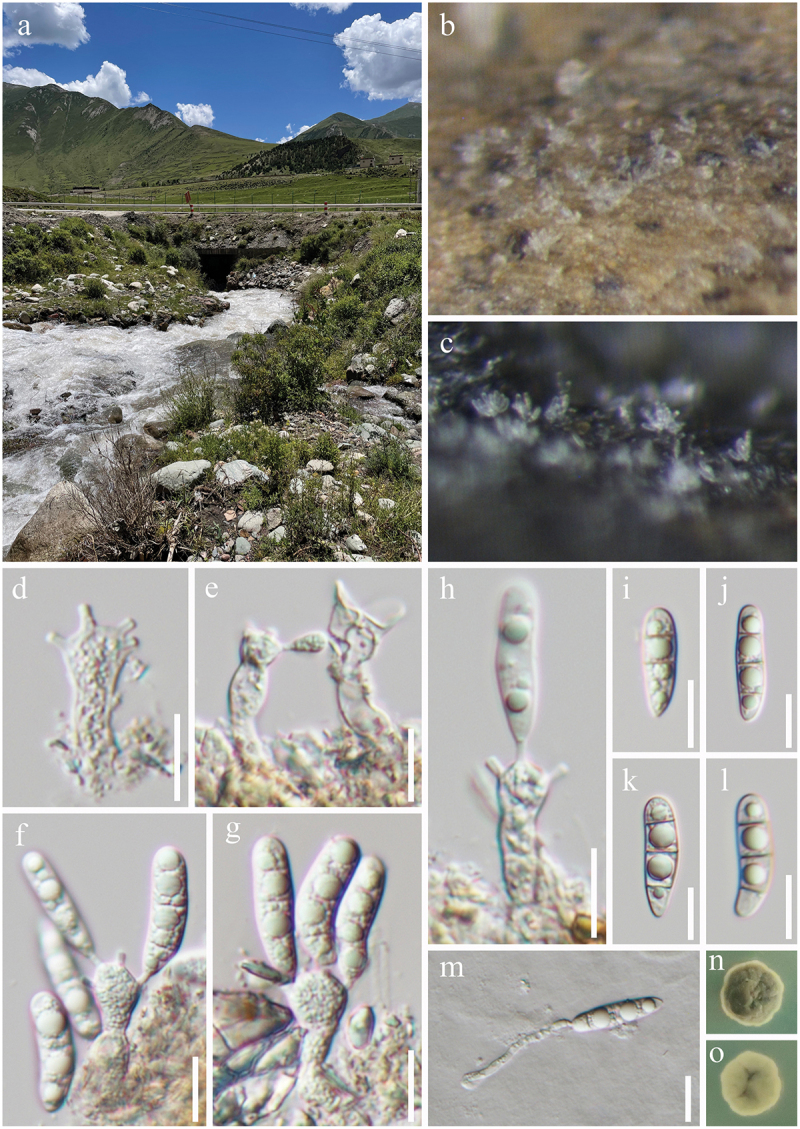
*Pleurotheciella atroseptata* (HKAS 146437, holotype). (a) Freshwater habitat. (b, c) Colonies on the substratum. (d–h) Conidiogenous cells, conidiogenous cells with conidia. (i–l) Conidia. (m) Germinating conidium. (n, o) Colony on PDA from surface and reverse. Scale bars: d–m = 10 µm.

***Pleurotheciella atroseptata*** W.P. Wang & Z.L. Luo, sp. nov., Figure 18

*Fungal Names number*: FN572471.

*Etymology*: The epithet “atroseptata” means dark septum, which refers to the conidia with dark septum of this fungus.

Holotype: HKAS 146437.

*Saprobic* on submerged decaying wood. **Asexual morph**: *Colonies* superficial, effuse, scattered, subhyaline. *Mycelium* immersed, composed of smooth, hyaline hyphae. *Conidiophores* reduced to conidiogenous cells. *Conidiogenous cells* 10–21 × 3.5–4.7 µm ($\overset{ˉ}{x}$ = 17.1 × 4.1 µm, *n* = 10), polyblastic, integrated, terminal, hyaline, subcylindrical, straight to slightly curved, partly dilated to subglobose at the apex, with several small cylindrical, 1.5–2.6 µm long, denticulate conidiogenous loci. *Conidia* 16–23 × 4.4–6.7 µm ($\overset{ˉ}{x}$ = 21 × 5.4 µm, *n* = 30), acrogenous, solitary, clavate with rounded apical and acute or obtuse basal, straight to slightly curved, hyaline to subhyaline, smooth-walled, 2–3-septate, the colour of the septum gradually darkens as it develops, guttulate. **Sexual morph**: Undetermined.

Culture characteristics: Conidia germinating on PDA within 24 h, with germ tubes produced from the apex. Colonies on PDA reaching 10 mm in diameter after 12 weeks at room temperature. Mycelia dry, dense. Colonies on the surface of PDA, circular, with regular edges, bulge with rugae, dark brown with pale edges, smooth, pale brown to brown from below.

Material examined: China, Xizang Autonomous Region, Naqu (31°31′40.86″N; 93°32′18.72″E), on unknown submerged decaying wood in a freshwater stream, 29 July 2024, Wen-Peng Wang, S-6944 (HKAS 146437, holotype), ex-type culture, CGMCC 3.28760 = KUNCC 25-19280; *ibid*., S-6946 (HKAS 146441, paratype).

Notes: In the phylogenetic analysis, *Pleurotheciella atroseptata*, *P. centenaria*, *P. erumpens*, *P. ganzhouensis*, *P. irregularis*, *P. rivularia*, *P. submersa*, and *P. xizangnsis* nested within a subclade of *Pleurotheciella* (Figure 1). These species mostly share reduced conidiophores (except *P. submersa*). However, *P. atroseptata* has clavate conidia with rounded apical and acute or obtuse basal differ from *P. rivularia* and *P. centenaria*. Conidia of *P. erumpens*, *P. ganzhouensis*, *P. irregularis*, and *P. xizangnsis* are fusiform, cylindrical or subclavate with pale septa, while the conidia septa of our new species *P. atroseptata* are gradually darkened as it develops, which can be different from those species (Réblová et al. 2012, 2020; Luo et al. 2018a; He et al. 2024). Comparisons of the ITS sequences between *P. atroseptata* and the holotype or ex-type strain of *P. centenaria*, *P. erumpens*, *P. ganzhouensis*, *P. irregularis*, *P. rivularia*, *P. submersa*, and *P. xizangensis* showed 7.06% (35/496 bp, four gaps), 5.75% (29/504 bp, six gaps), 7.34% (40/545 bp, nine gaps), 6.63% (36/543 bp, six gaps), 5.44% (27/496 bp, two gaps), 5.75% (31/539 bp, five gaps), and 5.17% (28/542 bp, four gaps) nucleotide difference, respectively.

***Pleurotheciella brachyspora*** W.P. Wang, H.W. Shen & Z.L. Luo, J. Fungi 10, 516: 15 (2024)

*Fungal Names number*: FN571976.

Material examined: China, Guangxi Autonomous Region, Hezhou (24°42′86.71″N; 111°03′74.02″E), on unknown submerged decaying wood in a freshwater stream, 22 February 2024, Tian-Tian Zhao, S-6343 (HKAS 139463), living culture, KUNCC 24-18257.

**Figure 19.**
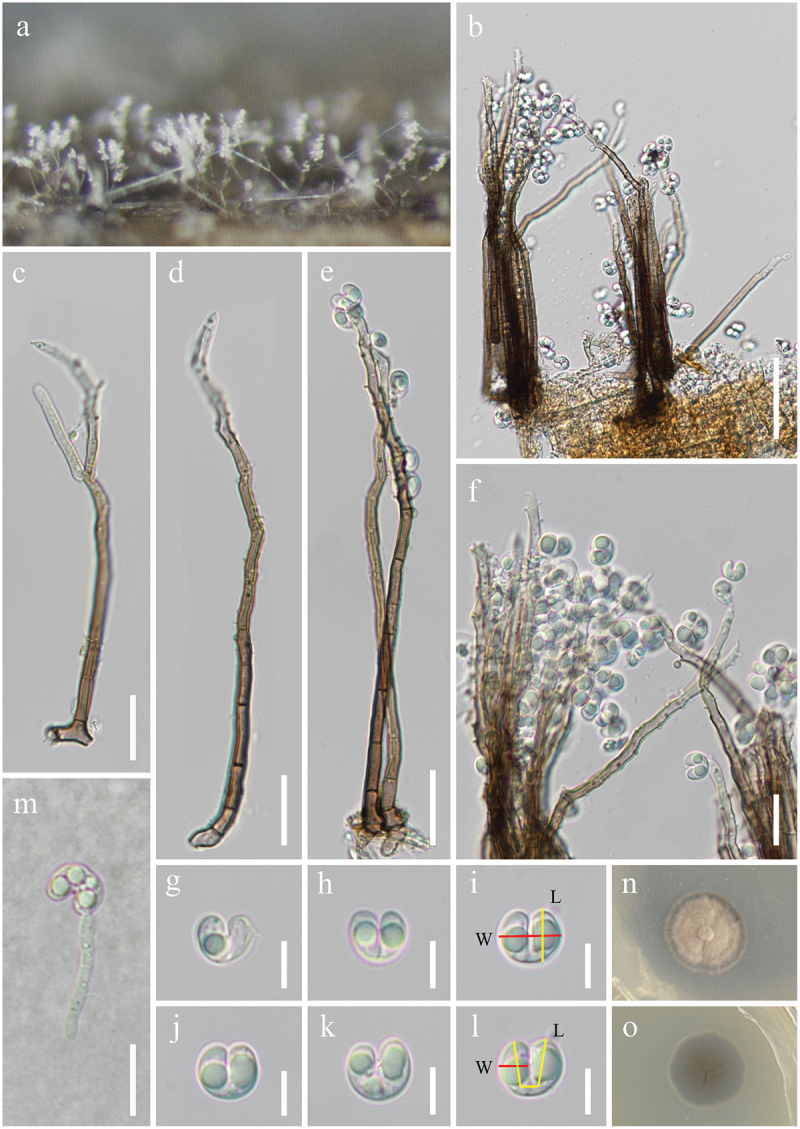
*Pleurotheciella curvata* (HKAS 131967, holotype). (a) Colonies on the substratum. (b) Synnemata. (c, d) Conidiophores. (e) Conidiophores with conidia. (f) Apex of synnemata. (g–l) Conidia. (m) Germinating conidium. (n, o) Colony on PDA from surface and reverse. Scale bars: b = 30 µm, c–e = 15 µm, f, m = 10 µm, g–l = 5 µm.

***Pleurotheciella curvata*** W.P. Wang & Z.L. Luo, sp. nov., Figure 19

*Fungal Names number*: FN572354.

*Etymology*: Referring to the obviously curved conidia of this fungus.

*Holotype*: HKAS 131967.

*Saprobic* on submerged decaying wood. **Asexual morph**: *Colonies* superficial, erect, scattered, brown conidiophores covered with white, velvety conidia. *Mycelium* mostly immersed, composed of septate, smooth, unbranched, pale brown hyphae. *Conidiophores* 65–153 × 2.2–3.1 µm ($\overset{ˉ}{x}$ = 114.1 × 2.7 µm, *n* = 20), macronematous, synnematous or mononematous, erect, cylindrical, slightly flexuous, unbranched or branched, septate, brown, paler towards the apex, hyaline at the apex, with cylindrical denticles covered from the apex to middle. *Synnemata* brown, paler towards the apex, composed of incompact conidiophores. *Conidiogenous cells* polyblastic, integrated, terminal to intercalary, with small, cylindrical, hyaline to pale brown denticulate conidiogenous loci. *Conidia* 4.9–6.7 × 5.9–7.5 µm ($\overset{ˉ}{x}$ = 6 × 6.8 µm, *n* = 30) in general (Figure 19i), 7.3–13 × 2.3–3.7 µm ($\overset{ˉ}{x}$ = 11.2 × 3 µm, *n* = 30) (Figure 19l), acropleurogenous, solitary, straight, bends to U-shaped, the periphery is semicircular, hyaline, guttulate, smooth-walled, rounded at both ends, aseptate, occasionally uniseptate. **Sexual morph**: Undetermined.

Culture characteristics: Conidia germinating on PDA within 24 h, with germ tubes produced from one end. Colonies on PDA reaching 20 mm in diameter after 4 weeks at room temperature. Mycelia dry, dense. Colonies on the surface of PDA, circular, with regular edges, rough surface with a subglobose central protrusion, grey with brown edges, sectored from the centre, brown to dark brown with pale edges from below.

Material examined: China, Yunnan Province, Pu’er (22°71′62.42″N; 100°98′91.43″E), on unknown submerged decaying wood in a freshwater stream, 11 July 2022, Long-Li Li, S-3979 (HKAS 131967, holotype), ex-type cultures, CGMCC 3.27020 = KUNCC 23-15455.

Notes: In the phylogenetic analysis, *Pleurotheciella curvata*, *P. acericola*, *P. brachyspora*, *P. dimorphospora*, *P. longidenticulata*, *P. obliqua*, *P. saprophytica*, and *P. yunnanensis* clustered together as a subclade in *Pleurotheciella* with 100% ML/1.00 PP support (Figure 1). All these species except *P. dimorphospora* and *P. yunnanensis* share macronematous, erect conidiophores with cylindrical denticles covered from the apex to middle. However, *P. curvata* has synnematous or mononematous conidiophores and bends with U-shaped conidia that differs from *P. acericola*, *P. brachyspora*, *P. longidenticulata*, *P. obliqua*, and *P. saprophytica* (Luo et al. 2018a; Boonmee et al. 2021; Wang et al. 2024a; Li et al. 2025). Comparisons of the ITS sequences between *P. curvata* and the ex-type strain of *P. acericola*, *P. brachyspora*, *P. dimorphospora*, *P. longidenticulata*, *P. obliqua*, *P. saprophytica*, and *P. yunnanensis* showed 4.81% (25/520 bp, seven gaps), 5.95% (31/521 bp, seven gaps), 4.61% (24/521 bp, nine gaps), 5.54% (29/523 bp, ten gaps), 4.41% (23/521 bp, nine gaps), 5.03% (25/497 bp, ten gaps), and 5.02% (25/498 bp, nine gaps) nucleotide difference, respectively.

***Pleurotheciella erumpens*** Réblová & J. Fourn., Stud. Mycol. 95: 456 (2020), Figure 20

**Figure 20.**
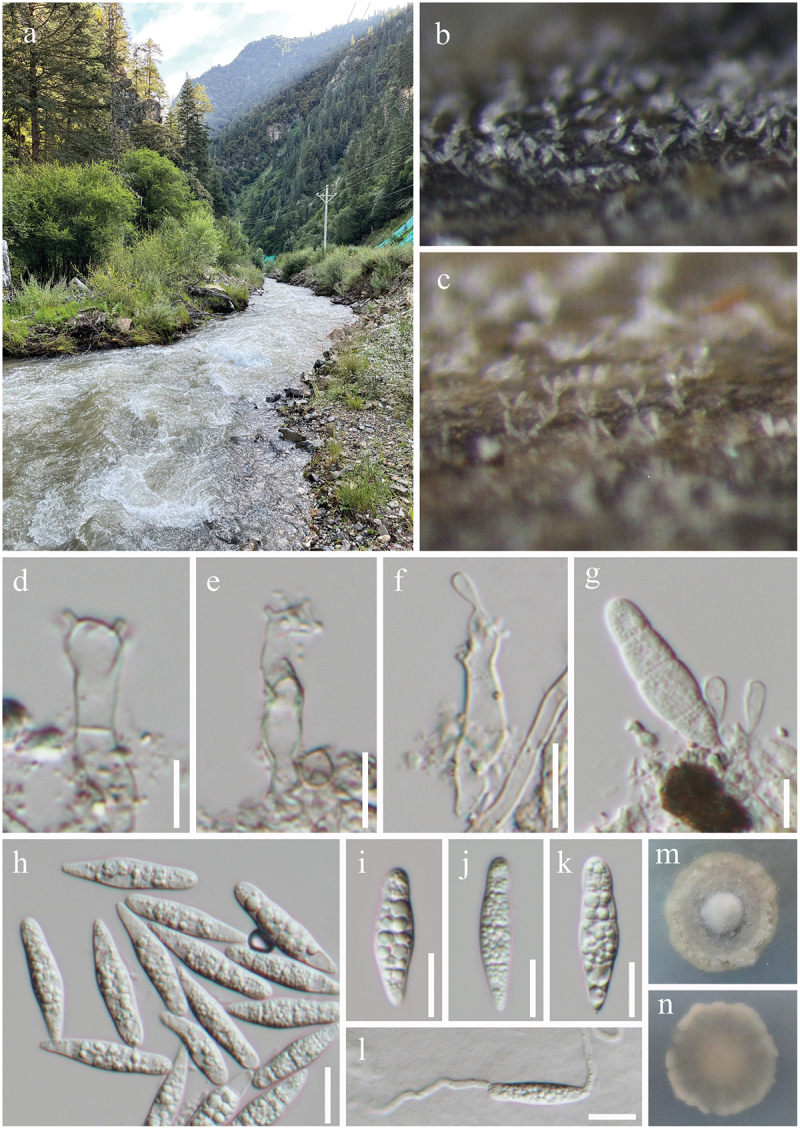
*Pleurotheciella erumpens* (HKAS 146436). (a) Freshwater habitat. (b, c) Colonies on the substratum. (d–g) Conidiogenous cells, conidiogenous cells with conidia. (h–k) Conidia. (l) Germinating conidia. (m, n) Colony on PDA from surface and reverse. Scale bars: d, g = 5 µm, e, f, h–l = 10 µm.

*Fungal Names number*: FN832932.

*Saprobic* on submerged decaying wood. **Asexual morph**: *Colonies* superficial, effuse, scattered, subhyaline to pale white. *Mycelium* immersed, composed of aseptate, smooth, hyaline hyphae. *Conidiophores* reduced to conidiogenous cells. *Conidiogenous cells* 7.2–25 × 3–4.3 µm ($\overset{ˉ}{x}$ = 13.4 × 3.7 µm, *n* = 20), polyblastic, integrated, terminal, hyaline, subcylindrical, straight to slightly curved, with several small cylindrical, up to 1.6 µm long, denticulate conidiogenous loci. *Conidia* 20–27 × 4–6.8 µm ($\overset{ˉ}{x}$ = 23.9 × 5.4 µm, *n* = 50), acrogenous, solitary, fusiform to clavate, broadest in the middle, tapering to the base, with rounded apical and acute or obtuse basal, straight to slightly curved, hyaline, 0–3 inconspicuousness septate, slightly constricted at the septum, multiple small guttulate. **Sexual morph**: Undetermined.

Culture characteristics: Conidia germinating on PDA within 12 h, with germ tubes produced from both ends. Colonies on PDA reaching 20 mm in diameter after 6 weeks at room temperature. Mycelia dry, dense. Colonies semi-immersed in PDA, circular, with regular edges, surface flat, with a globose, velvety, white protrusion in the centre, brownish-grey, brown with pale edges from below.

Material examined: China, Sichuan Province, Ganzi Autonomous Prefecture, Baiyu County (31°10′59.81″N; 98°54′27.91″E), on unknown submerged decaying wood in a freshwater stream, 2 August 2024, Fa-Li Li, S-6794 (HKAS 146436), living culture, KUNCC 24-19088.

Notes: Phylogenetic tree showed that the new collection (KUNCC 24-19088) clustered with the ex-type strain of *Pleurotheciella erumpens* (CBS 142447) with 100% ML/1.00 PP support (Figure 1). Comparisons of the ITS and LSU sequences between our new collection and *P. erumpens* (CBS 142447) revealed 100% (501/501 bp) and 100% (841/841 bp) similarity, respectively. *Pleurotheciella erumpens* was introduced by Réblová et al. (2020), and only described the sexual morph. We identify our new collection as *P. erumpens* based on phylogenetic analysis. Moreover, the asexual morph of *P. erumpens* is provided in this study.

***Pleurotheciella ganzhouensis*** W.M. He, D.M. Hu & H.Y. Song, Front. Microbiol. 15, 1452499: 5 (2024), Figure 21

**Figure 21.**
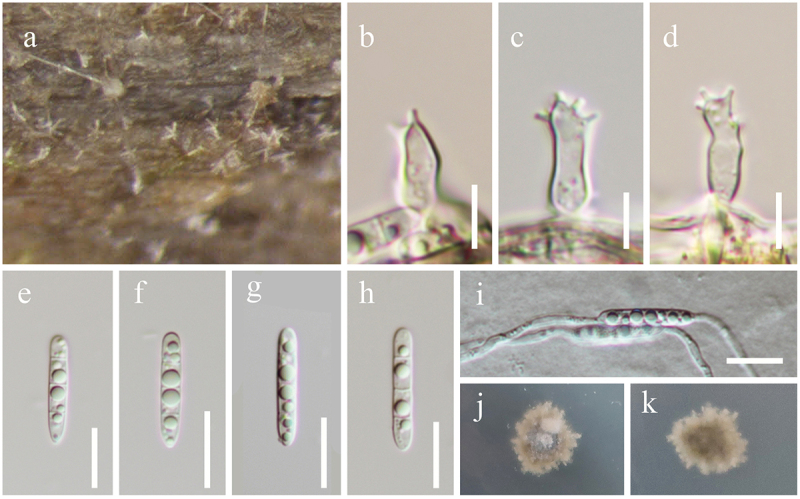
*Pleurotheciella ganzhouensis* (HKAS 139467). (a) Colonies on the substratum. (b–d) Conidiogenous cells. (e–h) Conidia. (i) Germinating conidia. (j, k) Colony on PDA from surface and reverse. Scale bars: b–d = 5 µm, e–i = 10 µm.

*Fungal Names number*: FN853181.

Material examined: China, Yunnan Province, Qujing, Luoping County (25°01′27.11″N; 104°36′75.96″E), on unknown submerged decaying wood in a freshwater river, 15 July 2023, Xing-Ya Zeng, S-5851 (HKAS 139467), living cultures, CGMCC 3.27768 = KUNCC 23-16852.

**Figure 22.**
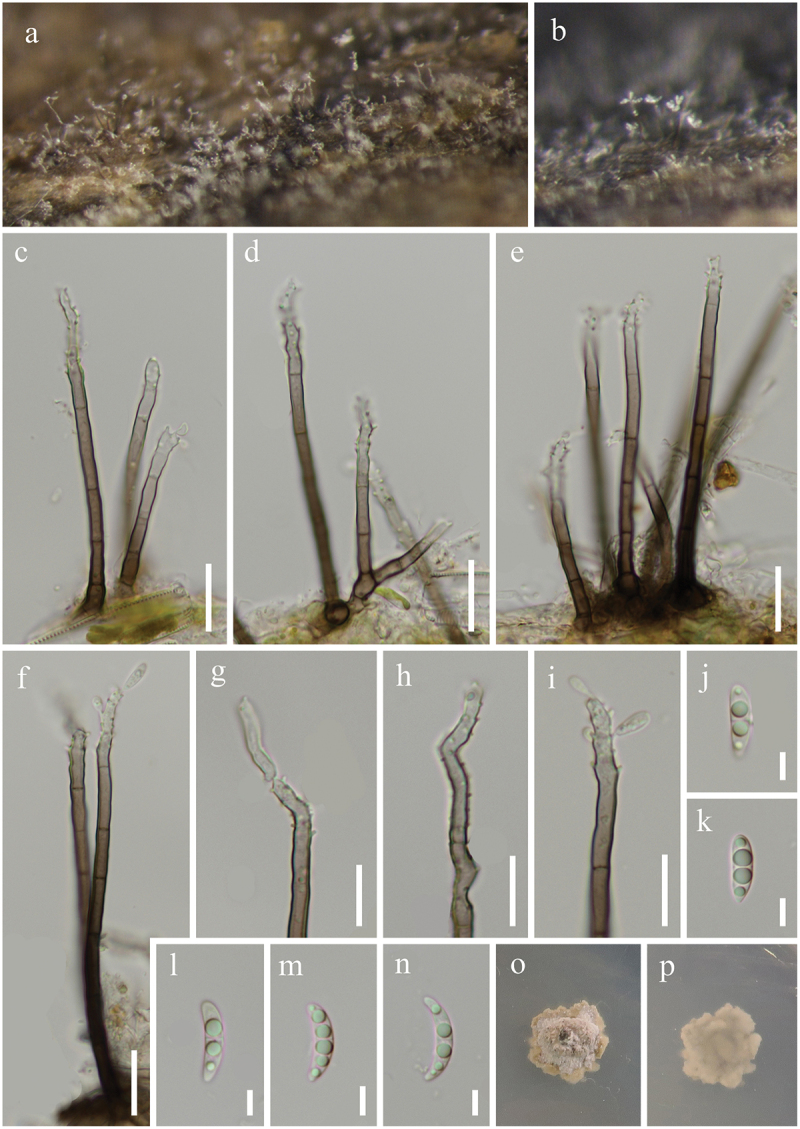
*Pleurotheciella guangxiensis* (HKAS 139461, holotype). (a, b) Colonies on the substratum. (c–f) Conidiophores. (g–i) Conidiogenous cells. (j–n) Conidia. (o, p) Colony on PDA from surface and reverse. Scale bars: c–f = 15 µm, g–i = 10 µm, j–n = 5 µm.

***Pleurotheciella guangxiensis*** W.P. Wang & Z.L. Luo, sp. nov., Figure 22

*Fungal Names number*: FN572355.

*Etymology*: Referring to the Guangxi Autonomous Region, China, where the species was collected.

*Holotype*: HKAS 139461.

*Saprobic* on submerged decaying wood. **Asexual morph**: *Colonies* superficial, effuse, scattered, hairy, brown to dark brown conidiophores with hyaline, gathered conidia at the apex. *Mycelium* mostly immersed, composed of septate, smooth, unbranched, pale brown hyphae. *Conidiophores* 26–109 × 2.4–3.6 µm ($\overset{ˉ}{x}$ = 77.7 × 3.1 µm, *n* = 30), macronematous, mononematous, cylindrical, with a subglobose to globose basal, straight or curved at the apex, occasionally branched at the base, septate, thick, smooth-walled, brown, paler towards the apex, hyaline at the apex, percurrent proliferating, with multiple small denticles at the apex. *Conidiogenous cells* 9.4–33 (−49) × 2.2–3.4 µm ($\overset{ˉ}{x}$ = 25.8 × 2.8 µm, *n* = 30), polyblastic, integrated, determinate or indeterminate, terminal, subcylindrical to cylindrical, straight or curved, hyaline to pale brown, with multiple cylindrical, hyaline, denticulate conidiogenous loci. *Conidia* 12–19 × 2.9–4 µm ($\overset{ˉ}{x}$ = 14.4 × 3.4 µm, *n* = 20), acrogenous, solitary, clavate to lunate or fusiform, hyaline, guttulate, slightly curved, with dull both ends or rounded apical and slightly truncate base, smooth-walled, uniseptate or aseptate. **Sexual morph**: Undetermined.

Culture characteristics: Colonies on PDA reaching 20 mm in diameter after 4 weeks at room temperature. Mycelia dry, dense. Colonies on the surface of PDA, with irregular edges, surface rough, protrusion, khaki.

Material examined: China, Guangxi Autonomous Region, Liuzhou (25°19′72.54″N; 109°23′23.99″E), on unknown submerged decaying wood in a freshwater stream, 20 February 2024, Tian-Tian Zhao, S-6361 (HKAS 139461, holotype), ex-type culture, KUNCC 24-18269.

Notes: In the phylogenetic analysis, *Pleurotheciella guangxiensis* formed a distinct branch basal to *P. aquatica*, *P. fusiformis*, *P. hyalospora*, *P. lunata*, and *P. uniseptata* (Figure 1). Comparisons of the ITS sequences of *P. guangxiensis* with the ex-type strain of *P. aquatica*, *P. fusiformis*, *P. hyalospora*, *P. lunata*, and *P. uniseptata* reveal a difference of 5.12% (28/547 bp, 17 gaps), 5.11% (29/568 bp, 14 gaps), 4.6% (24/522 bp, eight gaps), 6.32% (35/554 bp, seven gaps), and 6.11% (32/524 bp, ten gaps), respectively.

Morphologically, *Pleurotheciella fusiformis* exhibits hyaline conidiophores, whereas those of *P. guangxiensis* are brown and become paler towards the apex (Luo et al. 2018a). The conidiophores of *P. guangxiensis* (26–109 µm) are noticeably longer than those of *P. aquatica* (28–46 µm) and *P. lunata* (26–44 µm), but shorter than those of *P. uniseptata* (100–150 µm) (Réblová et al. 2016; Luo et al. 2018a). Furthermore, *P. guangxiensis* has occasionally branched, percurrent proliferating conidiophores with straight or curved conidiogenous cells, which can be distinguished from *P. hyalospora* (Liu et al. 2024b; Figure 9). We therefore recognise *Pleurotheciella guangxiensis* as a new species.

***Pleurotheciella nilotica*** Abdel-Aziz & Abdel-Wahab, Nova Hed. 110 (1–2): 94 (2020), Figure 23

**Figure 23.**
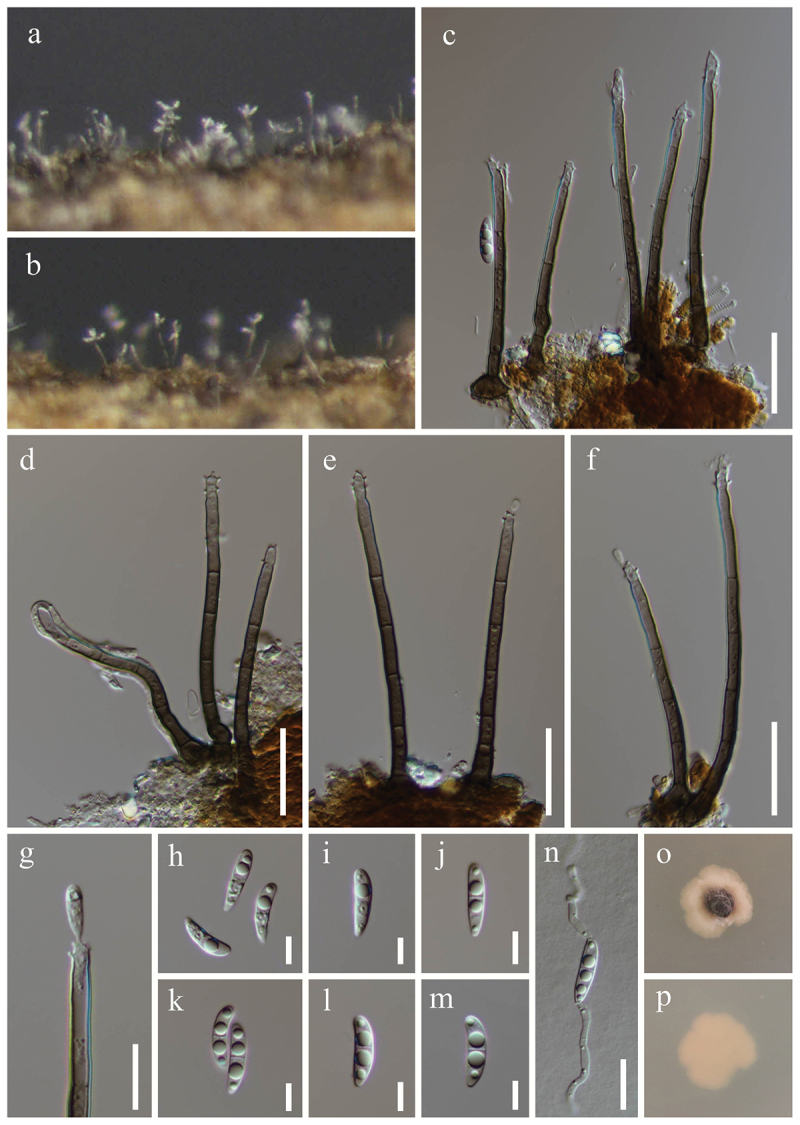
*Pleurotheciella nilotica* (HKAS 139440). (a, b) Colonies on the substratum. (c–f) Conidiophores. (g) Conidiogenous cells with conidia. (h–m) Conidia. (n) Germinating conidium. (o, p) Colony on PDA from surface and reverse. Scale bars: c–f = 20 µm, g, n = 10 µm, h–m = 5 µm.

*Fungal Names number*: FN833765.

*Synonym*: ***Pleurotheciella verrucosa*** W.M. He, D.M. Hu & H.Y. Song, Frontiers in Microbiology 15:1452499, 5 (2024), syn. nov.

*Saprobic* on submerged decaying wood. **Asexual morph**: *Colonies* superficial, erect, gathered in small groups or scattered, hairy, brown to dark brown conidiophores with hyaline conidia at the apex. *Mycelium* mostly immersed, composed of septate, smooth, unbranched, pale brown hyphae. *Conidiophores* 34–85 × 2.2–3.4 µm ($\overset{ˉ}{x}$ = 64.5 × 2.8 µm, *n* = 30), macronematous, mononematous, cylindrical, straight or slightly flexuous, unbranched, septate, brown, paler towards the apex, hyaline to pale brown at the apex, smooth, thick-walled, with multiple denticles at the apex. *Conidiogenous cells* (7.7–) 15–33 × 2.1–2.8 µm ($\overset{ˉ}{x}$ = 21.9 × 2.5 µm, *n* = 30), polyblastic, integrated, terminal, subcylindrical, hyaline to pale brown, sympodial, with several cylindrical, up to 1.3 µm denticulate conidiogenous loci. *Conidia* 12–15 × 2.5–3.5 µm ($\overset{ˉ}{x}$ = 13.7 × 3.1 µm, *n* = 30), acrogenous, solitary, hyaline, guttulate, straight to slightly curved, clavate, with rounded apical, taper to the base, smooth-walled, aseptate when young, uniseptate at maturity. **Sexual morph**: Undetermined.

Culture characteristics: Conidia germinating on PDA within 12 h, with germ tube produced from both ends. Colonies on PDA reaching 15 mm in diameter after 6 weeks at room temperature. Mycelia dry, dense. Colonies on the surface of PDA, with regular edges, pale brown with a dark brown, subglobose protrusion in the centre, pale brown from below.

Material examined: China, Guangxi Autonomous Region, Nanning (22°49′48″N; 108°13′32″E), on unknown submerged decaying wood in a freshwater river, 17 November 2023, Qiu-Xia Yang, S-6325 (HKAS 139440), living culture, KUNCC 24-18245.

Notes: In the phylogenetic analysis, our collection is nested within *Pleurotheciella nilotica*. Comparisons of the ITS and LSU sequences of our collection and *P. nilotica* (KUMCC 19-0214) revealed 99.23% (516/520 bp) and 99.75% (804/806 bp) similarity, respectively. Morphological evidence also supports the identification of our collection as *P. nilotica* (Abdel-Aziz et al. 2020; Shi et al. 2021).

He et al. (2024) introduced *Pleurotheciella verrucosa* from freshwater habitats in Jiangxi Province, China, and the phylogenetic dataset did not include *P. nilotica*. Our phylogenetic analysis showed that two strains of *P. verrucosa* (JAUCC6076 and JAUCC6078) clustered with several strains of *P. nilotica* (KUMCC 19-0214, KUMCC 20-0154, MD1317, and KUNCC 24-18245) with 100% ML/1.00 PP support (Figure 1). Comparisons of the ITS, LSU, and SSU sequences of *P. verrucosa* (JAUCC6076) and *P. nilotica* (KUMCC 19-0214) revealed 99.40% (501/504 bp), 99.76% (820/822 bp), and 99.88% (822/823 bp, one gap) similarity, respectively. Morphologically, *P. verrucosa* is similar to the holotype of *P. nilotica* in having macronematous, mononematous, cylindrical, slightly curved conidiophores with similar sized (51.3–131.8 × 1.9–3.4 µm vs. 23–110 × 3–4 µm), terminal, sympodially conidiogenous cells, and clavate, uniseptate conidia with similar size (10.2–16.9 × 2.3–4.3 µm vs. 8–13 × 2–4 µm) (Abdel-Aziz et al. 2020). We therefore synonymise *Pleurotheciella verrucosa* with *P. nilotica*.

***Pleurotheciella sympodia*** H. Yang & H. Zhang, Phytotaxa 518 (2): 151 (2021), Figure 24

**Figure 24.**
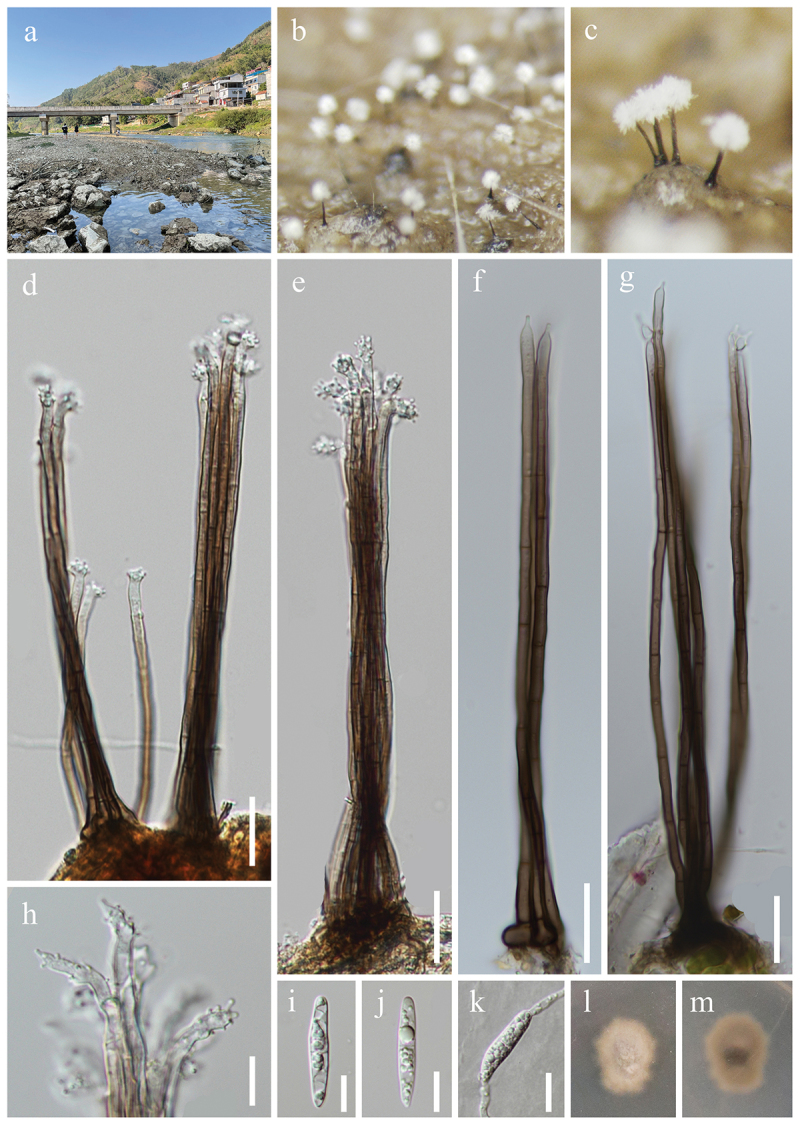
*Pleurotheciella sympodia* (a, f, g, l, m, HKAS 139458; b–e, h–k, HKAS 139452). (a) Freshwater habitat. (b, c) Colonies on the substratum. (d–g) Synnemata. (h) Conidiogenous cells. (i, j) Conidia. (k) Germinating conidium. (l, m) Colony on PDA from surface and reverse. Scale bars: d–g = 20 µm, h–k = 10 µm.

*Fungal Names number*: FN557776.

*Saprobic* on submerged decaying wood. **Asexual morph**: *Colonies* superficial, erect, scattered, solitary or in groups, hairy, brown to dark brown synnemata with white, velvety conidia gathered at the apex. *Mycelium* partly immersed, partly superficial, composed of septate, smooth, unbranched, pale brown hyphae. *Conidiophores* 115–213 × 2.2–3.5 µm ($\overset{ˉ}{x}$ = 158.9 × 2.8 µm, *n* = 20), macronematous, mostly synnematous, partly mononematous, cylindrical, septate, smooth-walled, straight or slightly flexural, unbranched, brown, paler towards the apex, hyaline at the apex. *Synnemata* 120–248 × 6.1–13 µm ($\overset{ˉ}{x}$ = 186 × 10.5 µm, *n* = 10), rigid, brown, cylindrical, composed of incompact to compact conidiophores. *Conidiogenous cells* 23–52 × 2.3–3.3 µm ($\overset{ˉ}{x}$ = 35.3 × 2.8 µm, *n* = 20), polyblastic, integrated, terminal, cylindrical, straight or slightly curved, hyaline to pale brown, sympodial, with one to several cylindrical denticulate. *Conidia* 23–31 × 4–5.9 µm ($\overset{ˉ}{x}$ = 27.6 × 4.9 µm, *n* = 30), acrogenous, solitary, hyaline, straight to slightly curved, clavate, rounded apical and tapering towards the base, smooth-walled, uniseptate, guttulate. **Sexual morph**: Undetermined.

Culture characteristics: Conidia germinating on PDA within 24 h, with germ tubes produced from both ends. Colonies on PDA reaching 10 mm in diameter after 4 weeks at room temperature. Mycelia dry, dense. Colonies on the surface of PDA, with irregular edges, flat surface with protrusion centre, khaki, smooth, khaki with dark brown in the centre from below.

Material examined: China, Yunnan Province, Xishuangbanna Autonomous Prefecture (21°53′37.60″N; 101°16′30.98″E), on unknown submerged decaying wood in a freshwater stream, 12 July 2022, Liang Zhang, S-4266 (HKAS 139452), living culture, KUNCC 23-15478; Guangxi Autonomous Region, Liuzhou (24°08′90.39″N; 106°64′83.78″E), on unknown submerged decaying wood in a freshwater stream, 18 February 2024, Tian-Tian Zhao, S-6423 (HKAS 139458), living culture, KUNCC 24-18298.

Notes: Our new collections fit well with the description of *Pleurotheciella sympodia* in having synnematous conidiophores, terminal, denticulate conidiogenous cells, and clavate, straight or slightly curved conidia with rounded apical (Shi et al. 2021). In the phylogenetic analysis, our new collections nested within the *P. sympodia* clade. Therefore, we identify our new collections as *P. sympodia* based on phylogenetic analysis and morphological characteristics. Shi et al. (2021) introduced *P. sympodia* from freshwater habitats in Thailand, marking the first record of this species in China.

***Pleurotheciella uniseptata*** (Matsush.) Seifert, Persoonia 37: 74 (2016), Figure 25

**Figure 25.**
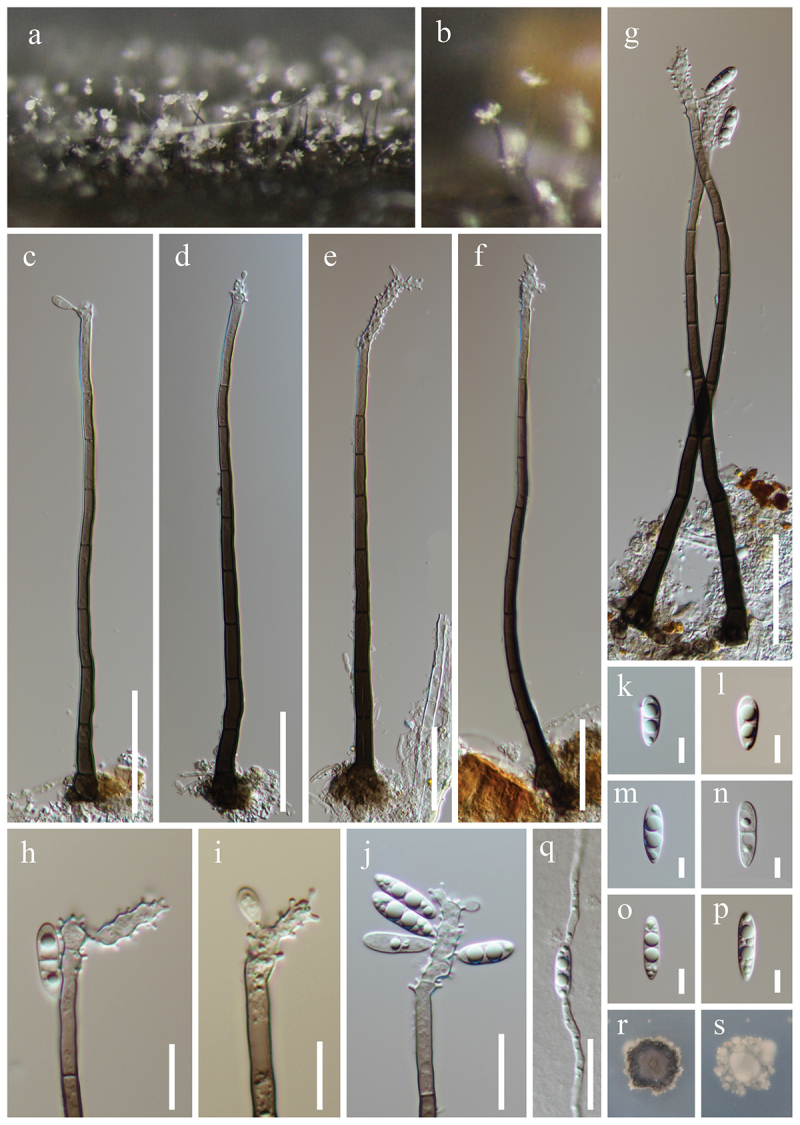
*Pleurotheciella uniseptata* (HKAS 139437). (a, b) Colonies on the substratum. (c–f) Conidiophores. (g) Conidiophores with conidia. (h–j) Conidiogenous cells with conidia. (k–p) Conidia. (q) Germinating conidium. (r, s) Colony on PDA from surface and reverse. Scale bars: c–g = 30 µm, h, i = 10 µm, j, q = 15 µm, k–p = 5 µm.

*Fungal Names number*: FN 813238.

Material examined: China, Yunnan Province, Wenshan Autonomous Prefecture, Guangnan County (24°19′12.23″N; 105°11′67.48″E), on unknown submerged decaying wood in a freshwater stream, 19 July 2023, Wen-Peng Wang, S-5883 (HKAS 139437), living cultures, CGMCC 3.27769 = KUNCC 23-16863; Qujing, Luoping County (24°76′57.73″N; 104°49′72.38″E), on unknown submerged decaying wood in a freshwater river, 16 July 2023, Fa-Li Li, S-5766 (HKAS 139450), living culture, KUNCC 23-16827.

**Figure 26.**
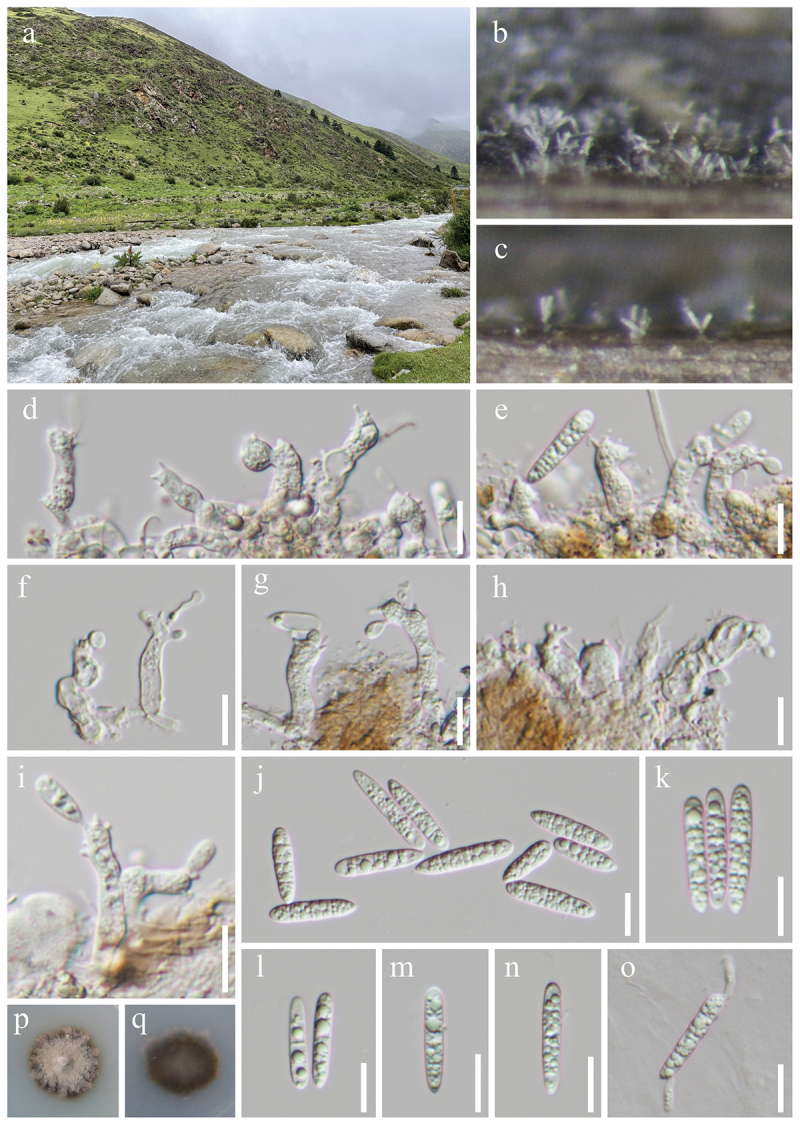
*Pleurotheciella xizangensis* (HKAS 147751, holotype). (a) Freshwater habitat. (b, c) Colonies on the substratum. (d–i) Conidiogenous cells, conidiogenous cells with conidia. (j–n) Conidia. (o) Germinating conidium. (p, q) Colony on PDA from surface and reverse. Scale bars: d–o = 10 µm.

***Pleurotheciella xizangensis*** W.P. Wang & Z.L. Luo, sp. nov., Figure 26

*Fungal Names number*: FN572472.

*Etymology*: Referring to the Xizang Autonomous Region, China, where the species was collected.

Holotype: HKAS 147751.

*Saprobic* on submerged decaying wood. **Asexual morph**: *Colonies* superficial, effuse, scattered, subhyaline to pale white. *Mycelium* immersed, composed of aseptate, smooth, hyaline hyphae. *Conidiophores* reduced to conidiogenous cells. *Conidiogenous cells* 10.5–24 (−36) × 3–4.8 µm ($\overset{ˉ}{x}$ = 18.4 × 3.7 µm, *n* = 30), polyblastic, integrated, terminal, hyaline, subcylindrical, straight to slightly curved, with several small cylindrical denticulate conidiogenous loci 1.6–5.1 µm ($\overset{ˉ}{x}$ = 3.1 µm, *n* = 30). *Conidia* 13–24 × 2.8–4.6 µm ($\overset{ˉ}{x}$ = 20.1 × 3.8 µm, *n* = 50), acrogenous, solitary, clavate to subcylindrical, tapering towards to base, with rounded apical and obtuse to rounded base, straight, hyaline, 0–1 (−2)-septate, guttulate. **Sexual morph**: Undetermined.

Culture characteristics: Conidia germinating on PDA within 12 h, with germ tubes produced from both ends. Colonies on PDA reaching 10 mm in diameter after 6 weeks at room temperature. Mycelia dry, dense. Colonies semi-immersed in the PDA, circular, with irregular edges, surface rough, compaction, brown with a surface covered with a layer of grey hyphae, brown to dark brown from below.

Material examined: China, Xizang Autonomous Region, Changdu (29°38′00.00″N; 98°80′12.76″E), on unknown submerged decaying wood in a freshwater stream, 24 July 2024, Wen-Peng Wang, S-6792 (HKAS 147751, holotype), ex-type culture, KUNCC 25-19266.

Notes: NCBI Blast results of the ITS sequence of *Pleurotheciella xizangensis* (KUNCC 25-19266) showed that the most similar is *P. submersa* (DLUCC 0739, 97.05%). Comparisons of the ITS, LSU, and *rpb*2 sequences between *P. xizangensis* and the ex-type strain of *P. submersa* (MFLUCC 17-1709) revealed 2.96% (16/540 bp, four gaps), 1.13% (8/710 bp, one gap), and 7.47% (77/1,031 bp) difference, respectively. Morphologically, *P. submersa* has macronematous, mononematous, erect, cylindrical conidiophores and subcylindrical, slightly curved, aseptate conidia, while *P. xizangensis* has reduced conidiophores and clavate, 0–1 (−2)-septate, straight, smaller conidia (13–24 × 2.8–4.6 µm vs. 25–28 × 5.5–6.5 µm), the characteristics of which can be distinguished from *P. submersa* (Luo et al. 2018a). We therefore introduce *Pleurotheciella xizangensis* as a new species.

***Pleurothecium*** Höhn.

Notes: *Pleurothecium* was proposed by von Höhnel (1919) and typified by *P. recurvatum*. 18 species are currently accepted in this genus, and among the sexual morphs, only *P. recurvatum* and *P. semifecundum* have been reported (Xu et al. 2025). The asexual morph of *Pleurothecium* is characterised by macronematous, cylindrical, erect or reduced conidiophores, polyblastic, sympodially extended denticulate conidiogenous cells, and cylindrical to ellipsoidal, most 3-septate conidia with obvious guttulate (Luo et al. 2018a; Jayawardena et al. 2022; Fryar and Catcheside 2023; Hyde et al. 2023; He et al. 2024; Liu et al. 2024b).

In the phylogenetic tree, our new species *Pleurothecium pisiformis* and *P. aquisubtropicum* form a separate clade and sister to *Phaeoisaria* (Figure 1). The BLAST search on NCBI GenBank of the ITS sequence of our new species showed that the most similar are *P. aquisubtropicum* (GZAAS 21-0384, 98.84%) and *P. semifecundum* (CBS 131271, 84.96%), respectively. However, *P. pisiformis* fits well with the concept of *Pleurothecium*, and we introduced *P. pisiformis* into this genus. The precise taxonomic status of *P. pisiformis* and *P. aquisubtropicum* still needs to be further determined.

**Figure 27.**
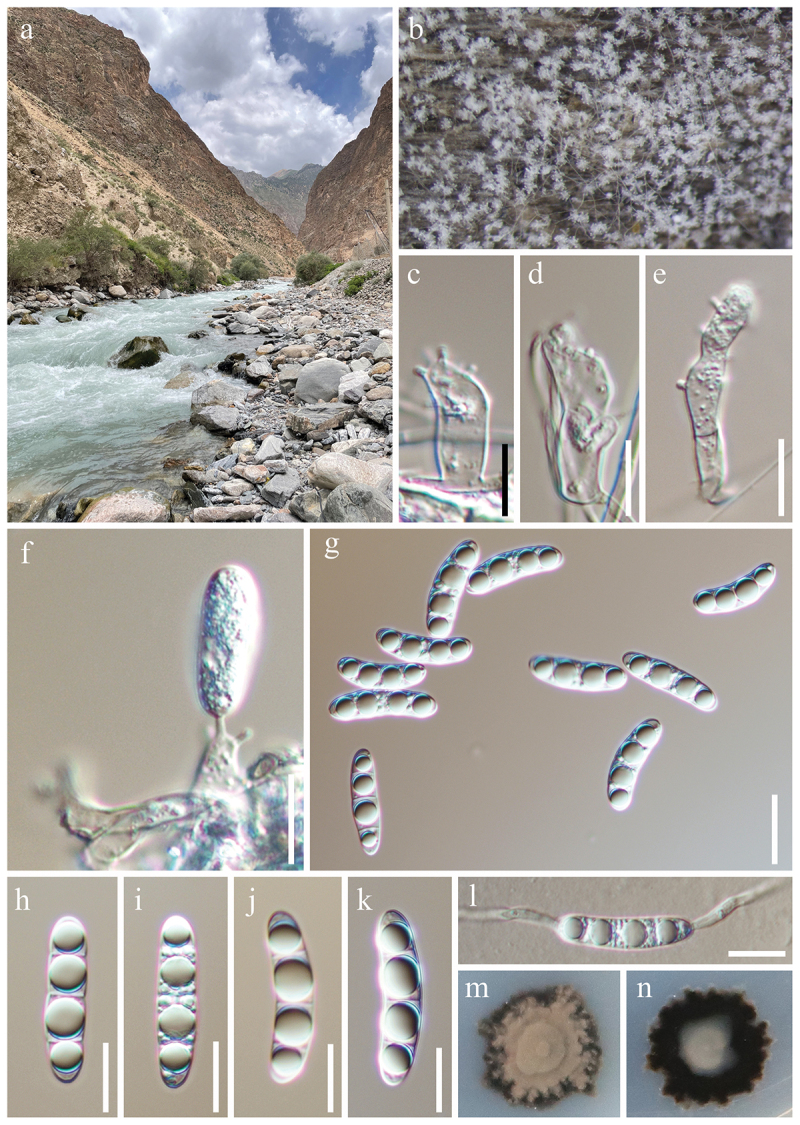
*Pleurothecium pisiformis* (HKAS 146445, holotype). (a) Freshwater habitat. (b) Colonies on the substratum. (c–f) Conidiogenous cells, conidiogenous cells with conidia. (g–k) Conidia. (l) Germinating conidium. (m, n) Colony on PDA from surface and reverse. Scale bars: c–f, h–l = 10 µm, g = 15 µm.

***Pleurothecium pisiformis*** W.P. Wang & Z.L. Luo, sp. nov., Figure 27

*Fungal Names number*: FN572473.

*Etymology*: Referring to the conidia that resembles a pea in shape of this fungus.

*Holotype*: HKAS 146445.

*Saprobic* on submerged decaying wood. **Asexual morph**: *Colonies* superficial, effuse, scattered, white. *Mycelium* partly immersed, partly superficial, composed of septate, smooth, unbranched, hyaline hyphae. *Conidiophores* reduced to conidiogenous cells. *Conidiogenous cells* 11–37 (−46) × 2.5–7.3 µm ($\overset{ˉ}{x}$ = 24.3 × 4.8 µm, *n* = 20), polyblastic, integrated, terminal, hyaline, subcylindrical, straight to slightly curved, thin-walled, with several small cylindrical, 1.5–2.6 µm long, denticulate conidiogenous loci. *Conidia* 21–31 × 4.8–6.9 µm ($\overset{ˉ}{x}$ = 24.9 × 5.8 µm, *n* = 50), acrogenous, solitary, cylindrical, rounded at both ends, straight to slightly curved, hyaline, 3-septate, guttulate, smooth-walled. **Sexual morph**: Undetermined.

Culture characteristics: Conidia germinating on PDA within 12 h, with germ tubes produced from both ends. Colonies on PDA reaching 20 mm in diameter after 6 weeks at room temperature. Mycelia dry, dense. Colonies on the surface of PDA, layer by layer from the outside to the inside, dark brown at base, brown at surface, with irregular edges, pale brown to subhyaline in the centre, becoming dark brown from below.

Material examined: China, Xizang Autonomous Region, Changdu (30°28′31.61″N; 96°39′57.27″E), on unknown submerged decaying wood in a freshwater stream, 31 July 2024, Wen-Peng Wang, S-6789 (HKAS 146445, holotype), ex-type culture, CGMCC 3.28758 = KUNCC 24-19085.

Notes: The phylogenetic tree showed that *Pleurothecium pisiformis* clustered with *P. aquisubtropicum* with 100% ML/1.00 PP support (Figure 1). Comparisons of the ITS and LSU sequences of *P. pisiformis* and *P. aquisubtropicum* revealed 0.96% (5/519, three gaps) and 0.12% (1/844) difference, respectively. However, the two species have completely different morphological characteristics. *Pleurothecium aquisubtropicum* has macronematous, subcylindrical, straight, unbranched conidiophores and aseptate conidia, which are more in line with the concept of *Pleurotheciella* (Jayawardena et al. 2022). *Pleurothecium pisiformis* has reduced conidiophores and cylindrical, rounded at both ends, 3-septate, larger conidia (21–31 × 4.8–6.9 µm vs. 13.4–15.5 × 3.4–5.5 µm). Furthermore, *P. pisiformis* has polyblastic, denticulate conidiogenous cells and cylindrical, rounded at both ends, 3-septate, guttulate conidia fits well with the concept of *Pleurothecium* (Luo et al. 2019; He et al. 2024; Xu et al. 2025). Therefore, we introduce *Pleurothecium pisiformis* as a new species in the genus *Pleurothecium*.

***Rhexoacrodictys*** W.A. Baker & Morgan-Jones

*Synonym*: ***Saprodesmium*** W. Dong & Doilom, syn. nov.

Notes: Baker et al. (2002) established *Rhexoacrodictys* to accommodate the type species, *R. erecta*. To date, *Rhexoacrodictys* comprises six asexual species, which are characterised by macronematous, mononematous, erect conidiophores with percurrent extensions, terminal, monoblastic conidiogenous cells that produce ellipsoidal to obovoidal, muriform conidia (Bao et al. 2022, 2023; Win et al. 2024; Manawasinghe et al. 2025). In this study, we synonymised *Saprodesmium* with *Rhexoacrodictys* based on phylogenetic analysis and morphological characteristics.

***Rhexoacrodictys dematiospora*** (W. Dong, Doilom & K.D. Hyde) W.P. Wang & Z.L. Luo, comb. nov.

*Fungal Names number*: FN572356.

*Basionym*: ***Saprodesmium dematiosporum*** W. Dong, Doilom & K.D. Hyde, J. Fungi 7 (711): 16 (2021).

Holotype: China, Yunnan Province, Pingbian District (22°59′13″N; 103°40′30″E), on decaying wood submerged in an unnamed stream originated from Dawei Mountain Nature Reserve, 20 September 2017, W. Dong, WF23A (HKAS 101710, holotype), ex-type living culture KUMCC 18-0059; *ibid*., MFLU 18-1165, isotype.

Notes: *Saprodesmium dematiosporum* was introduced by Dong et al. (2021) from a freshwater habitat in Yunnan Province, China. In the phylogenetic tree, *S. dematiosporum* nested within *Rhexoacrodictys* and sister to *R. chiangraiensis* (Figure 1), we therefore transfer *S. dematiosporum* to *Rhexoacrodictys* as *R. dematiospora*. Morphologically, *R. dematiospora* has monoblastic conidiogenous cells and obovoidal to ellipsoidal, muriform conidia fits well with the conception of *Rhexoacrodictys*. However, *R. dematiospora* has micronematous, vesiculate, thin-walled conidiophores and subglobose conidiogenous cells, which can be easily distinguished from other *Rhexoacrodictys* species (Xia et al. 2017; Bao et al. 2022, 2023; Win et al. 2024).

***Sterigmatobotrys*** Oudemans

Notes: *Sterigmatobotrys* was introduced by Oudemans (1886), with *S. elata* as the type. Only three species are currently accepted in this genus, *viz*., *Sterigmatobotrys macrocarpa*, *S. rudis*, and *S. uniseptata*, and all *Sterigmatobotrys* species have been collected from freshwater habitats (Luo et al. 2019; Calabon et al. 2022; Yang et al. 2023b). *Sterigmatobotrys* is characterised by septate, branched paraphyses longer than asci, cylindrical, truncate at the apex asci with a distinct refractive, inamyloid apical annulus, fusiform to cylindrical-fusiform, 3-septate ascospores in sexual morph; macronematous, mononematous conidiophores with complex penicillate head, polyblastic, hyaline, parallel conidiogenous cells, and cylindrical to subclavate, slimy conidia gathered in the apex of conidiophores in asexual morph (Réblová and Seifert 2011; Ertz et al. 2016; Luo et al. 2019).

***Sterigmatobotrys rudis*** (Sacc.) Heuchert, U.Braun & Ertz, Fungal Biology 120 (11): 1423 (2016), Figure 28

**Figure 28.**
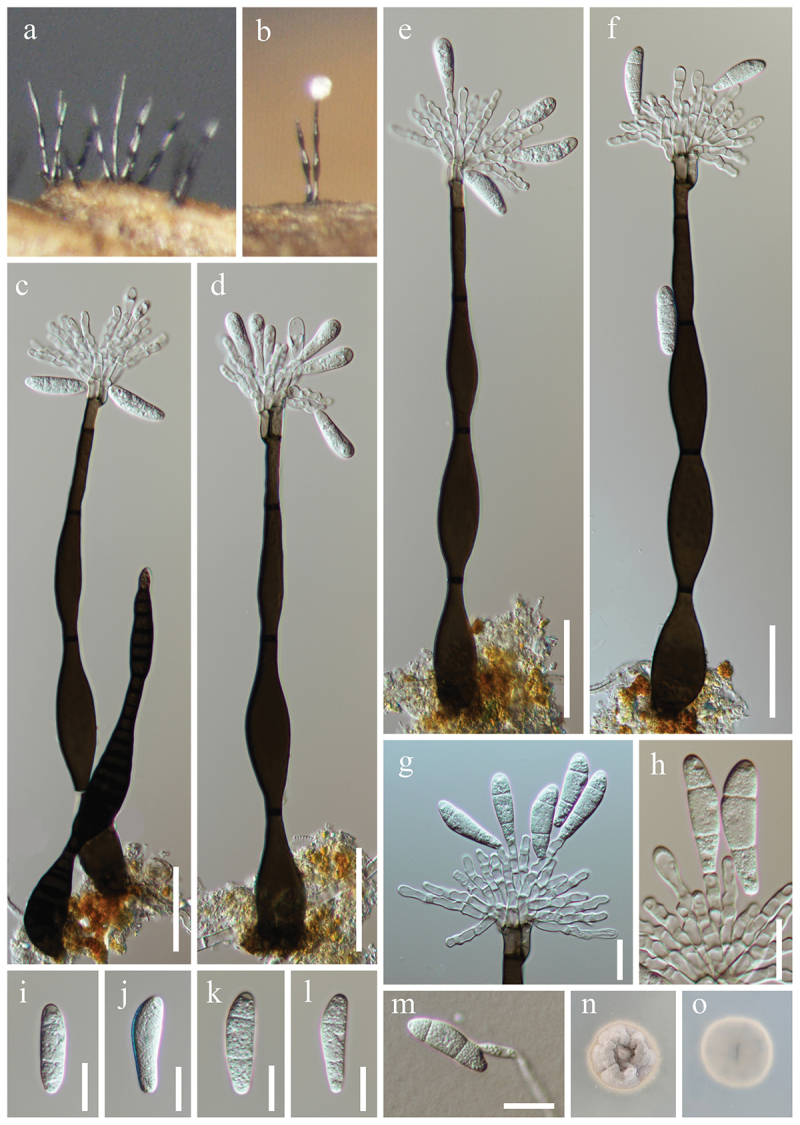
*Sterigmatobotrys rudis* (HKAS 139449). (a, b) Colonies on the substratum. (c–f) Conidiophores with conidia. (g, h) Conidiogenous cells with conidia. (i–l) Conidia. (m) Germinating conidium. (n, o) Colony on PDA from surface and reverse. Scale bars: c–f = 30 µm, g–m = 10 µm.

*Fungal Names number*: FN817106.

*Saprobic* on submerged decaying wood. **Asexual morph**: *Colonies* erect, hairy, scattered, solitary or gathered, hyaline, sometimes slimy conidia gathered at the apex of dark brown to black, glistening conidiophores. *Mycelium* immersed, composed of septate, smooth-walled, hyaline to pale brown hyphae. *Conidiophores* 143–194 × 5.1–6.7 µm ($\overset{ˉ}{x}$ = 169.3 × 5.5 µm, *n* = 10), macronematous, mononematous, dark brown to black, thickened septatum, smooth, thick-walled, consists of ellipsoidal, up to 17 µm wide cells, with a complex penicillate head consisting of series of penicillate branches, partly does not produce conidiogenous cells and conidia, with dense septate. *Conidiogenous cells* 14–27 × 2.2–2.9 µm ($\overset{ˉ}{x}$ = 18.3 × 2.5 µm, *n* = 30), integrated, polyblastic, terminal, cylindrical, hyaline, smooth-walled. *Conidia* 16–24 × 4.1–7 µm ($\overset{ˉ}{x}$ = 20.6 × 5.8 µm, *n* = 30), acrogenous, solitary, slimy, straight or slightly curved at the far end, cylindrical to subclavate, 2-septate without septum, broad at upper middle part, rounded at both ends, smooth, thin-walled, hyaline. **Sexual morph**: Undetermined.

Culture characteristics: Conidia germinating on PDA within 24 h, with germ tubes produced from one side. Colonies on PDA reaching 15 mm in diameter after 4 weeks at room temperature. Mycelia dry, dense. Colonies on the surface of PDA, with regular edges, bulge, surface rough with pucker, central concavity, grey with thin, white edges, circular, grey with white edges from below.

Material examined: China, Yunnan Province, Wenshan Autonomous Region, Guangnan County (24°11′51.30″N; 104°92′82.31″E), on unknown submerged decaying wood in a freshwater stream, 19 July 2023, Xing-Ya Zeng, S-5642 (HKAS 139449), living culture, KUNCC 23-16607; Guangxi Autonomous Region, Guilin (24°52′96.62″N; 100°53′67.92″E), on unknown submerged decaying wood in stream, 22 February 2024, Tian-Tian Zhao, S-6128 (HKAS 139435), living culture, KUNCC 24-18166.

Notes: *Taeniolella rudis* was initially introduced by Hughes (1958), based on phylogenetic analysis and morphological characteristics. Ertz et al. (2016) transferred *T. rudis* to *Sterigmatobotrys* as *S. rudis*. Recently, Yang et al. (2023b) collected *S. rudis* from a freshwater habitat in Yunnan Province, China. However, their description was incomplete. In this study, we recollected *S. rudis* from freshwater habitats in Yunnan Province and Guangxi, and provided a complete morphological description for *S. rudis*. This species also has complex penicillate head of conidiophores and slimy, hyaline, cylindrical to subclavate conidia.

***Savoryellales*** Boonyuen, Suetrong, Sivichai, K.L. Pang & E.B.G. Jones

***Savoryellaceae*** Jaklitsch & Réblová

Notes: *Savoryellaceae* was established by Jaklitsch (2015), with *Savoryella* as the type genus. Hyde et al. (2024a) included eight genera, *viz*., *Aquabispora*, *Ascotaiwania* (= *Neoascotaiwania*), *Bactrodesmium*, *Canalisporium* (= *Ascothailandia*), *Dematiosporium*, *Kaseifertia*, *Rhexoacrodictys*, and *Savoryella* in this family. However, the bactrodesmium-like genus, *Kaseifertia*, is related to *Pleosporales* (Réblová et al. 2020), and several recent studies have not incorporated this genus into *Savoryellaceae* (Yang et al. 2022, 2023a; Yu et al. 2023; Tian et al. 2024). Moreover, recent studies have demonstrated that *Rhexoacrodictys* clustered within *Pleurotheciaceae*, and *Ascotaiwania* and *Neoascotaiwania* are situated in different subclades of *Savoryellaceae* in phylogenetic analysis (Dayarathne et al. 2019; Yang et al. 2023a; Yu et al. 2023; Tian et al. 2024; Figure 1). Based on morphological characterisation and phylogenetic analysis, Wang et al. (2024b) introduced *Ascolacicola* as a member in *Savoryellaceae*. Consequently, in this study, we accepted eight genera, *viz*., *Aquabispora*, *Ascolacicola*, *Ascotaiwania*, *Bactrodesmium*, *Canalisporium*, *Dematiosporium*, *Neoascotaiwania*, *Savoryella* within *Savoryellaceae*.

*Aquabispora*, *Ascotaiwania*, *Ascolacicola*, *Canalisporium*, *Neoascotaiwania*, and *Savoryella* have been reported as the sexual morph, which are characterised by immersed or superficial, globose to pyriform or ellipsoidal ascomata with or without neck, 2- or 8-spored, cylindrical to clavate asci with an apical ring, and ellipsoidal, fusiform, or muriform ascospores with or without a gelatinous sheath. Except for *Aquabispora*, all these genera have cylindric-clavate asci with an apical ring, fusiform, transversely septate ascospores with larger, dark central cells and smaller and pale ends cells. Due to these similarities, they are difficult to distinguish based solely on sexual morphological characteristics (Chang et al. 1998; Ranghoo and Hyde 1998; Sri-Indrasutdhi et al. 2010; Dayarathne et al. 2019; Yang et al. 2023a; Yu et al. 2023). The asexual morph of *Savoryellaceae* is characterised by micronematous, short, or reduced conidiophores, holoblastic conidiogenous cells, and subglobose, ellipsoidal, obovoidal or pyriform, muriform, dark conidia (Dayarathne et al. 2019; Luo et al. 2019; Zhang et al. 2019; Réblová et al. 2020; Goh and Kuo 2021; Tian et al. 2024). Species of *Savoryellaceae* are widely distributed in terrestrial, freshwater, and marine habitats around the world, and are often saprophytic on the surface of decaying wood (Dayarathne et al. 2019; Réblová et al. 2020; Yang et al. 2022; Yu et al. 2023).

***Ascolacicola*** Ranghoo & K.D. Hyde

Notes: Ranghoo and Hyde (1998) established *Ascolacicola*, with *A. aquatica* as the type species. *Ascolacicola* is characterised by inconspicuous conidiophores and conidiogenous cells, broadly obovoidal or pyriform, septate, sometimes thickly banded at the septum conidia with the upper cells larger than the basal cells in asexual morph; immersed or superficial, globose to subglobose, black, uniloculate, ostiolate ascomata, periphysate neck, composed of several layers of brown cells peridium, hypha-like or filiform paraphyses, cylindrical, pedicellate asci with an apical ring, and fusiform or ellipsoidal, transversely septate, central cells larger and dark, ends cells smaller and pale ascospores in sexual morph (Ranghoo and Hyde 1998; Dayarathne et al. 2019; Wang et al. 2024b; Liu et al. 2025). Based on phylogenetic analysis and morphological characteristics, Wang et al. (2024b) accepted *Ascolacicola* in *Savoryellaceae*, and accommodates five species in this genus. Currently, six species are included in *Ascolacicola*, and only the type species lacks sequence data. In this study, we identify three new collections as *A. aquatica* based on morphological characteristics, and molecular sequences are provided.

***Ascolacicola aquatica*** Ranghoo & K.D. Hyde, Mycologia 90 (6): 1056 (1998), Figures 29, 30

**Figure 29.**
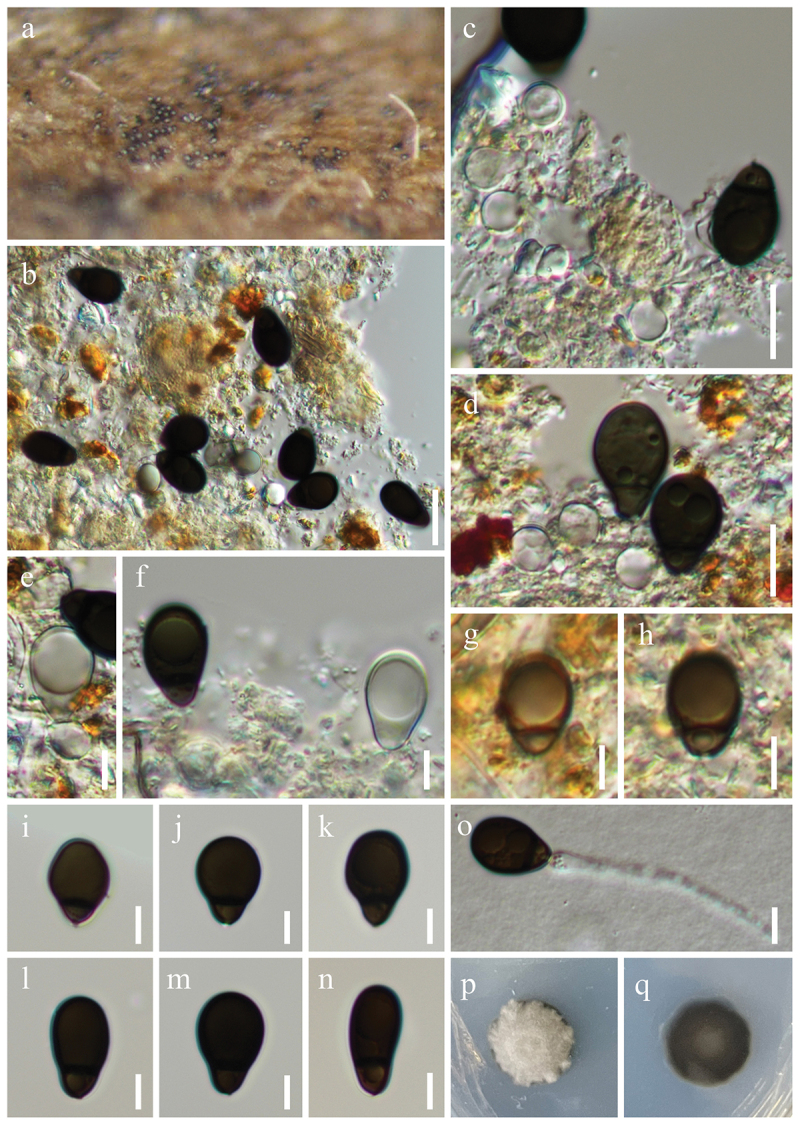
Asexual morph of *Ascolacicola aquatica* (HKAS 139448). (a) Colonies on the substratum. (b–h) Conidia in substratum. (i–n) Conidia. (o) Germinating conidium. (p, q) Colony on PDA from surface and reverse. Scale bars: b–d = 10 µm, e–o = 5 µm.

**Figure 30.**
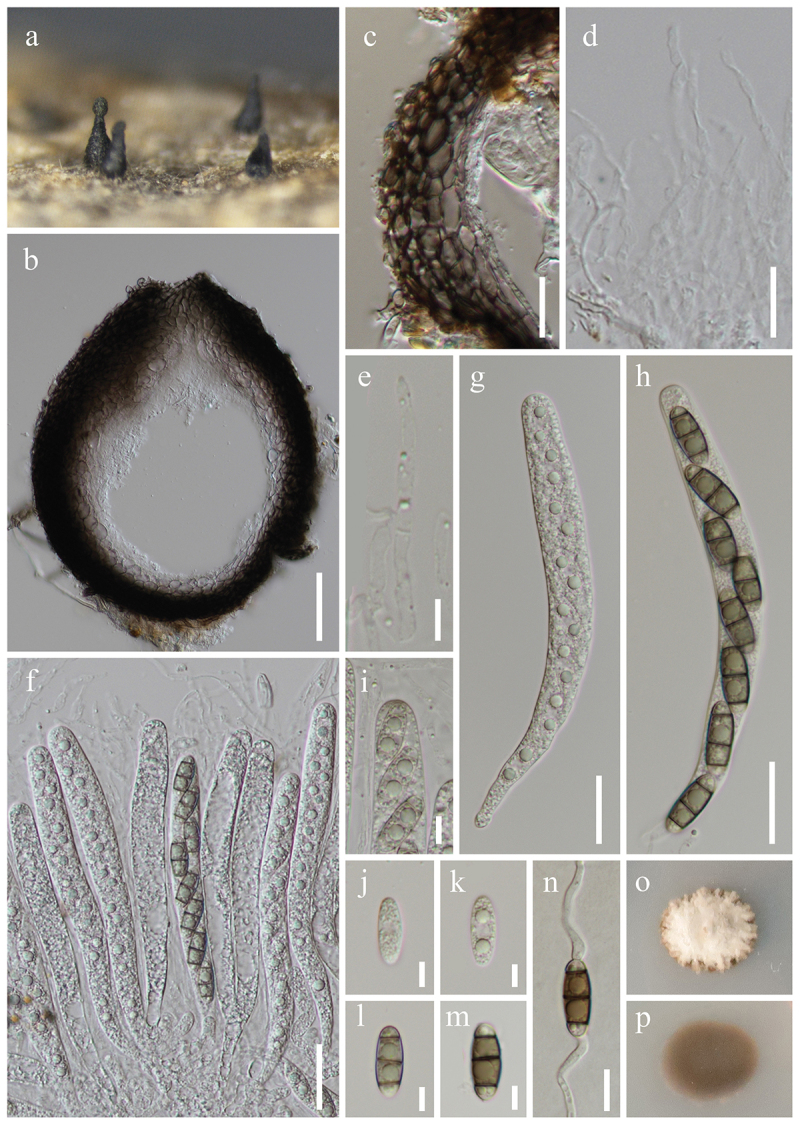
Sexual morph of *Ascolacicola aquatica* (HKAS 139431). (a) Ascomata on the substratum. (b) Vertical section of ascoma. (c) Structure of peridium. (d, e) Paraphyses. (f–h) Asci. (i) Apex of ascus. (j–m) Ascospores. (n) Germinating ascospore. (o, p) Colonies on PDA from surface and reverse. Scale bars: b = 40 µm, c, d = 15 µm, e, i–m = 5 µm, f = 25 µm, g, h = 20 µm, n = 10 µm.

*Fungal Names number*: FN446951.

*Saprobic* on submerged decaying wood. **Asexual morph**: *Colonies* on the substratum superficial, effuse, scattered or gathered, dark brown to black. *Mycelium* immersed, composed of unbranched, subhyaline to pale brown hyphae. *Conidiophores* reduced. *Conidiogenous cells* holoblastic. *Conidia* 8.7–16.1 × 5.7–11.2 µm ($\overset{ˉ}{x}$ = 12.9 × 8.3 µm, *n* = 40), acrogenous, solitary, pyriform to obovoidal, broadly rounded at the apex, straight or slightly curved, smooth-walled, uniseptate at the base, thickened at the septum, dividing the conidium into unequal cells, the upper cell is larger than the lower cell, guttulate, hyaline to brown when young, dark brown to black when mature. **Sexual morph**: *Ascomata* 125–208 µm in diam., 205–248 µm high, erect ovoidal, scattered, mostly solitary, sometimes gathered, superficial, black, coriaceous, uniloculate, ostiolate, with a short, rostrum neck. *Peridium* 8.4–40 µm thick, thickened around the ostiole, 2-layered, outer layer consisting of multi-rows of brown, thick-walled, polyhedral cells of *textura angularis*, inner layer comprising 1–2 rows of hyaline to pale brown, thin-walled, globose to subglobose cells of *textura globulosa*. *Paraphyses* 1.4–2.1 µm wide, filamentous or cylindrical, thin-walled, hyaline, sometimes branched, sparse septate, slightly tapering towards the apex. *Asci* 112–152 × 11–26 µm ($\overset{ˉ}{x}$ = 135.4 × 13.3 µm, *n* = 30), 8-spored, unitunicate, cylindrical, slightly curved, pedicellate, rounded apical. *Ascospores* 14–20 × 5.1–7.1 µm ($\overset{ˉ}{x}$ = 16.6 × 6.2 µm, *n* = 30), obliquely uniseriate, straight, fusoid to ellipsoidal, rounded at both ends, hyaline when young without septate, becoming three black septate when mature, central cells larger, olive green, end cells subhyaline, guttulate.

Culture characteristics: Conidia germinating on PDA within 48 h, with germ tubes produced from the base. Colonies on PDA reaching 10 mm in diameter after 7 weeks at room temperature. Mycelia dry, dense. Colonies on the surface of PDA, slightly protrusion, with regular edges, compaction, surface covered with a layer of grey hyphae, brown to dark green from below. Ascospores germinating on PDA within 24 h, with germ tubes produced from both ends. Colonies on PDA reaching 10 mm in diameter after 9 weeks at room temperature. Mycelia dry, dense. Colonies on the surface of PDA, slightly protrusion, with regular edges, compaction, surface covered with a layer of white hyphae, brownish-yellow, smooth from below.

Material examined: China, Yunnan Province, Wenshan Autonomous Prefecture, Guangnan County (24°11′51.30″N; 104°92′82.31″E), on unknown submerged decaying wood in a freshwater stream, 19 July 2023, Wen-Peng Wang, S-5699 (HKAS 139448), living cultures, CGMCC 3.27466 = KUNCC 23-16631; Qujing, Luoping County (25°01′27.11″N; 104°36′75.96″E), on unknown submerged decaying wood in a freshwater stream, 15 July 2023, Wen-Peng Wang, S-5839 (HKAS 139454), living culture, KUNCC 23-16834; Guangxi Autonomous Region, Hechi (24°55′70.58″N; 107°21′44.02″E), on unknown submerged decaying wood in a freshwater stream, 19 February 2024, Wen-Peng Wang, S-6452 (HKAS 139431), living culture, KUNCC 23-18634.

Notes: *Ascolacicola aquatica* was introduced by Ranghoo and Hyde (1998) from freshwater habitat, but no corresponding molecular data were provided. In this study, the new collection (HKAS 139431) is similar to *A. aquatica* in having superficial ascomata with a rostra neck, filamentous paraphyses, cylindrical, pedicellate asci, and ellipsoidal, 4-celled ascospores with similar size (14–20 × 5.1–7.1 µm vs. 12.5–16.5 × 4–7.5 µm) (Ranghoo and Hyde 1998). Therefore, we identify the new collection as *Ascolacicola aquatica* based on morphological characteristics.

In our phylogenetic tree, our new sexual strain of *Ascolacicola aquatica* (KUNCC 23-18634) and our two new asexual strains (KUNCC 23-16631 and KUNCC 23-16834) clustered with *A. mitriformis* with 96% ML/0.95 PP support. However, *A. aquatica* has ovoidal ascomata with a short, rostra neck, smaller asci (112–152 × 11–26 µm vs. 325–375 × 25–35 µm), and fusoid to ellipsoidal, 3-septate, smaller conidia (14–20 × 5.1–7.1 µm vs. 62.5–72.5 × 12.5–17.5 µm) rounded at both ends, which can be clearly distinguished from *A. mitriformis* (Ranghoo and Hyde 1998). Comparisons of the LSU sequence of the new collection (KUNCC 23-16631) to *A. aquatica* (HKAS 139431) and *A. mitriformis* (HKUCC 3706) reveal a difference of 0.12% (1/803 bp, two gaps) and 0.85% (7/820 bp, 8 gaps), respectively. Morphologically, our two new collections have uniseptate, small conidia which tally with the morphological descriptions of *A. aquatica*, and differ from *A. mitriformis*. We therefore identify our two new asexual strains (KUNCC 23-16631 and KUNCC 23-16834) as *A. aquatica*.

***Bactrodesmium*** Cooke

Notes: *Bactrodesmium* was introduced by Cooke (1883), with *B. abruptum* as the type. Based on phylogenetic analysis, Réblová et al. (2020) accepted *Bactrodesmium* in *Savoryellaceae*, and the characteristics of *Bactrodesmium* were summarised through morphological studies and 35 species were included in this genus. *Bactrodesmium* is characterised by conidiomata sporodochia, mononematous, macronematous to semi-macronematous, simple or sparsely or penicillately branched, hyaline and thin-walled conidiophores, terminal, holoblastic conidiogenous cells, and solitary, various shapes, septate conidia (Borse et al. 2019; Réblová et al. 2020). Although the phylogenetic position of this genus has been determined, most *Bactrodesmium* species do not have reliable DNA sequence data, therefore more data are needed for more comprehensive and accurate studies of this genus.

**Figure 31.**
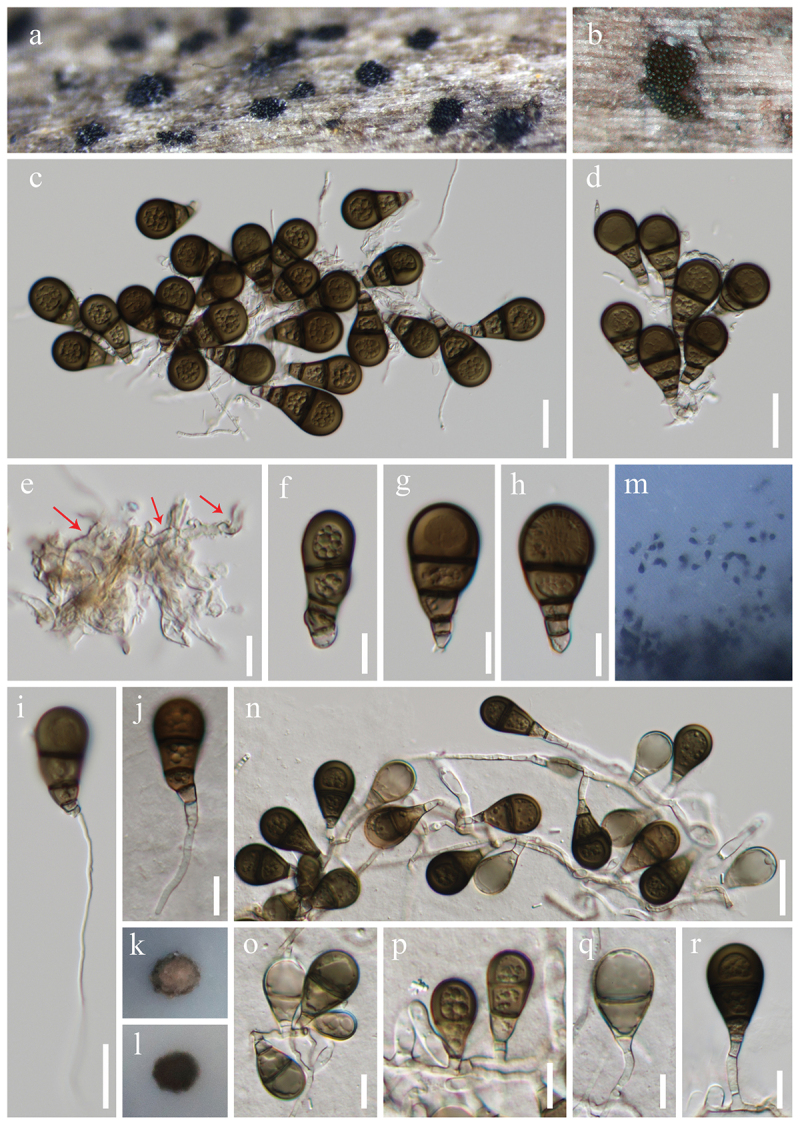
*Bactrodesmium brunneosporum* (HKAS 139444, holotype). (a, b) Colonies on the substratum. (c, d) Mount of conidia. (e) Conidiophores. (f–h) conidia. (i) Conidia with hyphae. (j) Germinating conidium. (k, l) Colony on PDA from surface and reverse. (m–r) Sporulation observed on PDA [(m) Colonies on PDA; (n–r) Conidia produced from hyphae]. Scale bars: c, d, i, n = 20 µm, e–h, j, o–r = 10 µm.

***Bactrodesmium brunneosporum*** W.P. Wang & Z.L. Luo, sp. nov., Figure 31

*Fungal Names number*: FN572357.

*Etymology*: Referring to the brown conidia of this fungus.

*Holotype*: HKAS 139444.

3.2.1.

*Saprobic* on submerged decaying wood. **Asexual morph**: *Colonies* superficial, conidiomata sporodochia, dark brown to black, glistening. *Mycelium* immersed, composed of branched, aseptate, subhyaline, smooth hyphae. *Conidiophores* 2.3–2.6 µm wide, reduced or micronematous to semi-macronematous, arising from basal hyphae, aseptate, pale brown, unbranched. *Conidiogenous cells* terminal, integrated, monoblastic. *Conidia* 23–38 × 11–18 µm ($\overset{ˉ}{x}$ = 30 × 15 µm, *n* = 40), acrogenous, solitary, mostly produced from the ends of hyphae, pyriform to clavate, broad rounded apical, truncate base, (2–) 3 (−4) septate, thickening at the septum, smooth-walled, brown, guttulate. **Sexual morph**: Undetermined.

Culture characteristics: Conidia germinating on PDA within 36 h, with germ tubes produced from the base. Colonies on PDA reaching 8 mm in diameter after 5 weeks at room temperature. Mycelia dry, dense. Colonies semi-immersed in PDA, with regular edges, surface rough, brown, dark brown from below. *Conidiophores* reduced. *Conidiogenous cells* monoblastic, integrated, cylindrical, hyaline. *Conidia* 23–37 × 12–16 µm ($\overset{ˉ}{x}$ = 30 × 13.9 µm, *n* = 30), solitary, produced from hyphae, lateral to terminal, pyriform to clavate, broad rounded apical, 2–3-septate, thickening at the upper septum, smooth-walled, brown, guttulate.

Material examined: China, Yunnan Province, Nujiang Autonomous Prefecture, Gongshan County, Dulongjiang River (27°77′48.01″N; 98°34′09.77″E), on unknown submerged decaying wood, 24 April 2024, Ying Wang, S-6687 (HKAS 139444, holotype), ex-type cultures, CGMCC 3.28463 = KUNCC 24-19067.

Notes: Phylogenetic analysis showed that *Bactrodesmium brunneosporum* formed a distinct basal lineage to *B. diversum*, *B. leptopus*, *B. pallidum*, and *B. spilomeum* with 96% ML/1.00 PP support (Figure 1). However, conidiogenous cells of *B. brunneosporum* are monoblastic, but *B. diversum* has polyblastic conidiogenous cells (Table 1). Furthermore, *B. brunneosporum* has shorter and broader conidia with less septate (Table 1), which can be distinguished from *B. leptopus*, *B. pallidum*, and *B. spilomeum*. Comparisons of the ITS sequences between *B. brunneosporum* and *B. abruptum* (CBS144404), *B. diversum* (CBS 144081), *B. leptopus* (CBS 144542), *B. obovatum* (CBS 145350), and *B. pallidum* (CBS 145349) showed a difference of 12.53% (62/495 bp, 28 gaps), 13.58% (66/486 bp, 24 gaps), 8.25% (40/485 bp, 22 gaps), 11.74% (58/494 bp, 27 gaps), and 14.29% (73/511 bp, 21 gaps), which strongly support our isolate as a new species.

**Table 1.** Comparative morphology of *Bactrodesmium* species in the phylogenetic tree.

Species	Conidiogenous cells	Conidia	
		Size (µm)	Number of septa
*Bactrodesmium abruptum*	Terminal, monoblastic	(36.5–) 42–65.5 (−70) × (12.5–) 14–18 (−19)	(3–) 4–6 (−7)
*B. brunneosporum*	Terminal, monoblastic	23–38 × 11–18	(2–) 3 (−4)
*B. diversum*	Terminal, polyblastic	(27–) 30–48 (−52.5) × (14–) 15–19.5 (−20.5)	3–5 (−6)
*B. leptopus*	Terminal, monoblastic	(21–) 24–42 (−44) × 11.5–15.5	3–5
*B. obovatum*	Terminal, monoblastic	(27.5–) 35–46 (−48) × 15.5–20	(3–) 4–5
*B. pallidum*	Terminal, monoblastic	29–54 (−57) × 9–13	(4–) 5–6
*B. spilomeum*	Terminal, monoblastic	24–43 × 8.5–11	3–5

***Canalisporium*** Nawawi & Kuthub.

Notes: *Canalisporium* was established by Nawawi and Kuthubutheen (1989), with *C. caribense* as the type species. Currently, 24 species are accepted in *Canalisporium* (including a sexual species, *C. grenadoideum*), and these species are commonly found on wood collected from pantropical freshwater habitats (Goh and Kuo 2021). *Canalisporium* is characterised by sporodochia conidiomata composed of dark, complanate, muriform conidia with clear longitudinal and transverse separation in asexual morph (Tibpromma et al. 2018; Hyde et al. 2019, 2020a; Goh and Kuo 2021; Yu et al. 2023); 8-spored, cylindrical, pedicellate asci with apical ring, ovoidal to fusiform, slightly curved, 3-septate ascospores with dark middle cells, larger than ends cells (Sri-Indrasutdhi et al. 2010).

***Canalisporium kenyense*** Goh, W.H. Ho & K.D. Hyde, Can. J. Bot. 76 (1): 148 (1998), Figure 32

**Figure 32.**
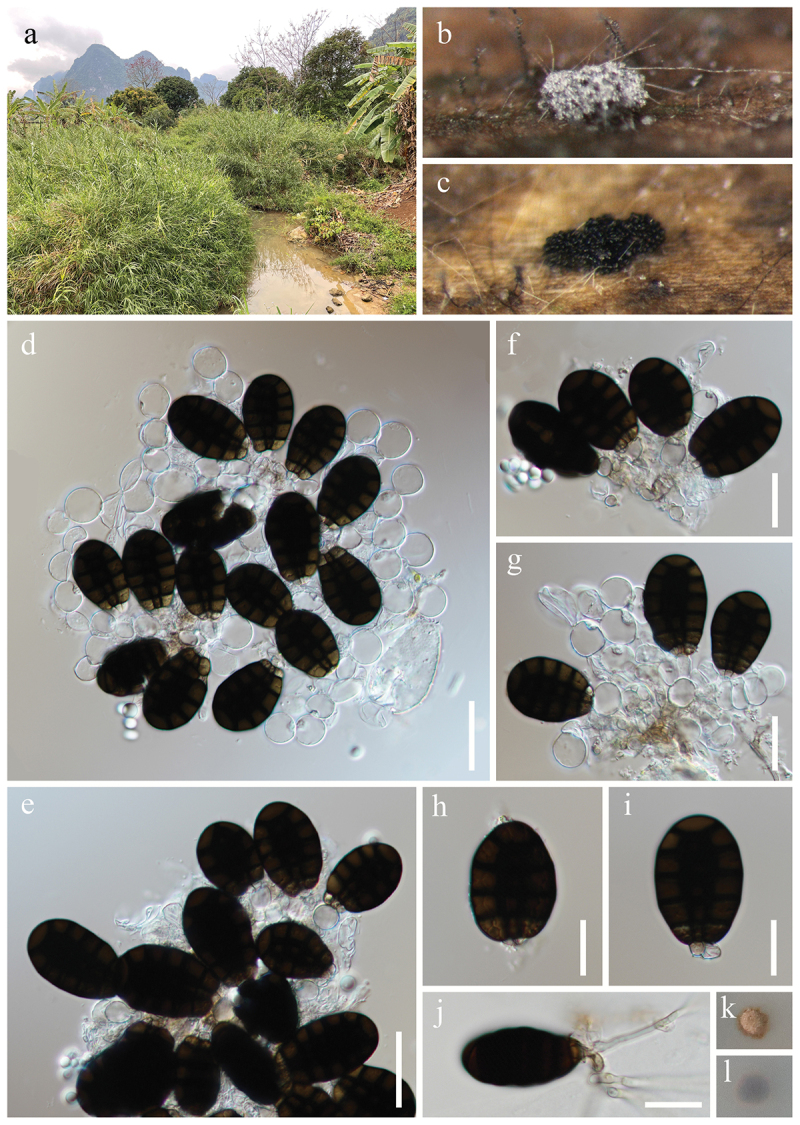
*Canalisporium kenyense* (HKAS 139447). (a) Freshwater habitat. (b, c) Colonies on the substratum. (d–g) Conidiophores and conidia. (h, i) Conidia. (j) Germinating conidium. (k, l) Colony on PDA from surface and reverse. Scale bars: d, e = 30 µm, f, g = 20 µm, h–j = 15 µm.

*Fungal Names number*: FN446332.

Material examined: China, Yunnan Province, Qujing, Luoping County (24°76′57.73″N; 104°49′72.38″E), on unknown submerged decaying wood in a freshwater river, 16 July 2023, Xing-Ya Zeng, S-5948 (HKAS 139451), living culture, KUNCC 23-17554; Guangxi Autonomous Region, Chongzuo (22°52′67.00″N; 107°01′50.18″E), on unknown submerged decaying wood in a freshwater stream, 27 February 2023, Wen-Peng Wang, S-6441 (HKAS 139447), living culture, KUNCC 24-18380.

***Dematiosporium*** Z.L. Luo, K.D. Hyde & H.Y. Su

Notes: *Dematiosporium* was introduced by Luo et al. (2019), with *D. aquaticum* as the type. Only two asexual species *D. aquaticum* and *D. bambusicola* are accepted in this genus, and both were recovered from freshwater habitats (Luo et al. 2019; Réblová et al. 2020; Yu et al. 2023). *Dematiosporium* is characterised by reduced to conidiogenous cells or micronematous, mononematous, cylindrical, unbranched, aseptate conidiophores, monoblastic, terminal, determinate conidiogenous cells, and dictyospores, ellipsoidal or globose to subglobose conidia.

**Figure 33.**
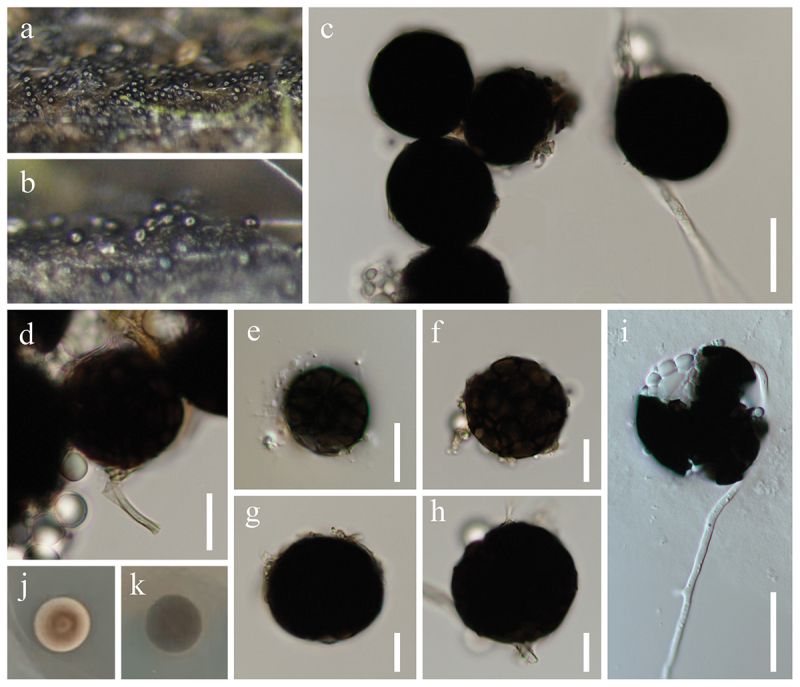
*Dematiosporium globosum* (HKAS 139470, holotype). (a, b) colonies on the substratum. (c, d) hyphae with conidia. (e–h) conidia. (i) Germinating conidium. (j, k) colony on PDA from surface and reverse. Scale bars: c, d, i = 20 µm, e–h = 10 µm.

***Dematiosporium globosum*** W.P. Wang & Z.L. Luo, sp. nov., Figure 33

*Fungal Names number*: FN572358.

*Etymology*: Referring to the globose conidia of this fungus.

*Holotype*: HKAS 139470.

*Saprobic* on submerged decaying wood. **Asexual morph**: *Colonies* on the substratum superficial, effuse, scattered, gathered, dark brown to black, glistening. *Mycelium* mostly immersed, partly superficial, composed of branched, septate, subhyaline to pale brown hyphae. *Conidiophores* reduced. *Conidiogenous cells* not observed. *Conidia* (17–) 23–36 µm ($\overset{ˉ}{x}$ = 29.2 µm, *n* = 40) in diam., acrogenous, directly grows at the ends of hyphae, solitary, globose to subglobose, dark brown to black, irregular dictyosporous, thickened, slightly constricted at the septum, thin-walled. **Sexual morph**: Undetermined.

Culture characteristics: Conidia germinating on PDA within 24 h, with germ tubes produced from the surface. Colonies on PDA reaching 12 mm in diameter after 4 weeks at room temperature. Mycelium dry, dense. Colonies on the surface of PDA, slightly protrusion, with regular edges, brown, with pale brown edges, fluffy, dark brown, smooth from below.

Material examined: China, Yunnan Province, Honghe Autonomous Prefecture, Mile (24°42′69.75″N; 103°48′34.68″E), on unknown submerged decaying wood in a freshwater stream, 14 July 2023, Zheng-Quan Zhang, S-5355 (HKAS 139470, holotype), ex-type cultures, CGMCC 3.27464 = KUNCC 23-15597.

Notes: In the phylogenetic analysis, *Dematiosporium globosum* is sister to *D. aquaticum* with 92% ML/1.00 PP support (Figure 1). Comparisons of the ITS, *tef*1-α, and *rpb*2 sequences between *D. globosum* and *D. aquaticum* (CBS 144793) reveal a difference of 9.69% (57/588 bp, 33 gaps), 4.9% (35/715 bp), and 6.49% (66/1,017 bp, one gap), respectively. Conidia of *D. globosum* grows directly at the ends of hyphae, thickened and constricted at the septum, different from *D. aquaticum* (Luo et al. 2019). We therefore recognise *D. globosum* as a new species following the guidelines of Jeewon and Hyde (2016).

***Savoryella*** E.B.G. Jones & R.A. Eaton

Notes: *Savoryella* was introduced by Jones and Eaton (1969) and typified by *S. lignicola*. The sexual morph of *Savoryella* is characterised by immersed to superficial, globose to subglobose or ellipsoidal ascomata with a short neck, 2- or 8-spored, clavate to cylindrical asci with a nonamyloid apical ring, and ellipsoidal to fusiform, 3-septate, brown middle cells and paler end cells ascospores (Dayarathne et al. 2019; Zhang et al. 2019; Yu et al. 2023). Five asexual species, *viz*., *S. chiangraiensis*, *S. claviformis*, *S. cocois*, *S. nypae*, and *S. sarushimana*, are accepted in this genus, which are characterised by micronematous, mononematous or inconspicuous conidiophores, holoblastic, determinate, integrated, terminal, cylindrical conidiogenous cells, and pyriform to obovoidal or fusiform, septate conidia (Zhang et al. 2019; Tian et al. 2024; Xu et al. 2024b). Currently, a total of 17 species are accepted in this genus, including five marine species, ten freshwater species, and two species isolated from decaying leaves (Dayarathne et al. 2019; Zhang et al. 2019; Yu et al. 2023; Tian et al. 2024).

***Savoryella bambusicola*** X.D. Yu, S.N. Zhang & Jian K. Liu, J. Fungi 9(5, no. 571): 571 (2023)

*Fungal Names number*: FN847554.

Material examined: China, Yunnan Province, Qujing, Luoping County, Duoyihe River (24°76′57.73″N; 104°49′73.28″E), on unknown submerged decaying wood, 16 July 2023, Wen-Peng Wang, S-5849 (HKAS 139464), living culture, KUNCC 23-16839; Honghe Autonomous Prefecture, Mile, Yubu reservoir (24°47′35.66″N; 103°49′92.39″E), on unknown submerged decaying wood, 14 July 2023, Si-Qi Chen, S-5878 (HKAS 139456), living culture, KUNCC 23-16880; Mile (24°42′69.75″N; 103°48′34.68″E), on unknown submerged decaying wood in a freshwater stream, 14 July 2023, Si-Qi Chen, S-5497 (HKAS 139472), living culture, KUNCC 24-18362; Guangxi Autonomous Region, Nanning (22°48′48″N; 108°13′32″E), on unknown submerged decaying wood in a freshwater river, 17 November 2023, Qiu-Xia Yang, S-5974 (HKAS 139427), living culture, KUNCC 23-17686.

***Savoryella claviformis*** R.J. Xu, S. Boonmee, K.D. Hyde & Q. Zhao, Stud. Fung. 9 (e009): 2 (2024), Figure 34

**Figure 34.**
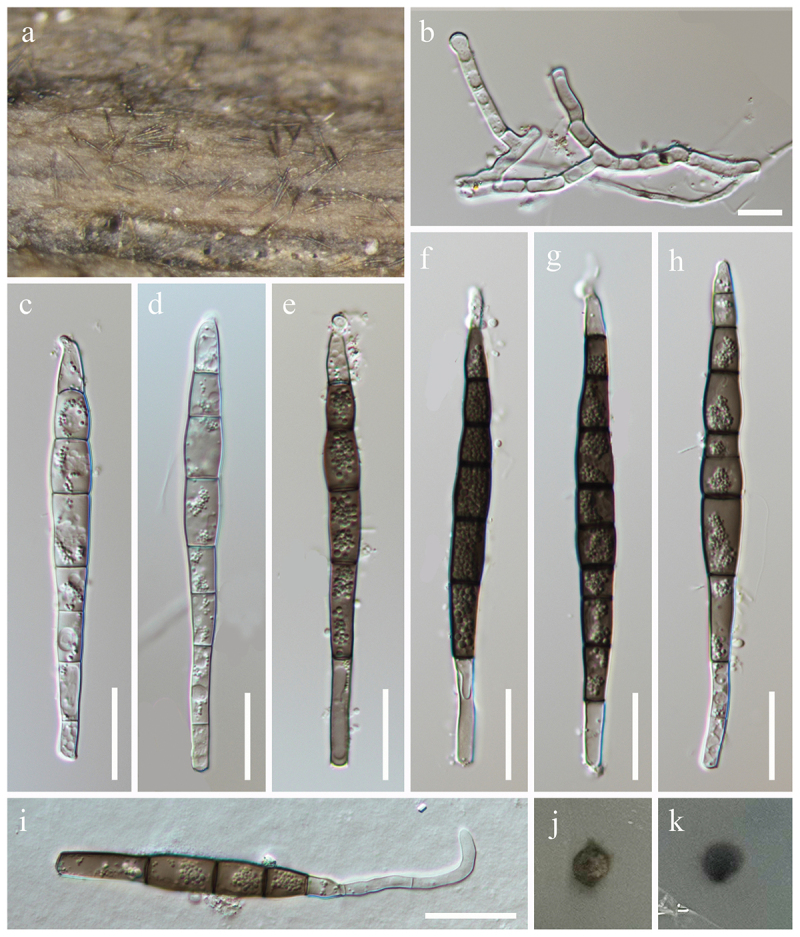
*Savoryella claviformis* (HKAS 139465). (a) Colonies on the substratum. (b) Conidiophores. (c–h) Conidia. (i) Germinating conidium. (j, k) Colonies on PDA from surface and reverse. Scale bars: b = 10 µm, c–i = 20 µm.

*Fungal Names number*: FN853230.

*Saprobic* on submerged decaying wood. **Asexual morph**: *Colonies* superficial, effuse, scattered strips, brown. *Mycelium* mostly immersed, composed of unbranched, septate, smooth, subhyaline hyphae. *Conidiophores* 26–40 (−60) × 3.1–3.5 µm ($\overset{ˉ}{x}$ = 36.3 × 3.3 µm, *n* = 10), micronematous to semi-macronematous, mononematous, cylindrical, straight or slightly flexuous, branched or unbranched, septate, subhyaline to hyaline, smooth, thin-walled. *Conidiogenous cells* monoblastic, integrated, terminal, determinate, hyaline, cylindrical. *Conidia* 70–116 × 6.2–9.9 µm ($\overset{ˉ}{x}$ = 95.1 × 8.1 µm, *n* = 20), acrogenous, solitary, long fusiform, straight, 5–9-septate, sometimes slightly constricted at the septum, central cells larger, subhyaline when young, brown to dark brown when mature, paler at both ends, tapering towards the apex, small guttulate, with a long, lighter, cylindrical base connect to conidiogenous cells. **Sexual morph**: Undetermined.

Culture characteristics: Conidia germinating on PDA within 48 h, and germ tubes produced from the base. Colonies on PDA reaching 6 mm in diameter after 6 weeks at room temperature. Mycelia dry, dense. Colonies semi-immersed in the PDA, slightly protrusion, surface rough, with irregular edges, dark green, compaction.

Material examined: China, Yunnan Province, Honghe Autonomous Prefecture, Mile, Yubu reservoir (24°47′35.66″N; 103°49′92.39″E), on unknown submerged decaying wood, 14 July 2023, Si-Qi Chen, S-5517 (HKAS 139465), living culture, KUNCC 24-17684.

Notes: Phylogenetic analysis showed that our new collection clustered with *Savoryella claviformis* (KUNCC 10408 and KUNCC 10495) with 100% and 1.00 PP support (Figure 1). Our new collection fits well with the description of *S. claviformis* in having semi-micronematous, mononematous, cylindrical conidiophores and fusiform, multiseptate conidia (Xu et al. 2024b). We therefore identify our new collection as *Savoryella claviformis*.

**Figure 35.**
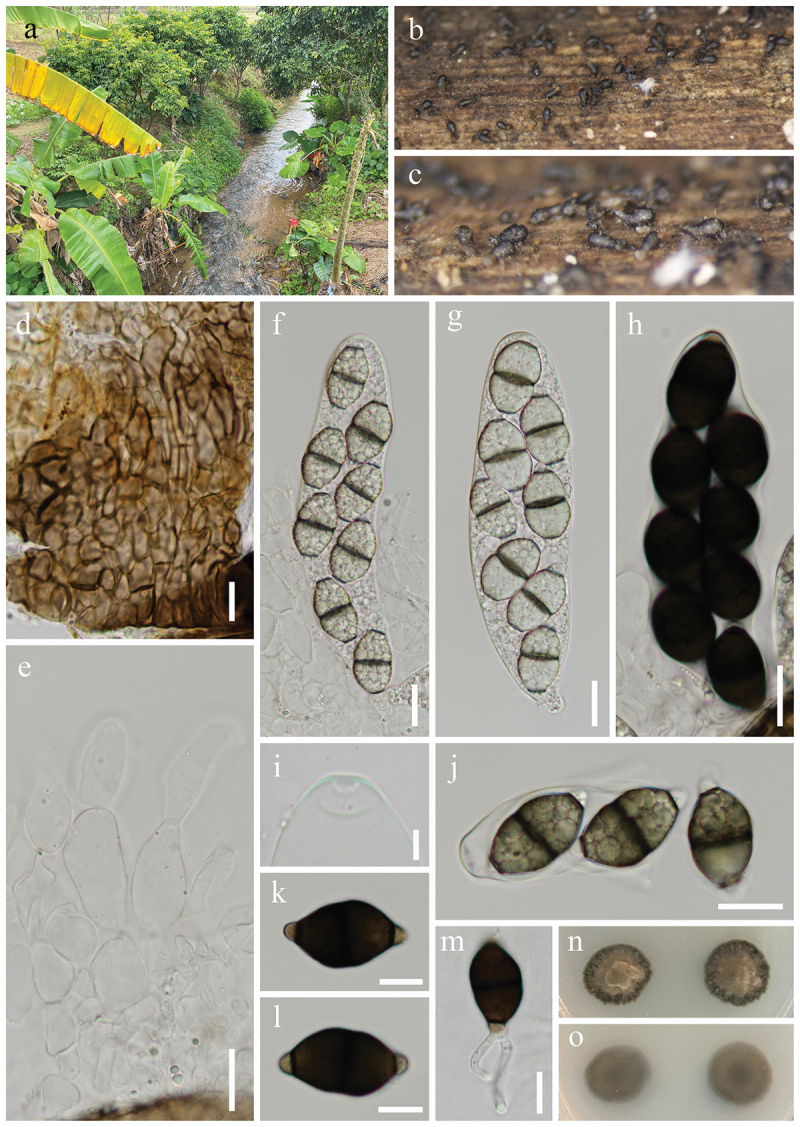
*Savoryella guangxiensis* (HKAS 139457, holotype). (a) Freshwater habitat. (b, c) Ascomata on the substratum. (d) Peridium. (e) Paraphyses. (f–h) Asci. (i) Apex of ascus. (j–l) Ascospores. (m) Germinating ascospore. (n, o) Colonies on PDA from surface and reverse. Scale bars: d, k–m = 10 µm, e–h, j = 15 µm, i = 5 µm.

***Savoryella guangxiensis*** W.P. Wang & Z.L. Luo, sp. nov., Figure 35

*Fungal Names number*: FN572359.

*Etymology*: Referring to the Guangxi Autonomous Region, China, where the species was collected.

*Holotype*: HKAS 139457.

*Saprobic* on submerged decaying wood. **Asexual morph**: Undetermined. **Sexual morph**: *Ascomata* superficial, scattered, black, coriaceous, uniloculate, laterally ostiolate, ellipsoidal or long ovoidal, lying horizontal to the host surface, with a short neck. *Peridium* thin, composed of brown, irregular-shaped cells of *textura epidermoidea* in surface view. *Paraphyses* 6.5–13 µm wide, thin-walled, hyaline, branched, septate, obviously constricted at the septum, composed of enormous ellipsoidal to subglobose to oval cells. *Asci* 74–153 × 24–36 µm ($\overset{ˉ}{x}$ = 110.8 × 30.6 µm, *n* = 20), 8-spored, unitunicate, clavate, slightly curved, short pedicellate, broad rounded at the apex, thin-walled, with an apical pore. *Ascospores* 24–30 × 12–17 µm ($\overset{ˉ}{x}$ = 27.4 × 14.6 µm, *n* = 30), biseriate inclination, straight or slightly curved, asymmetrical ellipsoidal, central cells are significantly larger than ends cells, tapering to the both ends, guttulate, three black septate, thickened and constricted at the central septum, hyaline when young, ends cells are pale brown and central cells are dark brown when mature.

Culture characteristics: Ascospores germinating on PDA within 48 h, and germ tubes produced from one end. Colonies on PDA reaching 10 mm in diameter after 9 weeks at room temperature. Mycelia dry, dense. Colonies on the surface of PDA, compaction, regular edges, protrusion, brown with dark green edges, dark green to brown, smooth from below.

Material examined: China, Guangxi Autonomous Region, Qinzhou (22°22′13.17″N; 109°43′36.91″E), on unknown submerged decaying wood in a freshwater stream, 25 February 2024, Fa-Li Li, S-6451 (HKAS 139457, holotype), ex-type culture, KUNCC 24-18386.

Notes: Phylogenetic analysis showed that *Savoryella guangxiensis* clustered with *S. aquatica* and *S. yunnanensis* with 99% ML/0.93 PP support (Figure 1). The ITS sequence between *S. guangxiensis* (KUNCC 24-18386) and *S. aquatica* (SS 03801) reveals a difference of 1.58% (7/443 bp), and there are no ITS sequences in GenBank for *S. yunnanensis*. *Savoryella guangxiensis* fits well with the generic concept of *Savoryella* and similar to *S. aquatica* and *S. yunnanensis* in having ellipsoidal, thin-walled ascomata with a laterally ostiolate, clavate asci with a short pedicellate, and ascospores with central cells significantly larger than end cells (Dayarathne et al. 2019). However, *S. guangxiensis* has shorter ascospores (24–30 µm vs. 29–38 µm) with black, thickened septum, which are different from *S. aquatica*. *Savoryella guangxiensis* differs from *S. yunnanensis* in having branched, wider range paraphyses (6.5–13 µm vs. 8 µm) composed of enormous ellipsoidal to subglobose to oval cells, broad rounded apical asci with an apical pore, and asymmetrical ellipsoidal ascospores tapering to both ends (Dayarathne et al. 2019). We therefore identify *S. guangxiensis* as a new species.

***Savoryella verrucosa*** Minoura & Muroi, Trans. Mycol. Soc. Jap. 19: 132 (1978), Figure 36

**Figure 36.**
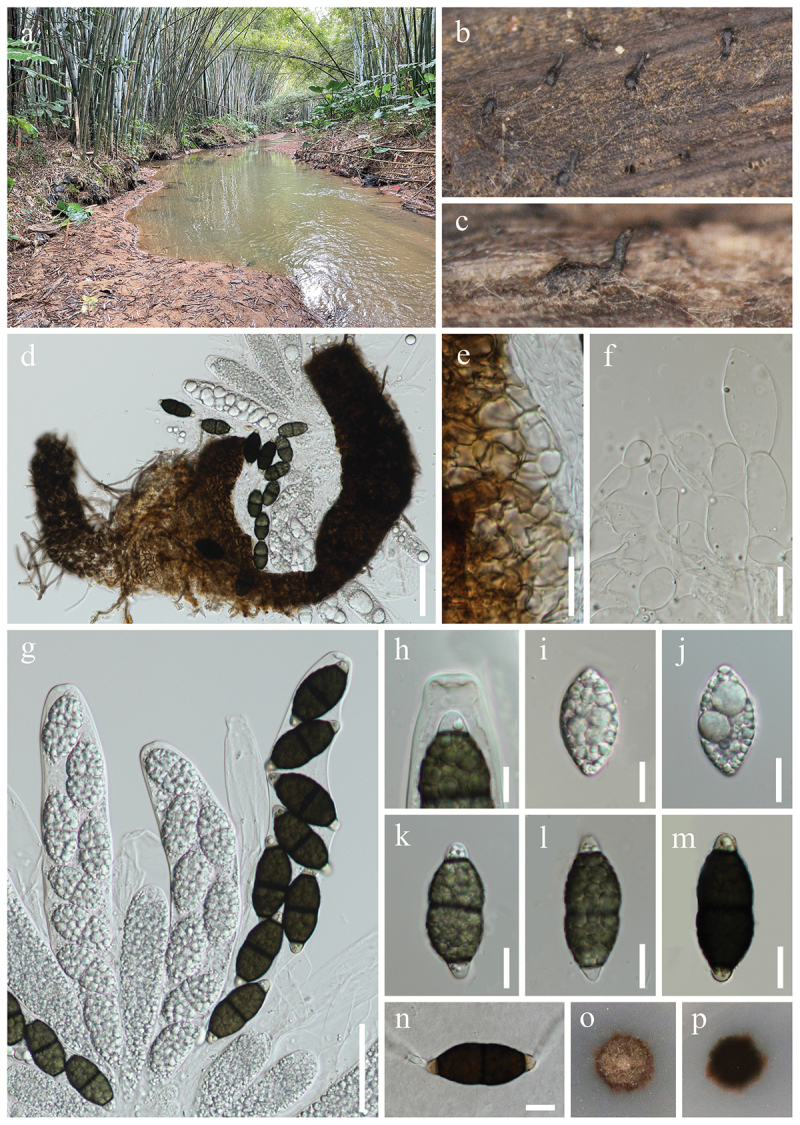
*Savoryella verrucosa* (HKAS 139445). (a) Freshwater habitat. (b, c) Ascomata on the substratum. (d) Section of ascoma. (e) Peridium. (f) Paraphyses. (g) Asci. (h) Apex of ascus. (i–m) Ascospores. (n) Germinating ascospore. (o, p) Colony on PDA from surface and reverse. Scale bars: d = 50 µm, e, f = 15 µm, g = 40 µm, h = 5 µm, i–n = 10 µm.

*Fungal Names number*: FN323112.

*Saprobic* on submerged decaying wood. **Asexual morph**: Undetermined. **Sexual morph**: *Ascomata* 295–337 µm long, 222–242 µm high, semi-immersed to superficial, scattered, solitary, black, coriaceous, uniloculate, laterally ostiolate, ellipsoidal to pyriform, lying horizontal to the host surface, with a cylindrical, bending upwards neck. *Peridium* thin, composed of hyaline to brown, irregular-shaped cells of *textura epidermoidea* in surface view. *Paraphyses* thin-walled, hyaline, branched, septate, obviously constricted at the septum, composed of 6.5–13 µm wide, enormous ellipsoidal to subglobose to oval or irregular-shaped cells. *Asci* 169–234 × 23–40 µm ($\overset{ˉ}{x}$ = 201.7 × 31 µm, *n* = 20), 8-spored, unitunicate, clavate to cylindrical, straight or slightly curved, short pedicellate, broad rounded, and thickened at the apex, thin-walled, with an apical ring. *Ascospores* 38–40 × 13–17 µm ($\overset{ˉ}{x}$ = 37.8 × 14.7 µm, *n* = 30), uniseriate inclination or biseriate perpendicular, straight or slightly curved, ellipsoidal to fusiform, central cells are significantly larger than ends cells, taping to the both ends, guttulate, three black septate, thickened and constricted at the central septum, hyaline, smooth-walled when young, ends cells are pale brown and central cells are olive green to dark brown, verrucose when mature.

Culture characteristics: Ascospores germinating on PDA within 48 h, with germ tubes produced from both ends. Colonies on PDA reaching 15 mm in diameter after 8 weeks at room temperature. Mycelia dry, dense. Colonies semi-immersed in PDA, slightly protrusion in central, with irregular edges, brown, brown to dark brown from below.

Material examined: China, Guangxi Autonomous Region, Yulin (22°27′76.41″N; 109°78′08.08″E), on unknown submerged decaying wood in a freshwater stream, 25 February 2024, Wen-Peng Wang, S-6393 (HKAS 139445), living culture, KUNCC 24-19059.

Notes: In the phylogenetic analysis, the new collection (KUNCC 24-19059) clustered with *Savoryella verrucosa* (SS 03331) with 92% ML support (Figure 1). Our new collection is similar to *S. verrucosa* in having ascomata that lie horizontally to the host surface with a cylindrical, bending upwards neck, clavate to cylindrical asci with short pedicellate and apical ring, and ellipsoidal, 3-septate conidia with verrucose walls (Ho et al. 1997). Comparison of the ITS sequence of the new collection and *S. verrucosa* (SS 03331) reveals a 99.57% (462/464 bp) similarity. We therefore identify our new collection as *S. verrucosa*, and it is a new geographical record in China.

### 3.3. *Biogeography of freshwater* Savoryellomycetidae

**Figure 37.**
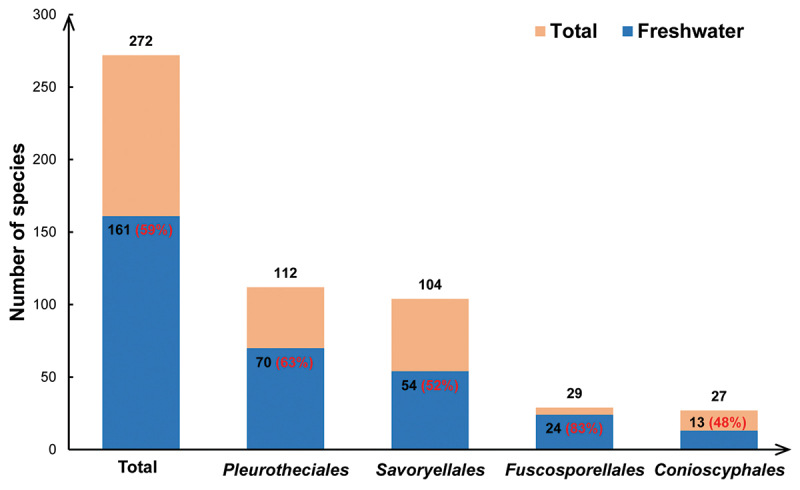
The number of *Savoryellomycetidae* species collected from freshwater habitats and totals.

By integrating the data from this study and previous research, as of 31 December 2024, a total of 272 species are currently recognised in *Savoryellomycetidae*. These species are distributed in 31 genera within *Conioscyphaceae* (1 genus, 27 species), *Fuscosporellaceae* (6 genera, 29 species), *Pleurothecieceae* (16 genera, 112 species), and *Savoryellaceae* (8 genera, 104 species). Among these, 161 (59%) species have been recorded in freshwater habitats (Figure 37). The numbers of these freshwater species corresponding to each family are as follows: *Conioscyphaceae* (1 genus, 13 species), *Fuscosporellaceae* (6 genera, 24 species), *Pleurothecieceae* (10 genera, 70 species), and *Savoryellaceae* (8 genera, 54 species). The families with the highest and lowest proportions of freshwater species are *Fuscosporellaceae* (83%) and *Conioscyphaceae* (48%), respectively (Figure 37). The genus with the largest number of freshwater species is *Pleurotheciella* (24 species). Nine genera, *viz*., *Helicoascotaiwania* (2 species), *Obliquifusoideum* (2 species), *Sterigmatobotrys* (3 species), *Fuscosporella* (4 species), *Mucispora* (5 species), *Pseudoascotaiwania* (1 species), *Plagiascoma* (1 species), *Aquabispora* (3 species), and *Dematiosporium* (3 species), are all genera with species that have been collected from freshwater habitats (Table S2).

**Figure 38.**
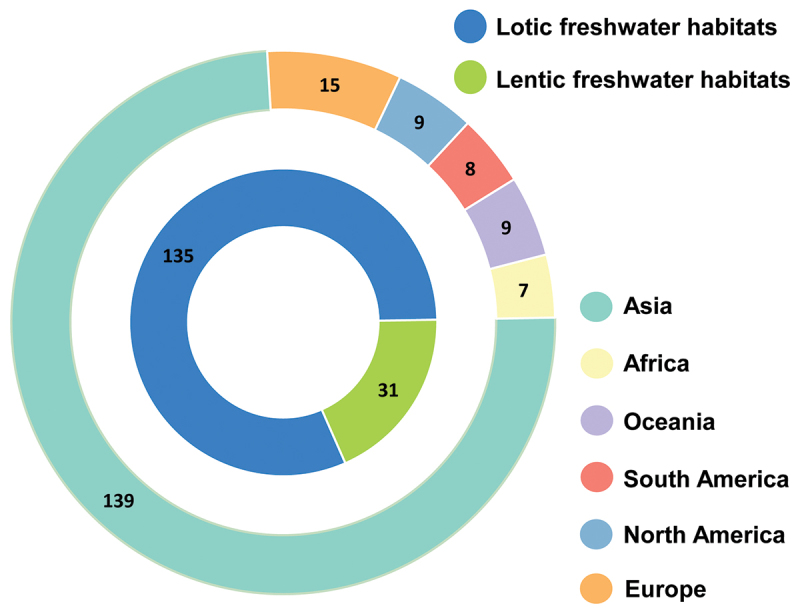
The number of freshwater *Savoryellomycetidae* distributed across different freshwater environments and continents. The outer ring signifies the number of species collection sites on different continents. The inner ring indicates the number of species collected from different types of freshwater habitats.

161 freshwater species of *Savoryellomycetidae* have been gathered from 20 countries across Africa, Asia, Europe, Oceania, North America, and South America. The continent with the highest incidence of freshwater *Savoryellomycetidae* species is Asia, where a total of 139 species have been recorded, followed by Europe (15), North America (9), Oceania (9), South America (8), and Africa (7) (Figure 38). We categorise the freshwater environment where freshwater *Savoryellomycetidae* species are collected into lotic (river and stream) and lentic (lake, reservoir, swamp, and wetland) freshwater habitats. A total of 147 freshwater species of *Savoryellomycetidae* have specific collection information. Among them, 135 species were collected from lotic freshwater habitats, 31 species were collected from lentic freshwater habitats, and 19 species were collected from both lotic and lentic freshwater habitats. The results demonstrate that more species were collected in lotic freshwater habitats than from lentic freshwater habitats (Figure 38). Additionally, in the freshwater environment, the nutrient mode of *Savoryellomycetidae* species is relatively straightforward. Saprophytism is the only known nutrient mode, and the host is mainly wood. Only one species (*Savoryella nypae*) was found on leaf (Table S2).

## 4. Discussion

This study provides the most comprehensive and systematic update on *Savoryellomycetidae* to date. Herein, we present an updated phylogeny of this subclass, with 189 species and 349 strains, which can serve as a reference for researchers studying related taxa. Based on the phylogenetic analysis, we transfer *Parafuscosporella atricolor* and *P. hunanensis* to *Vanakripa*, synonymise *Saprodesmium* with *Rhexoacrodictys* and introduce *R. dematiospora* comb. nov., and some cases of synonymy have been addressed, including *Dematipyriforma globispora* and *D. americana* under *D. aquatica*, *D. nilotica* under *D. aquilariae*, *Phaeoisaria fasciculata* under *P. loranthacearum*, and *Pleurotheciella verrucosa* under *P. nilotica*. Furthermore, our study reports ten new species and provides new strains and descriptions for a part of known species, enhancing our knowledge on the biodiversity of *Savoryellomycetidae*. Drawing on our data and previous research, we have conducted a biogeographic study of freshwater *Savoryellomycetidae* on a global scale, utilising data and images to visually reflect their distribution.

In our statistics, Asia, particularly China and Thailand, emerges as the region with the highest reported concentration of *Savoryellomycetidae* fungi, including the ten new species reported in this study all originate from China. This extreme sampling bias poses a significant challenge, making it difficult to accurately gauge the global biodiversity level and distribution pattern of this subclass. Objectively, our findings merely depict the distribution pattern and species diversity of *Savoryellomycetidae* fungi in Asian freshwater habitats. In contrast, the current distribution and biodiversity across other continents remain largely shrouded in mystery. This largely limits our understanding of the biodiversity level of this taxon, and also obscures the true pattern of its distribution in freshwater habitats worldwide. Moreover, *Savoryellomycetidae* species are also found in terrestrial environments, with complex life histories. Therefore, more comprehensive biogeography studies can be carried out in the future.

Additionally, unresolved taxonomic issues remain, particularly within the family *Pleurotheciaceae*. Arzanlou et al. (2007) accepted *Pleurothecium obovoideum* in *Pleurothecium*, subsequent studies on *Pleurotheciaceae* have consistently demonstrated that *P. obovoideum* could not be accommodated in *Pleurothecium*, and the morphological characteristics of *P. obovoideum* do not fit into the generic concept of *Pleurothecium* (Dong et al. 2021; Bao et al. 2022; Jayawardena et al. 2022; Wang et al. 2024a). Recently, He et al. (2024) transferred *P. obovoideum* to *Neomonodictys* based on phylogenetic evidence. Despite this transfer, *P. obovoideum* exhibits significant morphological differences from *Neomonodictys*. *Pleurothecium obovoideum* has reduced or macronematous, mononematous, unbranched, septate conidiophores and aseptate, ellipsoidal to obovate conidia in short chins (Arzanlou et al. 2007). In contrast, *Neomonodictys* is characterised by reduced conidiophores, muriform, solitary, ellipsoidal to obovoid or subglobose to globose, septate conidia (Hyde et al. 2020a; Huang et al. 2022). Given these distinct morphological discrepancies, it remains uncertain whether the inclusion of *P. obovoideum* in *Neomonodictys* based solely on phylogenetic analysis is appropriate. Further integrative studies, combining morphological, molecular, and ecological data, are needed to clarify its taxonomic placement.

In this study, we included four *Monotosporella* species (*M. clavata*, *M. erecta*, *M. palmicola*, and *M. sphaerica*) and *Phragmocephala stemphylioides* in *Pleurotheciaceae*. *Monotosporella clavata*, *M. palmicola*, and *M. sphaerica* were introduced by Yanna and Hyde (2002), but *M. sphaerica* lacks molecular data and exhibits considerable morphological differences. Phylogenetic analysis showed that *M. setosa* consistently clusters with *Rhexoacrodictys erecta*, but the strain of *M. setosa* (HKUCC 3713) lacks a morphological description (Bao et al. 2022; Jayawardena et al. 2022). Therefore, the genus *Monotosporella* requires a re-examination of its morphology to determine the species it contains as well as its accurate taxonomic status. Réblová et al. (2016) provided the ITS and LSU sequences of *P. stemphylioides*, and phylogenetic analysis showed that *P. stemphylioides* is nested in *Pleurotheciaceae*. However, Su et al. (2015) showed that *P. atra* and *P. garethjonesii* belong to the *Pleosporales*. In the future, the discovery of more *Phragmocephala* species and provision of DNA sequences may better explain the differences between *P. stemphylioides* and other *Phragmocephala* species.

The primary challenge in studying of *Savoryellomycetidae* lies in the morphological similarity among genera across different orders, particularly in their sexual morphs, including *Pseudoascotaiwania* (*Fuscosporellales*), *Helicoascotaiwania* (*Pleurotheciales*), and *Ascolacicola*, *Ascotaiwania*, *Canalisporium*, *Savoryella* (*Savoryellales*) (Fallah et al. 1999; Sri-Indrasutdhi et al. 2010; Réblová et al. 2016, 2020; Yu et al. 2023). These genera typically have pyriform to ellipsoidal, lying horizontal to the host surface ascomata with a lateral neck, cylindrical to clavate, pedicellate asci with an apical ring, and fusiform, horizontally septate, central cells larger and brown, ends cells small and hyaline (Chang et al. 1998; Fallah et al. 1999; Dayarathne et al. 2019; Réblová et al. 2020; Yu et al. 2023). As a result, distinguishing these species based solely on morphological characteristics is challenging. Currently, these genera are mainly distinguished based on DNA sequence data. Therefore, the inclusion of comprehensive DNA sequence data is essential to ensure more accurate identifications when studying morphologically similar fungi.

## Supplementary Material

Supplementary_file_Clean.xlsx
